# Multidimensional Neuroprotective Effects of Combined Ubisol-Q10 and Ashwagandha (*Withania somnifera*) in Male and Female 5XFAD Mice

**DOI:** 10.3390/nu18193270

**Published:** 2026-10-05

**Authors:** Keanna Dube, Alex Stoinescu, Julia Sadler, Michelle Truong, Natasha Malakoti-Negad, Maira Chaudhry, Rohan Talukdar, Jerome Cohen, Siyaram Pandey

**Affiliations:** 1 Department of Integrative Biology, University of Windsor, 401 Sunset Avenue, Windsor, ON N9B 3P4, Canada; dube112@uwindsor.ca (K.D.); truong71@uwindsor.ca (M.T.); talukda1@uwindsor.ca (R.T.); 2 Department of Chemistry & Biochemistry, University of Windsor, 401 Sunset Avenue, Windsor, ON N9B 3P4, Canada; stoines@uwindsor.ca; 3 Department of Biomedical Science, University of Windsor, 401 Sunset Avenue, Windsor, ON N9B 3P4, Canada; sadlerj@uwindsor.ca (J.S.); malakot1@uwindsor.ca (N.M.-N.); chaudh54@uwindsor.ca (M.C.); 4 Department of Psychology, University of Windsor, 401 Sunset Avenue, Windsor, ON N9B 3P4, Canada

**Keywords:** Alzheimer’s disease, *Withania somnifera*, coenzyme-Q10, Ubisol-Q10, mitochondrial dysfunction, neuroinflammation, autophagy, neuroprotection, sex differences

## Abstract

Background/Objectives: Alzheimer’s disease (AD) is a multifactorial neurodegenerative disorder characterized by interconnected pathological processes including mitochondrial dysfunction, oxidative stress, impaired autophagic processes, neuroinflammation, neuronal degeneration, and progressive functional decline. Natural health products (NHPs), coenzyme-Q10 (CoQ10) and Ashwagandha (*Withania somnifera*) (ASH), have been previously reported to possess antioxidant, anti-inflammatory, mitochondrial-supportive, neuroprotective, and neurotrophic properties. This work investigates the neuroprotective effects of a bioavailability-enhanced formulation, Ubisol-Q10 (UQ), and ethanolic ASH extract, combined as a “Tonic,” across multiple domains of AD pathology. Methods: The effects of long-term administration were evaluated in male and female 5XFAD mice from 2.5 to 11 months of age. Longitudinal magnetic resonance imaging assessed hippocampal and white matter volumes, a frailty index assessed physiological resilience, Novel Object and Novel Location Recognition testing assessed spatial and non-spatial memory, while post-mortem Nissl, Golgi–Cox, and immunofluorescence evaluated cellular density, dendritic morphology, amyloid pathology, neuroinflammation, autophagy-associated signaling, and astrocytic activity. Results: Across these measures, Tonic treatment preserved brain volumes, reduced amyloid pathology and inflammation, and modulated autophagy-associated pathways. Treatment also preserved dendritic architecture, frailty progression, and novelty-recognition behavior. Females demonstrated particularly pronounced preservation of white matter structure and increased dendritic density, whereas males showed strong modulation of inflammatory and autophagy-associated markers. Astrocytic immunoreactivity also demonstrated sex- and region-specific patterns, suggesting that glial responses may contribute differently to structural preservation in males and females. Conclusions: Together, these findings support that UQ + ASH may provide diverse protection against AD neurodegeneration. The observed sex-specific responses emphasize the importance of incorporating sex into preclinical evaluation of novel interventions for neurodegenerative diseases.

## 1. Introduction

Alzheimer’s disease (AD) represents a profound threat to the sustainability of global and national healthcare infrastructures. As the seventh leading cause of death worldwide as of 2022 [1], AD poses a significant socioeconomic crisis that increases with our aging population. In Canada, approximately 770,000 citizens currently live with a dementia diagnosis, which is projected to double by 2050, thereby imposing an unsustainable strain on both formal medical systems and informal care networks [2] The AD biological continuum develops over decades and cannot be defined solely by the onset of a series of isolated symptoms [3,4]. Instead, the disease transitions from a preclinical, asymptomatic phase characterized by undetectable synapse loss and sequestration of pathological proteins to a prodromal phase of mild cognitive impairment (MCI) and finally to syndromal dementia, where widespread neuronal death and cognitive disintegration become clinically obvious [5,6].

The core of the AD continuum is driven by five interconnected etiologies, defined as the following: accumulation of amyloid-β (Aβ), which are extracellular protein aggregates that disrupt synaptic signaling at the postsynaptic membrane and form β-sheet structures that impair ion homeostasis [7,8]; tau hyperphosphorylation, which causes tau to detach from microtubules and aggregate, disrupting axonal transport and synaptic integrity [9,10,11]; impaired autophagy, caused by failure in the cellular proteostasis, thus preventing the clearance of misfolded proteins and damaged organelles [12]; neuroinflammation, exemplified by chronic activation of microglia and astrocytes, which release pro-inflammatory cytokines and create a self-perpetuating toxic environment [13,14,15]; and oxidative stress, driven by mitochondrial dysfunction where the resulting reactive oxygen species (ROS) damage lipids, proteins, and DNA, furthering neuronal death [16,17].

Beginning the downward spiral of AD pathology is the formation of Aβ plaques, later followed by neurofibrillary tangles (NFTs), which is concentrated primarily in the hippocampus and entorhinal cortex [1,16,18,19,20,21]. Brain atrophy then results from the progressive loss of neuronal density in these areas [22]. The hippocampus and entorhinal areas are mainly responsible for long-term memory consolidation, spatial mapping, and information processing [23]. Therefore, AD-associated topographic loss of brain tissue and the disruption of cholinergic and glutamatergic pathways directly correlate with memory decline seen as issues in creating new memories, spatial navigation, and confusion [24,25,26].

A significant challenge in AD intervention is that these biochemical changes, including synaptic dysfunction, begin up to 20 years before clinical symptoms manifest [4,18,20]. By the time a patient crosses the “threshold of recognition,” defined as the point when memory decline becomes obvious, extensive neuronal death has already occurred [5,6]. This lengthy progression over decades emphasizes the necessity of developing interventions that can interrupt the underlying biological complexities before they develop into irreversible neurological decay. By moving away from prioritizing therapeutic symptomatic management and instead focusing on treating the underlying molecular system, we may be able to interrupt the progression from asymptomatic cellular stress to irreversible cognitive failure.

### 1.1. Sex Differences in AD

AD disproportionately affects women, who account for nearly two-thirds of all diagnosed cases. In the United States, women have an estimated lifetime risk of one in five for developing AD, compared with approximately one in ten for men [27]. Although longer lifespan has traditionally been proposed to explain this uneven distribution, more recent evidence suggests that biological sex itself is an independent risk factor influencing disease susceptibility, progression, and therapeutic response [27,28]. For instance, women diagnosed with AD generally show faster cognitive decline, greater impairment in episodic memory, and more severe neuropsychiatric symptoms than men at similar stages of disease [29]. These symptomatic differences are accompanied by greater accumulation of Aβ and tau pathology, accelerated hippocampal atrophy, and increased synaptic loss [30]. On the other hand, men often demonstrate greater vascular pathology and tend to exhibit more pronounced deficits in visuospatial abilities and executive function [27,28].

Nonetheless, aging still remains the strongest risk factor for AD and is accompanied by numerous biological changes, including mitochondrial dysfunction, cellular senescence, impaired proteostasis, epigenetic alterations, and immune dysregulation [27]. However, the aging process differs substantially between males and females due to differences in sex hormones, metabolism, and immune function. Estrogen is considered a neuroprotective hormone because of its ability to support mitochondrial health, reduce oxidative stress, and prevent Aβ accumulation and tau hyperphosphorylation [31,32,33]. During the menopausal transition, declining ovarian hormone production may diminish these protective effects, thus increasing susceptibility to AD in women. In aging men, testosterone levels also decline, although more gradually than hormonal changes seen in aging women, and this role in cognitive decline and AD remains less understood [34].

A review by Waters et al. (2021) [28] reported that only approximately one-third of preclinical AD studies include both male and females, and fewer than 10% of those directly investigate sex-specific treatment effects or disease mechanisms. As a result, important biological differences that may influence therapeutic efficacy are often overlooked. Incorporating both male and female animals into preclinical studies is therefore necessary for improving the translational relevance of AD research and developing treatments that are effective across both sexes.

### 1.2. Current Pharmacological Interventions

At present, several U.S. Food and Drug Administration (FDA)-approved medications are clinically recommended for the treatment of AD. These include acetylcholinesterase (AChE) inhibitors, such as donepezil, rivastigmine, and galantamine, which increase cholinergic signaling by preventing the breakdown of acetylcholine within the synaptic cleft [35]. N-methyl-D-aspartate (NMDA) receptor antagonists, such as memantine, reduce glutamate-mediated excitotoxicity by limiting excessive calcium influx into neurons [35].

More recently, anti-amyloid monoclonal antibodies, such as lecanemab and donanemab, have been approved as targeted therapies for AD. These engineered antibodies selectively bind to Aβ aggregates and flag them for removal by the immune system [14]. However, since the monoclonal antibody has moved into human trials, the results have been met with some disappointment due to the extremely high cost (~$30,000 USD/year) and only ~30% reduction in AD progression [1,4]. In addition, the currently recommended medications are associated with adverse side effects and toxicity with long-term use, which may worsen overall health and quality of life, especially in older adults [1,4].

In all, the current therapeutic landscape includes moderate-stage symptomatic-management therapies as well as promising disease-modifying anti-amyloid approaches; however, our options still remain limited in the ability to significantly slow or halt the progression of AD [36,37,38]. Perhaps the issue lies in the narrow targeting potential of these pharmaceutical agents, which act largely on downstream pathological effects of AD; therefore, upstream metabolic stressors such as mitochondrial dysfunction or impaired autophagy-related systems may not be addressed [6].

Recently, attention has been given to natural health products (NHPs) as potentially safer, multi-targeting alternatives [39,40,41,42,43,44,45,46,47,48]. Some NHPs offer broad mechanistic benefits with good safety profiles and therefore may be suitable for long-term intervention, especially for conditions that develop over decades, such as AD. To this point, the current study introduces a novel “Tonic” consisting of a combination of two natural health products as a potential therapy to combat AD neurodegeneration, detailed in the proceeding sections.

### 1.3. Ubisol-Q10: Enhanced Bioavailability and Mechanisms of Protection

Coenzyme-Q10 (CoQ10) is a vital electron carrier in the mitochondrial electron transport chain (ETC), facilitating electron transport between Complexes I, II, and III to produce ATP [49,50]. Although conventional CoQ10 possesses considerable antioxidant potential, its lipophilic nature and extensive metabolic processing contribute to limited bioavailability, with only ~5% of orally administered CoQ10 reaching systemic circulation [51]. This has historically led to poor clinical translation due to effective doses being unrealistically high for daily human consumption (1200–2400 mg/kg/day) [52,53].

To address this bioavailability problem, recent research has focused on developing alternative delivery systems designed to improve CoQ10 solubility and stability, enhance gastrointestinal absorption, and facilitate delivery to intracellular or mitochondrial targets [54,55,56]. For example, idebenone is a novel synthetic analog of CoQ10 (ubiquinone) and has been reformulated to be encapsulated in nanocomposite microspheres for delivery via nasal inhalation [57]. The alternative formulation was originally derived for neurodegenerative disease treatment, due to the potential for the 7.37 ± 2.4 μm sized particles to bioadhere to the nasal passage and be delivered more directly to the olfactory region of the brain. However, this novel formulation has only been tested in vitro, and its translated efficacy and enhanced bioavailability in animal models remains speculative.

Similarly, Ubisol-Q10 (UQ), a patented nanomicellar formulation (Micillic Lda, Lisbon, Portugal), was developed by the National Research Council (NRC) and has shown promise in reducing effective dosing as low as 6 mg/kg/day all while retaining its neuroprotective benefits [42,43,44,45,46,47]. UQ combines CoQ10 (equal parts ubiquinone and ubiquinol) with polyoxyethany-α-tocopheryl sebacate (PTS), a synthetic derivative of Vitamin E, in a 1:2 molar ratio. The formulation produces stable micelles with an average diameter of 22 ± 7 nm. This amphiphilic structure allows the CoQ10 to bypass digestive barriers and ensures effective transport to the brain [42,43,44,45,46], increasing cerebral CoQ10 concentrations by approximately 30–50% following oral administration in rats [46,58].

Several other alternative CoQ10 delivery systems have similarly demonstrated enhanced bioavailability in preclinical models. Onoue et al. (2014) [59] administered a nanocrystalline solid dispersion of CoQ10 prepared using a cold wet-milling system to rats at 100 mg/kg, resulting in a 13-fold increase in bioavailability compared with crystalline CoQ10. Similarly, Yang et al. (2017) [60] reported 2.43-fold and 3.0-fold increases in CoQ10 bioavailability following administration of a solid dispersion containing fumed silica in rats at 100 mg/kg. Therefore, UQ’s comparable increase in brain CoQ10 concentration at a low dose of 6 mg/kg supports its potential as a promising bioavailable delivery system for neurological applications [58].

Mechanistically, UQ acts to stabilize the mitochondria by integrating itself into the membrane to regulate ROS and prevent the opening of the mitochondrial permeability transition pore (mPTP) [61]. It also specifically blocks BAX-mediated apoptosis to preserve neuronal viability [62,63]. UQ shows promise as a potential therapeutic for AD because of its ability to enhance ATP production by maintaining neuronal hardiness under metabolic stress [49,50].

### 1.4. Ashwagandha (Withania somnifera) Bioactive Compounds and Mechanisms of Repair

Ashwagandha (ASH) serves as a potent nootropic and pro-neurotrophic agent [22,64]. However, its value shines in its ability to repair neural networks and modulate the inflammatory environment within the AD brain [65,66,67,68,69]. ASH is particularly rich in withanolides, which promote neurite outgrowth and upregulate antioxidant enzymes (SOD, GPX) [65,66]. Withaferin A, specifically, inhibits NF-κB signaling and activates the Nrf2-related antioxidant response [67,68,69].

ASH’s ability to reduce pro-inflammatory cytokines TNF-α and interleukin-1β (IL-1β), clear Aβ via upregulation of low-density lipoprotein receptor-related protein (LRP1) to transport amyloid from the brain to the plasma, and inhibit AChE to enhance cholinergic signaling for improved memory formation make it a strong suitor to address the multi-faceted nature of AD [25,64].

### 1.5. UQ + ASH “Tonic” Complementary Mechanisms Against Neurodegeneration

Considering the potential benefits of UQ and ASH against neurodegeneration, previous work from our research team hypothesised that a combination of the two NHPs, together in a “Tonic” solution, may provide more well-rounded effects than either implemented alone. Therefore, Vegh et al., 2021, 2025, and 2026 [42,43,44], sought to evaluate the outcome of UQ, ASH, and combined Tonic treatment in a double transgenic AD mouse model and paraquat-induced Parkinson’s disease (PD) rat model. Additionally, Dube et al. (2026) [48] comprehensively assessed the literature to create an integrative mechanistic model of possible mechanisms of action of UQ, ASH, and the Tonic against AD and PD biochemical processes.

Nonetheless, the previous reports suggest that these compounds, when combined into the Tonic, do indeed provide complementary neuroprotective actions by targeting distinct but interconnected aspects of neurodegenerative pathology. UQ, as a mitochondrial electron transport chain stabilizer and antioxidant [55,70], may act by supporting mitochondrial function and limiting oxidative stress, with particularly positive effects in the PQ-induced PD model, where mitochondrial dysfunction and oxidative injury are acute drivers of pathology [71]. In contrast, ASH has demonstrated strong effects in the more progressive AD model, consistent with its reported ability to enhance neurotrophic signaling, support astrocytic function, modulate synaptic plasticity, and promote neuronal repair [22,72,73]. Even though each component was found to have distinct strengths within each experimental condition, the combination Tonic consistently produced the most balanced and diverse neuroprotective results overall.

**Figure 1:**
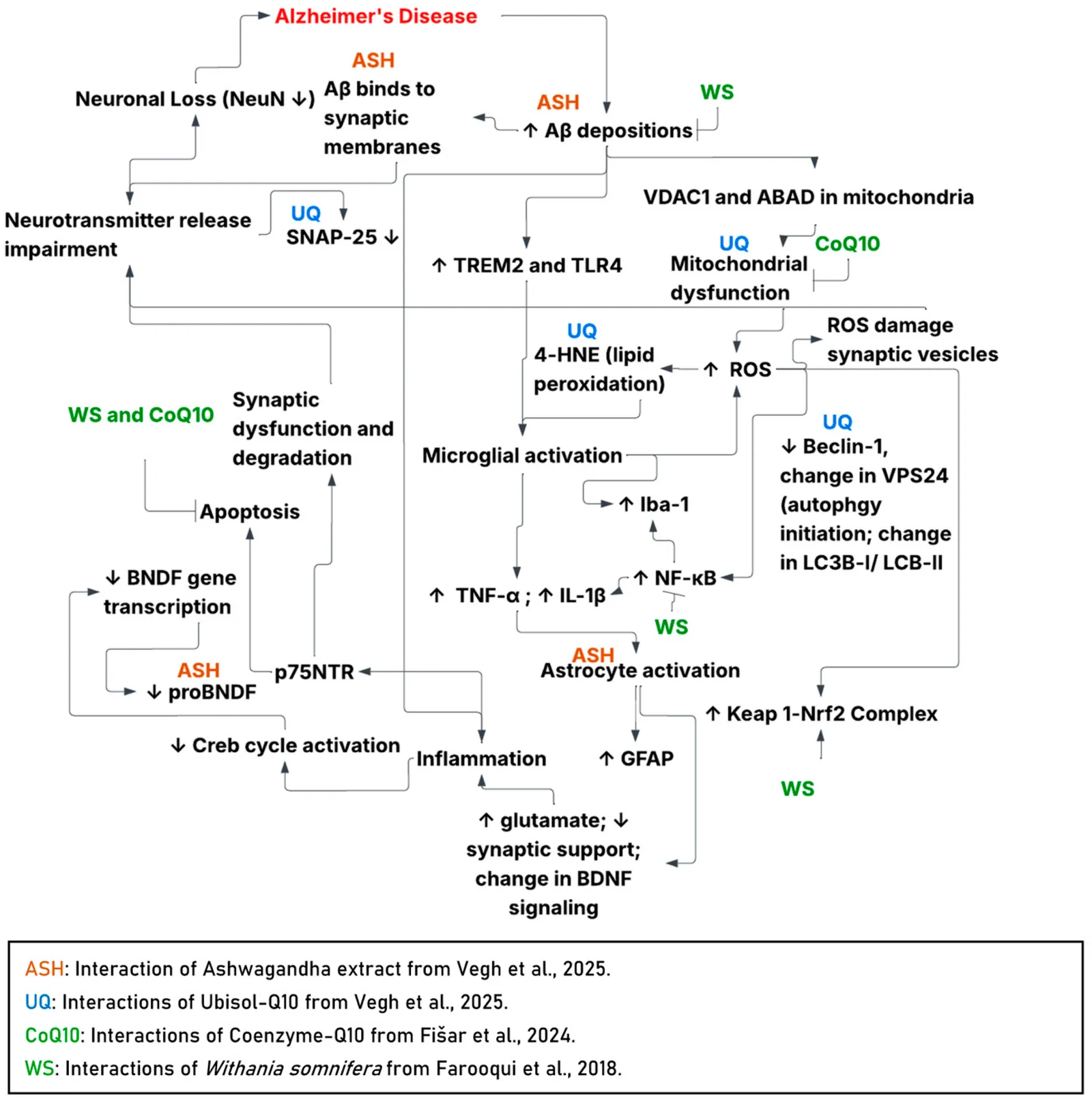
Taken from Dube et al. (2026) [48]. Integrative schematic illustrating the biochemical pathways underlying Alzheimer’s disease (AD, written in red to highlight the disease being exemplified in the current figure) and the mechanistic points of action of the tonic treatment components (Ubisol-Q10 (UQ) and Ashwagandha (ASH)). ASH and UQ (shown in orange and blue, respectively) represent findings of past published work from our lab [43] and indicate their reported points of influence within AD-related biochemical pathology. Coenzyme-Q10 (CoQ10) and *Withania somnifera* (WS), shown in green, represent findings from external studies [74,75]. Detailed statistical analysis should be consulted from the original publications. Up and down arrows next to each protein represent the disease-induced direction of expression typically seen for that marker.

Figure 1 below is taken from Dube et al. (2026) [48] and details the proposed mechanistic interactions of UQ and ASH throughout AD-related biochemical processes.

### 1.6. Synthesis of Study Objectives and Multi-Disciplinary Approach

Altogether, the previous literature on UQ, ASH, and Tonic gives promising evidence that the combination treatment may provide diverse effects against neurodegeneration, such as mitochondrial protection, antioxidant defense, neurotrophic signaling, glial regulation, improved autophagy-related processes, and neuronal repair rather than targeting a single pathological pathway [42,43,44,48]. Thus, the current study aims to further evaluate the Tonic’s potential in a stringent 5XFAD mouse model of AD.

We would like to emphasize throughout this work that our fundamental aim is to evaluate the Tonic’s efficacy across several timepoints of the degenerative process and across several disease-related domains involved in the aggressive 5XFAD genetic mimic of AD. Therefore, our experimental design uniquely follows the mice from 2 months of age to 11 months of age and comprehensively utilizes multiple disciplinary assessments to confirm the safety and efficacy of this treatment. Therefore, the Tonic was evaluated via pre-mortem longitudinal magnetic resonance imaging (MRI) to track the prevention of brain atrophy and white matter degradation, frailty index assessment to evaluate physiological well-being as the mice age, repeated spatial and non-spatial memory tests in an X-maze, and post-mortem biochemical assays to analyze synaptic maturity via Golgi–Cox staining, cellular density via Nissl staining, and the suppression of astrocyte/microglia activation and amyloid pathological markers and promotion of autophagy-associated signaling via immunofluorescent assays. Moreover, we understand the previous literature from our research team involved only male animals. Henceforth, the current study hopes to advance our understanding of the Tonic’s therapeutic outcomes in both male and female mice across all experimental domains and aims to deduce if the treatment possesses potential sex-dependent effects throughout AD development.

## 2. Materials and Methods

### 2.1. ASH Extraction and Phytochemical Characterization

ASH powder (obtained from Premier Herbal Inc., North York, ON, Canada; LOT No. ARP1812AI) was extracted using an anhydrous ethanol protocol adapted from Vegh et al. (2021) [46]. The extract was filtered through P8 filter paper, concentrated by rotary evaporation, and reconstituted in anhydrous ethanol to a final concentration of 200 mg/mL.

Phytochemical characterization was performed by Laboratoire PhytoChemia (Chicoutimi, QC, Canada). Withanolide composition was quantified using ultra-performance liquid chromatography with ultraviolet detection (UPLC-UV), and total flavonoid content was measured using a Dowd’s reagent colorimetric assay. The phytochemical composition of the extract is summarized in Table 1 (reproduced from Vegh et al. 2021 [46]).

### 2.2. Animal Care and Experimental Design

All procedures were approved by the University of Windsor Animal Care Committee (AUPP#22-11) and conducted in accordance with the Canadian Council on Animal Care (CCAC) guidelines. Wild-type (B6129SF2/J) and transgenic 5XFAD mice (B6.Cg-Tg(APPSwFlLon, PSEN1*M146L*L286V)6799Vas/Mmjax, RRID: MMRRC_034848-JAX) [76] were obtained from the Mutant Mouse Resource and Research Center (MMRRC) at The Jackson Laboratory, an NIH-funded strain repository, and donated to the MMRRC by Robert Vassar, Ph.D., Northwestern University. The 5XFAD model expresses five familial AD-associated mutations: APP Swedish, Florida, and London, together with PSEN1 M146L and L286V mutations.

Twenty-three male and twenty-two female mice were randomly assigned to three experimental groups: Tonic-treated 5XFAD (50 μg/mL UQ + 2 mg/mL ethanolic ASH extract; 8 mg/kg/day UQ + 320 mg/kg/day ASH; *n* = 8 males, 7 females), untreated 5XFAD controls (*n* = 8 males, 7 females), and wild-type controls (*n* = 7 males, 8 females). Control animals received the vehicle solution (0.01% PTS and 1% ethanol). Please note that previous reports by our research team have reported that Tonic treatment consistently provided the most balanced therapeutic outcomes when compared to independently treated ASH and UQ groups [42,43,44,48]. Therefore, we deemed it reasonable to proceed with testing the Tonic treatment alone for this study.

Treatment concentrations were calculated from average daily water consumption (~4 mL/mouse/day), and water intake was monitored throughout the study to ensure consistent dosing across animals. Mice were housed in groups of four under standard laboratory conditions at 18–23 °C and 40–70% humidity with ad libitum access to food (Purina LabDiet Rodent 5001 Chow; North American Lab Supply, Forth Worth, TX, USA) and their designated treatment/control solution, provided in their home cage water bottles. Mice were placed on a semi-restricted diet only during times of cognitive testing in the X-maze. Details regarding this feeding regime are explained below. Animals were separated only when necessary, due to persistent aggression. Three animals died from causes unrelated to the study and were excluded from subsequent analyses (*n* = 1 male wild-type/control, 1 female 5XFAD/control, 1 female 5XFAD/Tonic).

**Figure 2:**
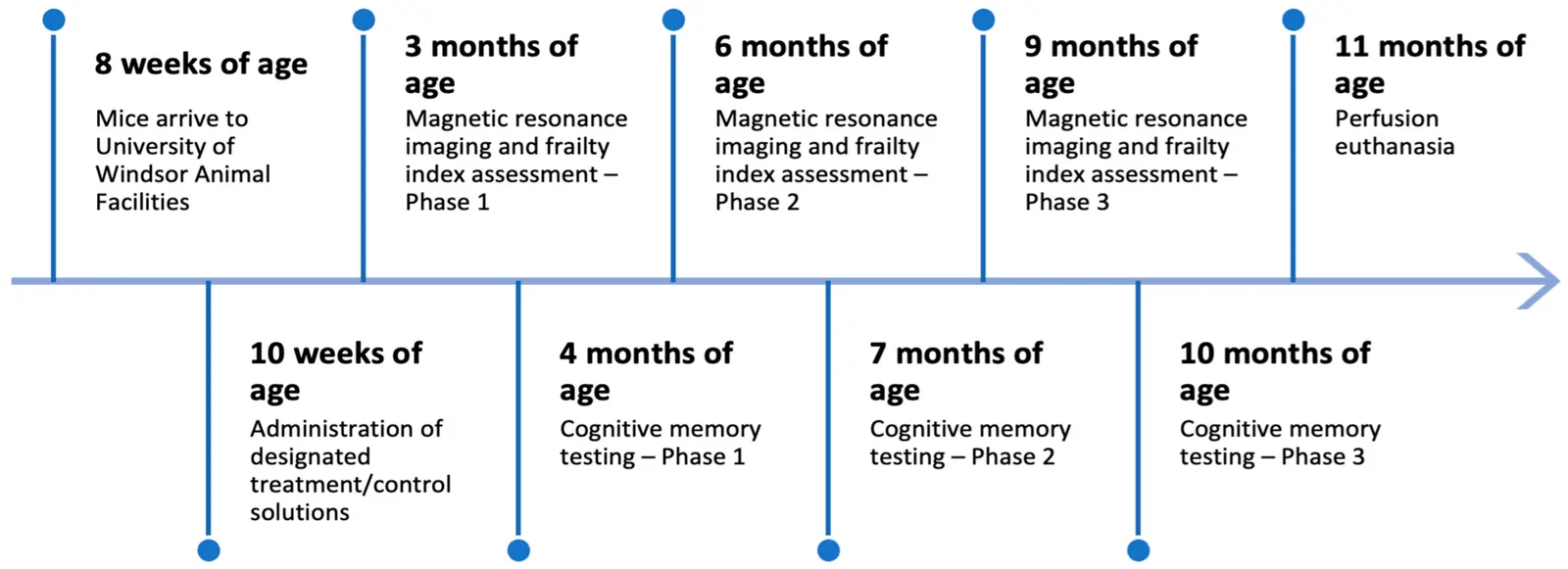
Experimental timeline following the 45 mouse subjects used in the current study.

Mice arrived at the University of Windsor animal facility at 2 months of age with a two-week acclimation period before beginning treatment administration at 10 weeks of age. MRI and behavioral/cognitive testing started at 12 weeks of age and continued until euthanasia at 11 months. Investigators responsible for animal handling, behavioral testing, and subjective assessments remained blinded to treatment allocation throughout the study. Figure 2 shows the timeline of pre-mortem experimental events and end point of the study over the mice’s lifetime.

### 2.3. MRI

The current study used a T2-weighted 3-dimensional Fast Spin Echo (T2W 3D-FSE) MRI technique. This method was used because of MRI’s non-invasive manner and high soft-tissue contrast for the differentiation of white matter, gray matter, and cerebrospinal fluid (CSF) to track progression of integrity loss in tissue from early- to late-stage degeneration.

In this study, we will focus on four brain regions: hippocampus, fimbria, anterior commissure of olfactory, and corpus callosum. Although AD has traditionally been associated with gray matter degeneration, increasing evidence suggests that white matter pathology is also a major feature of disease progression [77,78,79]. White matter tracts are essential for communication between brain regions and are particularly vulnerable to chronic neuroinflammation [80]. Inflammatory mediators released by activated glial cells can impair oligodendrocyte function and damage myelin, ultimately degrading white matter integrity and disrupting neuronal connectivity. The fimbria and anterior commissure of olfactory show early structural changes associated with Aβ pathology and memory impairment, and degeneration of the corpus callosum supports the myelin breakdown hypothesis, which proposes that smaller myelinated fibers are especially susceptible to age-related and neurodegenerative damage [77,78,79].

Therefore, the current study aims to track treatment effects on brain tissue integrity throughout the degenerative process and explore potential early-degenerative signs via longitudinal volumetric assessment of the hippocampus, fimbria, anterior commissure of olfaction, and corpus callosum.

#### 2.3.1. In Vivo MRI Acquisition

All animals were imaged at 3, 6, and 9 months of age. Prior to imaging sessions, mice were transported from the University of Windsor Animal Care Facility to the MRI facility in temperature-controlled transport containers to maintain stable environmental conditions. For imaging procedures, animals were anesthetized using 3% isoflurane delivered in oxygen at a flow rate of 1 L/min during induction. Adequate anesthetic depth was confirmed by loss of the withdrawal reflex before initiating imaging. Once anesthesia was achieved, ocular lubricant was applied to protect the eyes during the procedure, and the head was secured using a cotton-Velcro brace designed for finger strains, to reduce movement during image acquisition.

Animals were positioned within the MRI bed and inserted into a 6 cm diameter radiofrequency coil. Thermal support was provided using insulated padding and a temperature-controlled support system to maintain physiological body temperature throughout imaging. During scanning, anesthesia was maintained at approximately 2–2.5% isoflurane without mechanical ventilation. Physiological parameters were monitored continuously using MRI-compatible equipment. Heart rate and respiratory activity were recorded using external sensors, and body temperature was measured with a rectal fiber-optic temperature probe. Body temperature was maintained at approximately 37 °C throughout image acquisition. At completion of imaging, mice were transferred to a recovery chamber with supplemental oxygen until spontaneous movement and normal behavior resumed before being returned to their home cage.

#### 2.3.2. MRI Acquisition Parameters

MR images were collected using the M2™ Compact High-Performance MRI System (Aspect Imaging Ltd., Shoham, Israel) with a magnetic field strength of 1 Tesla. The T2W 3D FSE sequence was adapted from previously published protocols in transgenic AD mouse models [81]. Table 2 details the exact parameters for male and female mice.

#### 2.3.3. MRI Image Processing and Segmentation

Following acquisition, MRI datasets underwent post-processing and quantitative analysis. Raw DICOM image files were first processed using MATLAB R2025a (MathWorks, Natick, MA, USA), where skull stripping was applied using a customized unbiased template. Signal normalization was then performed by scaling image intensity to the maximum CSF signal intensity for each subject. This normalization procedure reduced variability in image intensity between slices and across longitudinal scans to improve consistency of quantitative measurements.

**Figure 3:**
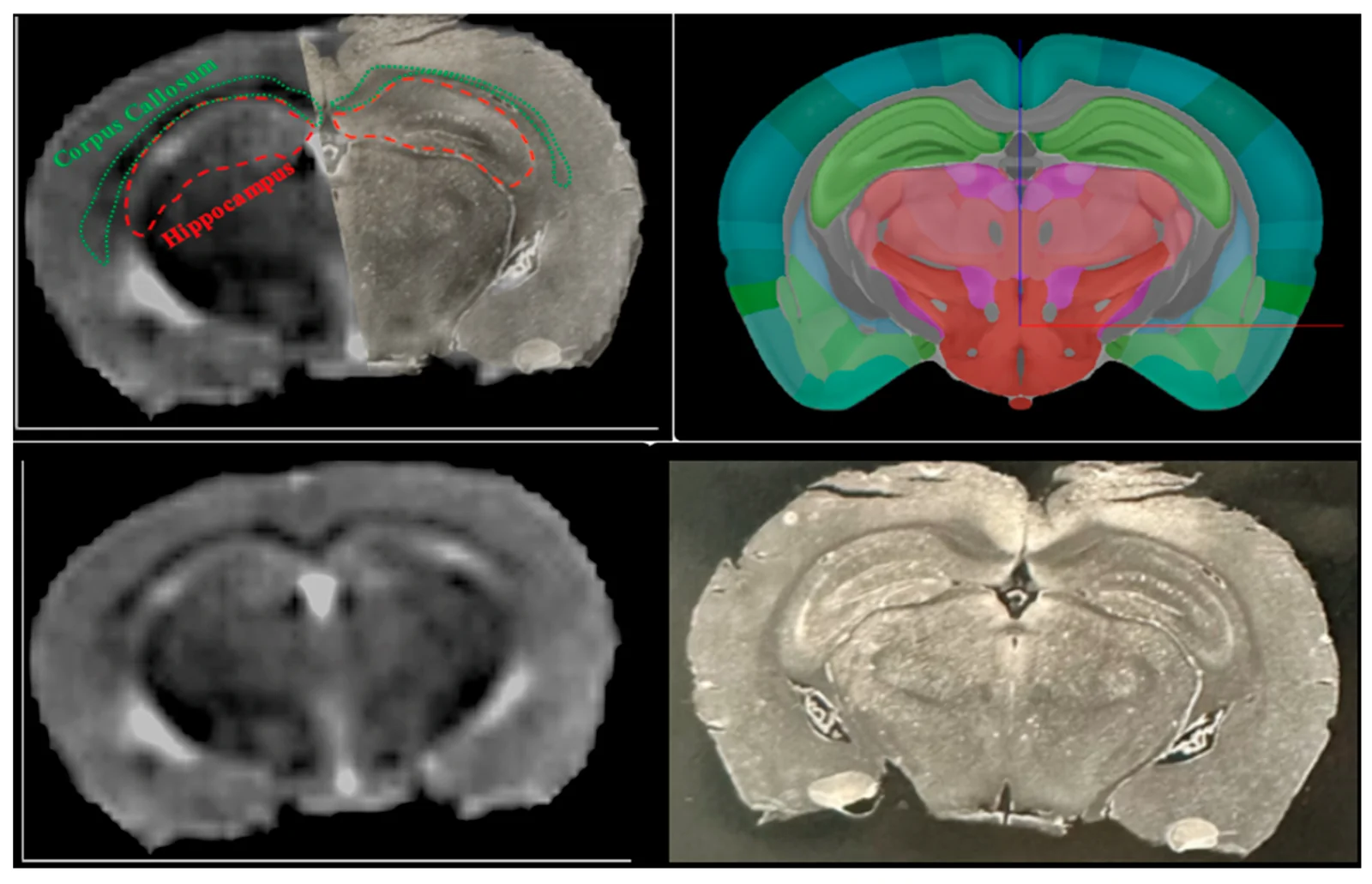
Example of how the Allen Mouse Brain Atlas (https://mouse.brain-map.org/experiment/show/71717640, accessed on 7 August 2024) (top right) and sectioned brain tissue (bottom right) were used in conjunction with magnetic resonance images (MRI) (bottom left) to segment the hippocampus and corpus callosum (top left). Photos of brain tissue sections were taken from microscope-mounted cryosectioned brains (30 µm thickness) of the mouse subjects used in the current study. The brain regions from the mounted brain section and Allen Mouse Brain Atlas shown here were selected because they aligned with the MRI slice well and could be overlayed to provide guidance for segmenting the brain region of interest (ROI).

Image segmentation and volumetric analysis were completed using 3D Slicer 5.2.2 (Slicer Community, 2023, Boston, MA, USA). Anatomical templates were generated using reference coordinates from the Allen Mouse Brain Atlas [82,83] along with microscope slide-mounted brain sections (30 µm thickness) from a mouse used in the current experiment to guide region selection and maintain consistency between animals. Segmentation was performed manually using image tracing tools and reviewed using 3D reconstruction to confirm anatomical accuracy. Figure 3 gives an example of how tissue sections and atlas regions were used to guide manual segmentation. Statistical analyses were performed using GraphPad Prism 11. Differences between experimental groups were evaluated using two-way repeated measures analysis of variance (ANOVA), with experimental group as fixed between-subject factors and time as variable. The Geisser–Greenhouse correction was applied when the assumption of sphericity was violated. Significant main effects or interactions were followed by Bonferroni-adjusted multiple-comparisons tests. All tests were two-tailed, with statistical significance defined as *p* < 0.05. Data are reported as mean ± SEM.

### 2.4. Post-Mortem Tissue Preparation for Histochemical and Immunofluorescent Assays

At 11 months of age, mice were deeply anesthetized with 3% isoflurane (1.5 L/min O_2_) and transcardially perfused with ice-cold phosphate-buffered saline (PBS) containing 28 μg/mL heparin, followed by 10% formaldehyde in PBS. Brains were removed and post-fixed at 4 °C. For the males, half the subjects’ brains were immediately prepared in Golgi–Cox staining solution after perfusion to adjust for minor interactions of post-fixation on staining efficacy found during the procedure with female mice. To prepare for biochemical assays, brains were cryoprotected in 30% sucrose until fully submerged, embedded in Shandon™ M-1 embedding matrix (Thermo Scientific, Mississauga, ON, Canada), and cryosectioned coronally at 30 μm or 14 μm for subsequent histological and immunohistochemical analyses.

### 2.5. Nissl Staining

Nissl staining was performed to corroborate MRI volumetric findings and deduce if volumetric findings were consequence of changes to cell density or some other matter. For this, we used a cresyl violet staining protocol adapted from the User Manual (PS102, Version 2011-1) FD Cresyl Violet Solution™ (FD NeuroTechnologies, Cat. #PS102-01, Columbia, MD, USA) manufacturer’s recommendations. Light micrograph images of Nissl-stained sections were acquired at 10× and 20× magnification.

Nissl staining within the corpus callosum (at 20× magnification) was quantified using ImageJ (NIH, Bethesda, MD, USA). Three unique animals per group were randomly selected for assessment. Microscope slides contained three slices per slide, so three fields were analyzed per subject for statistical analysis and interpretation. Brightfield images were converted to 8-bit grayscale, and the corpus callosum was manually outlined using the freehand selection tool to define the region of interest (ROI). A threshold was applied to identify cresyl violet-positive staining while excluding unstained background tissue. The thresholded area within the ROI was then measured using the Area Fraction function, which calculates the percentage of the total ROI occupied by cresyl violet-positive staining. The resulting percentage area was used as a quantitative measure of Nissl staining density within the corpus callosum. Statistical analyses were performed using Microsoft Excel Version 16.111.1. Differences between groups were initially assessed using a one-way analysis of variance ANOVA to determine overall group effects. When significant differences were detected, pairwise comparisons between groups were performed using two-tailed independent samples *t*-tests assuming unequal variance (Welch’s *t*-test) in Microsoft Excel. Comparisons were performed between Tonic-treated 5XFAD, 5XFAD control, and wild-type control groups. To account for multiple comparisons, Bonferroni correction was applied by multiplying the unadjusted *p*-value by the total number of comparisons performed (*n* = 3; 5XFAD/Tonic vs. 5XFAD/Control, 5XFAD/Tonic vs. Wild-Type/Control, and 5XFAD/Control vs. Wild-Type/Control). Adjusted *p*-values were considered statistically significant when *p* < 0.05. Data are presented as mean ± SEM.

### 2.6. Immunohistochemistry

The immunofluorescent assay methodology is a critical component of this research, which is designed to provide biochemical validation of the Tonic’s ability to target the multifactorial etiologies of AD. While longitudinal MRI tracks macrostructural changes like brain atrophy, immunofluorescence allows for microscopic mechanistic analysis of how these treatments modulate pathological pathways. Here, we will look at several biomarkers to evaluate the Tonic’s multi-targeting potentials via immunofluorescent assays for Aβ pathology, chronic neuroinflammation characterized by the sustained activation of microglia and astrocytes, and impaired autophagy-associated systems and proteostatic homeostasis. Table 3 summarizes the markers used and their intended purpose for this study.

Results from these biochemical assays will be used to corroborate MRI findings. For instance, if MRI shows enlarged white matter volumes, immunofluorescence (and Nissl staining) can determine if this is due to inflammatory edema, reactive gliosis, or abnormal myelin remodeling or if it is truly healthy tissue growth [77,78,84]. The analysis methods proposed here ensure that structural preservation observed in vivo is indeed secondary to the suppression of molecular pathological cascades.

#### 2.6.1. Primary Antibodies

Immunofluorescent staining was performed using seven primary antibodies: mouse anti-β-amyloid monoclonal antibody (1:500; Santa Cruz Biotechnology, Dallas, TX, USA; Cat. No. sc-28365) in combination with rabbit anti-amyloid precursor protein (APP) monoclonal antibody (1:500; Abcam, Dallas, TX, USA; Cat. No. ab32136), goat anti-interleukin-1β (IL-1β) polyclonal antibody (1:250; Novus Biologicals, Dallas, TX, USA; Cat. No. AF-401-SP) paired with rabbit anti-Beclin-1 polyclonal antibody (1:200; Novus Biologicals; Cat. No. NB500-249SS), rat anti-ionized calcium-binding adapter molecule 1 (Iba1) monoclonal antibody (1:300; Abcam; Cat. No. ab283346) paired with rabbit anti-microtubule-associated protein 1 light chain 3B (LC3B) monoclonal antibody (1:250; Abcam; Cat. No. ab192890), and rabbit anti-α-glial fibrillary acidic protein (α-GFAP) polyclonal antibody (1:250; Novus Biologicals; Cat. No. NB300-141).

#### 2.6.2. Immunofluorescence Staining Procedure

Three microscope slides from each experimental group containing three hippocampal tissue sections per slide were randomly selected for staining. The average of three fields per mouse was used as the biological replication for statistical analysis and interpretation of immunofluorescence outcomes. Slides were initially rinsed twice in 1XTris-buffered saline (TBS) for 5 min per wash. Antigen retrieval was performed using Historeveal (Abcam; Cat. No. ab103720), with incubation times adjusted according to tissue thickness (1 min for 30 μm sections and 20 s for 14 μm sections). Slides were subsequently washed twice with TBS. To reduce nonspecific binding, tissue sections were incubated with DAKO Universal Protein Block (DAKO, Dallas, TX, USA; Cat. No. X0909) for 15 min. Primary antibody solutions were prepared using Tween (1:100), DAKO antibody diluent (Agilent Technologies Canada Inc.; Cat. No. S0809), and the appropriate antibody dilution to a final volume of 400 μL. After gentle mixing, 100 μL of solution were applied to each section and incubated overnight at 4 °C. The following day, slides were washed twice with TBS for 5 min each before secondary antibody incubation. Secondary antibody solutions were prepared in antibody diluent with Tween (1:100) to a final volume of 400 μL. Sections received 100 μL of secondary antibody solution and were incubated for 2 h. Following incubation, slides were washed twice with TBS and mounted using VECTASHIELD^®^ Vibrance^®^ antifade mounting medium (MJS BioLynx Inc., Brockville, ON, Canada; Cat. No. VECTH17002 or VECTH18002 with DAPI). Coverslips were applied, and slides were allowed to cure for 1 h or overnight before imaging. Images from 30 μm sections were acquired using a Leica DMI6000 B inverted microscope (Leica Microsystems, Concord, ON, Canada).

#### 2.6.3. Immunofluorescence Staining Quantitative Analysis and Statistical Testing

Immunofluorescence images were quantified using ImageJ (NIH, Bethesda, MD, USA). Individual fluorescence channels were analyzed separately and converted to 8-bit grayscale. Background fluorescence was reduced using the Subtract Background function with a rolling ball radius of 50 pixels. A consistent threshold was then applied to identify positive fluorescent signal while excluding background fluorescence. The percentage of the image area occupied by fluorescence was quantified using the Area Fraction measurement, which calculates the proportion of pixels within the ROI exceeding the defined threshold. The resulting percentage area was used as a quantitative measure of marker expression. Images from all experimental groups were analyzed using identical background subtraction and threshold settings to ensure consistency across samples. Differences among experimental groups were initially assessed using one-way ANOVA for overall group differences in Microsoft Excel 16.111.3. Significant ANOVA effects were followed by Bonferroni-adjusted Welch’s *t*-tests for pairwise between-group comparisons. All tests were two-tailed, with statistical significance defined as *p* < 0.05. Data are reported as mean ± SEM.

### 2.7. Golgi–Cox Staining and Tissue Processing

Synaptic loss is one of the strongest structural correlates of cognitive decline in AD, often preceding actual neuronal death [85,86,87,88,89,90]. In AD models, the progressive loss of dendritic complexity disrupts hippocampal function and weakens long-term potentiation [91,92]. Therefore, evaluating morphological features of neurons in the hippocampus using Golgi–Cox methods can determine if performance on spatial and non-spatial memory tasks is supported by a corresponding quality of dendritic complexity and synaptic integration [91,92]. With this procedure, we will quantify dendritic length, thickness, and spine maturity to evaluate if the Tonic can create a favorable environment for neuronal maintenance, remodeling, and maturation of communicatory pathways [58,65,73].

#### 2.7.1. Tissue Impregnation and Golgi–Cox Staining Procedure

Golgi–Cox staining was performed using a dichromate-based impregnation protocol to visualize neuronal morphology. Protocol was obtained and followed according to the manufacturer’s protocol from FD Rapid GolgiStain™ Kit (FD NeuroTechnologies, Ellicott City, MD, USA [93]).

For female mice, 4–8-month-old post-fixed brains were used for the procedure, and for males, fresh perfused brains were used to avoid excessive glial cell staining with post-fixation. After stabilization, tissues were embedded using Shandon™ M-1 embedding matrix (Thermo Scientific, Mississauga, ON, Canada). Samples were then mounted onto specimen discs within the cryostat and secured using Milli-Q before freezing. Coronal sections were cut at a thickness of 100 μm using a cryostat maintained at approximately −23 °C. Tissue sections were mounted onto gelatin-coated glass microscope slides with three sections per slide. Coverslips were mounted using Eukitt quick-hardening mounting medium (Sigma-Aldrich, Oakville, ON, Canada; Cat. No. 03989). Image acquisition was performed as soon as possible following staining to maximize visualization quality. Brightfield imaging was completed using a Leica DMI6000B inverted microscope (Leica Microsystems, Concord, ON, Canada).

#### 2.7.2. Golgi Staining Quantitative Analysis and Statistical Testing

Three animals per group were randomly selected for assessment. Microscope slides contained three slices per slide, and thus three imaging fields per subject were used for statistical analysis and interpretation. Quantitative analysis of dendritic morphology was performed using ImageJ 1.54g software (NIH, Bethesda, MD). Images were calibrated using a 100 µm scale bar to ensure accurate measurement of neuronal structures. Dendritic parameters were assessed based on established Golgi analysis methods described by Risher et al. (2014) [94], with modifications for the current experimental design. Modifications include only visual assessment of dendritic spine maturity based on known morphology rather than taking proportion of width/height.

**Figure 4:**
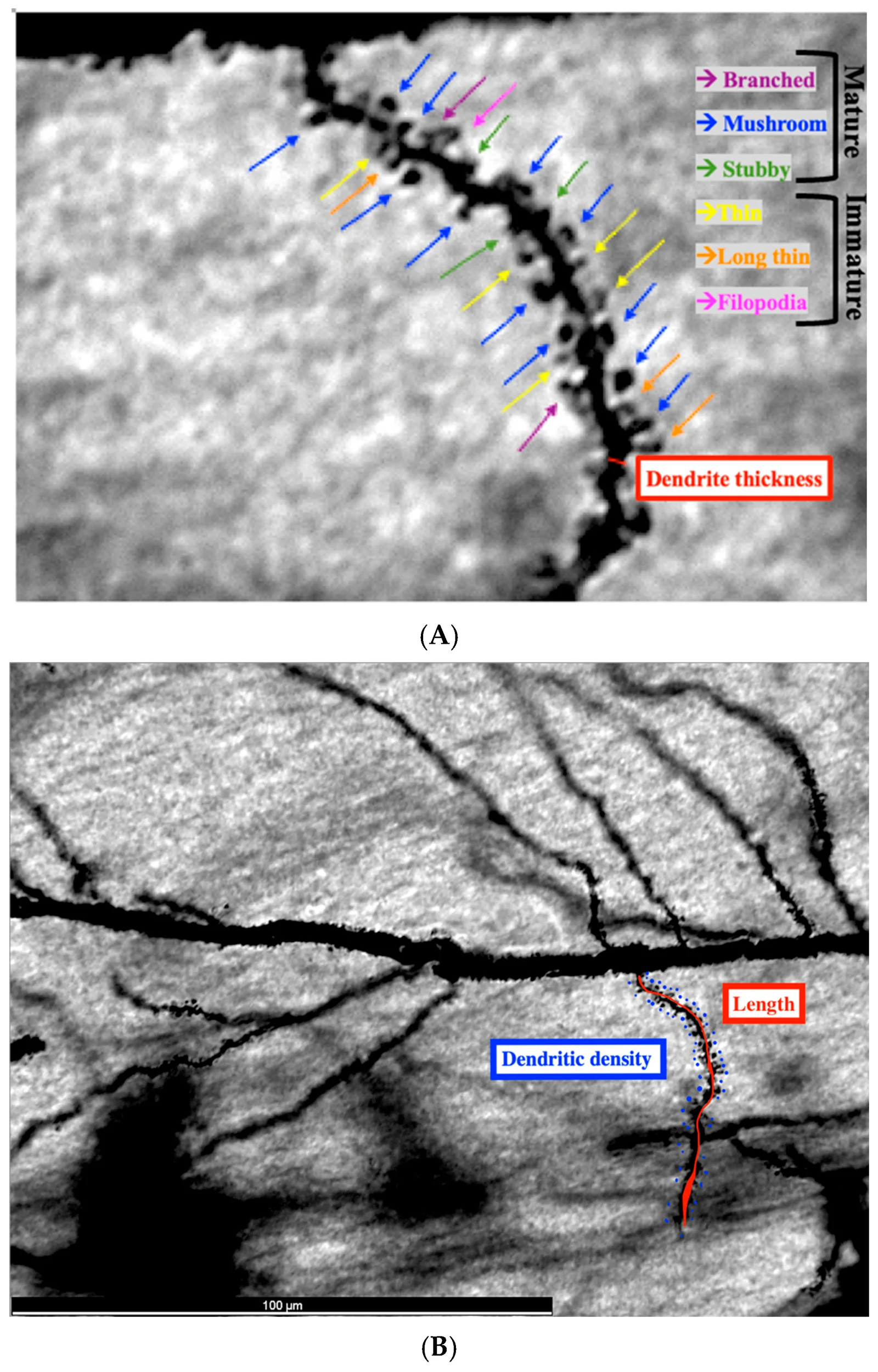
Representative images of Golgi–Cox assessment methodology for (**A**) dendritic spine maturity, dendrite thickness, (**B**) dendrite length, and dendritic spine density. Representative images are taken from 40× magnification brightfield microscope images, and analysis was done using ImageJ. Colors of the representative drawings correspond to the color of their text label within the picture.

Individual secondary dendritic segments were manually traced in ImageJ to quantify dendritic length, dendritic thickness, and dendritic density. Dendritic length was determined by measuring the total traced length of individual dendritic branches, while dendritic thickness was calculated from measurements of dendritic diameter along traced segments. Dendritic density was evaluated by quantifying the number of dendritic branches and/or protrusions within defined dendritic regions. For dendritic spine analysis, individual spines were classified according to established morphological criteria into six categories representing progressive stages of spine development and maturity: filopodia, long thin, thin, stubby, mushroom, and branched spines. Spine classifications were based on morphological characteristics including spine neck length, head size, and degree of branching as described by Risher et al. (2014) [94]. The observed relative proportion of each spine subtype was calculated for each experimental group, with an average of three dendritic spine assessments per subject used as biological replicates, to assess alterations in dendritic spine maturation and synaptic structural plasticity. Figure 4A,B provides a visual example of how each method of assessment described in this section was assessed.

Statistical analyses were performed using Microsoft Excel Version 16.111.1. Differences in dendritic morphology parameters and spine subtype distributions among wild-type control, 5XFAD control, and Tonic-treated 5XFAD groups were initially assessed using a one-way analysis of variance (ANOVA) to determine overall group effects. When significant differences were detected, pairwise comparisons between groups were performed using two-tailed independent samples *t*-tests assuming unequal variance (Welch’s *t*-test) in Microsoft Excel. Comparisons were performed between Tonic-treated 5XFAD, 5XFAD control, and wild-type control groups. To account for multiple comparisons, Bonferroni correction was applied by multiplying the unadjusted *p*-value by the total number of comparisons performed (*n* = 3; 5XFAD/Tonic vs. 5XFAD/control, 5XFAD/Tonic vs. wild-type/control, and 5XFAD/control vs. wild-type/control). Adjusted *p*-values were considered statistically significant when *p* < 0.05. Data are presented as mean ± standard error of the mean (SEM).

### 2.8. Frailty Assessment

Previous research shows that AD degeneration accelerates physical frailty along with cognitive impairments [95]. By assessing these parameters in pre-clinical studies using animal models of AD, we can evaluate how the burden of physical decline, and thus quality of life, may be alleviated by novel therapies, such as our Tonic. Therefore, we aim to employ the 31-item mouse frailty index by Whitehead et al. (2014) [96] to assess how our combination nutraceutical therapy may prevent physical symptoms of AD in conjunction with cognitive and neurostructural measures. Of equal importance, the frailty index is non-invasive, easy to implement, and therefore allows for quantification of physical condition over repetitive assessments over the mouse’s lifetime.

#### 2.8.1. Frailty Index Scoring

Frailty was quantified using a modified version of the mouse frailty index developed for aging and neurodegeneration research [97,98,99]. Frailty was evaluated every three months over the study period using a modified mouse frailty index, adapted from previously validated rodent frailty assessment methods. Assessments were completed prior to any fasting or other experimental manipulations to minimize acute stress effects. The mouse frailty assessment incorporated 31 non-invasive health variables spanning multiple physiological domains, including integumentary, musculoskeletal, vestibulocochlear, ocular/nasal, respiratory, digestive/urogenital, and behavioral systems.

Each deficit was assigned one of 5 scores according to severity: 0 indicating absence of a deficit, 0.25 indicating emerging impairment, 0.50 indicating mild impairment, 0.75 indicating moderate impairment, and 1 indicating severe impairment. Individual scores were summed to generate a frailty score ranging from 1 to 31, where higher values represented greater physiological decline. All observations contributed to the overall frailty score and were used to characterize progression of age- and disease-associated physiological decline across experimental groups.

Table 4 provides a summary of frailty index criteria, their definitions, and how we determined the associated scores. The table is reproduced from Whitehead et al. (2014) [96].

#### 2.8.2. Statistical Analysis of Frailty Index Data

Frailty index data was used to evaluate differences in physical frailty between experimental groups over the longitudinal assessment period. Statistical analyses were performed using GraphPad Prism 11.

To assess differences in frailty scores between wild-type, 5XFAD control, and Tonic-treated 5XFAD groups at each assessment timepoint, a two-way repeated measures ANOVA with time as the variable within-subject factor and experimental group as the fixed between-subject factor was performed to determine whether significant overall differences existed among groups. When a significant group or group × time effect was identified, a two-tailed Bonferroni-corrected multiple-comparisons test was conducted to identify specific group differences. Adjusted *p*-values < 0.05 were considered statistically significant. All frailty index values are presented as mean ± SEM.

### 2.9. Spatial and Non-Spatial Memory Analysis Using the NLR and NOR Tests

Longitudinal novelty-recognition testing in a four-arm radial maze was used to determine whether preservation of neuronal architecture (as evaluated via Golgi–Cox staining) by the Tonic treatment translated into preserved cognitive function. The cognitive task uses repeated habituation followed by novelty-induced dishabituation to assess learning, memory, and recognition processes relevant to AD based on procedures by Benice & Raber (2008) [125]. Across 5 consecutive habituation days, mice repeatedly encountered the same maze configuration and object arrangement to allow mice to become familiar with the environment and objects within. Progressive reductions in exploration across repeated exposures provides evidence of habituation and the formation and retention of a familiar representation [126,127]. The sixth day introduced spatial and object novelty, requiring animals to detect a change from the previously learned configuration. Distinctly different exploration patterns when exposed to the novel stimuli therefore reflects dishabituation and recognition of a previously encountered environment or object, rather than generalized exploratory activity [126,127].

This design also allows spatial and non-spatial recognition memory to be examined separately. Novel Location Recognition (NLR) requires detection of a change in the spatial relationship between a familiar object and its environment, whereas Novel Object Recognition (NOR) requires discrimination based on object identity [125]. Thus, NLR primarily challenges spatial/contextual representations, while NOR assesses recognition of object identity, which can allow measure of cognitive functions that may be differentially affected during AD progression [128,129,130]. The longitudinal design further assesses whether these memory processes were maintained across the disease lifetime (4, 7, and 10 months of age).

#### 2.9.1. Maze Configuration and Feeding Regimen

Testing was performed in a 60 × 60 cm four-arm radial maze. One arm contained a distinctive blue-and-white striped distal visual cue, while each arm opening contained unique proximal visual and textured cues. A double-barrel food cup was positioned at the distal end of each arm, with an accessible Cheerio fragment in the upper cup and an inaccessible Cheerio beneath to standardize olfactory cues. Food rewards encouraged complete maze traversal and maintained exploratory motivation across repeated trials. Maze exploration was recorded using an overhead infrared Canon camera and analyzed using Noldus EthoVision XT 17.0 software.

During behavioral testing, mice were maintained on a semi-restricted feeding schedule of approximately 4 g of food/mouse/day, with adjustments made according to veterinary recommendations and animal welfare monitoring. Food was provided following maze testing, and uneaten food was removed approximately 30 min before testing the following day.

#### 2.9.2. Habituation and Recognition Testing Procedure

Cognitive-behavioral assessment was performed at 4, 7, and 10 months of age, with each evaluation consisting of 5 habituation days followed by the testing day. During habituation, mice completed 4 × 3 min trials per day in an unchanged maze configuration. Three similarly sized objects (approximately 3 × 3 in) were positioned in three arms, with one arm remaining empty. Object identities and locations remained constant throughout habituation. Mice were placed in the central area and allowed to explore freely for 3 min, followed by a 2 min inter-trial interval for maze cleaning and resetting. Maze surfaces and objects were sanitized thoroughly using Prevail cleaning solution to minimize odour-based cues.

**Figure 5:**
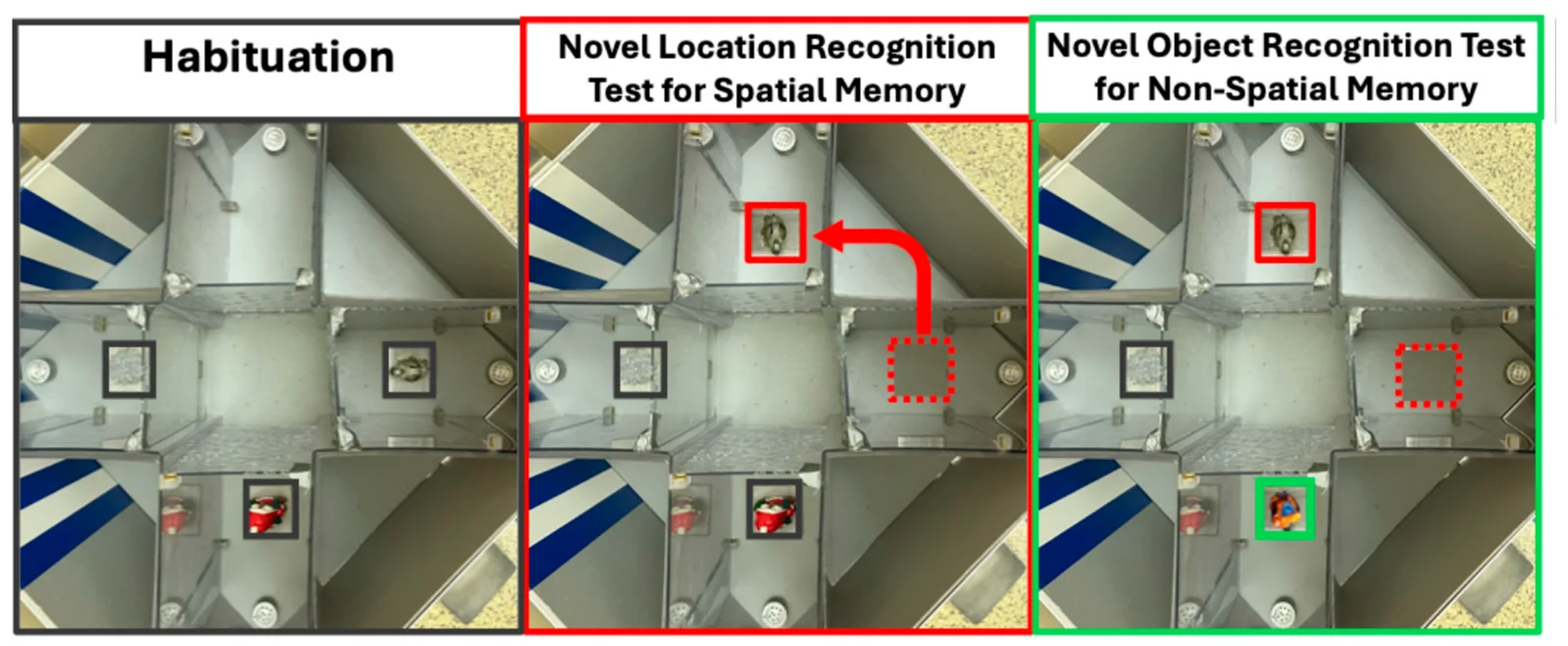
Representative images showing the procedure followed for habituation, Novel Location and Novel Object Recognition test. The color surrounding each image of the maze corresponds with the color of the box around the object and arrow representing the manipulation in that test. Habituation occurred for 4 trials per day over 5 consecutive days. The sixth day begins with two trials of habituation, then introduces the Novel Location Recognition test on the 3rd trial and Novel Object Recognition test on the 4th trial.

The 5-day habituation period established familiarity with the maze and object configuration and provided a baseline against which subsequent responses to novelty could be evaluated. On Day 6, Trials 1 and 2 maintained the familiar configuration and served as baseline/re-habituation trials. During Trial 3, NLR was assessed by relocating one familiar object to the previously empty arm while maintaining object identity. Increased exploration of the relocated object was interpreted as spatial recognition of the changed location. During Trial 4, NOR was assessed by replacing a separate familiar object with a novel object while maintaining its spatial location. Increased exploration of the novel object was interpreted as non-spatial recognition of object identity. Figure 5 presents a visual representation of object set-up on habituation and manipulation trials.

#### 2.9.3. Behavioral Quantification and Statistical Analysis

Total exploration time across all object areas was quantified for each trial. To characterize habituation and determine whether test-day novelty responses were independent of generalized changes in exploratory activity, exploration was quantified as the amount of time spent within the object areas as a percent of total trial duration (% of 3 min per trial). To account for times when the mouse may be sitting on top of the object rather than exploring it, the amount of time the mouse spent with its center body point within a central 1-inch-diameter circle within the object area was subtracted from total object exploration time. Trials 1 + 2 and Trials 3 + 4 were summed for each day (6 min total per comparison) and their group means were compared using paired-samples *t*-tests across Days 1–6.

On Day 6, exploration of the manipulation-specific object areas was compared between Trials 1 + 2 (familiar/baseline exposure) and Trials 3 + 4 (novelty exposure) using paired-samples *t*-tests. These comparisons assessed whether the introduction of a novel location or novel object selectively altered exploration, thus providing measures of spatial and non-spatial recognition memory. Statistical significance was defined as *p* < 0.05, and data are presented as mean ± SEM.

## 3. Results

### 3.1. MRI Results

**Figure 6:**
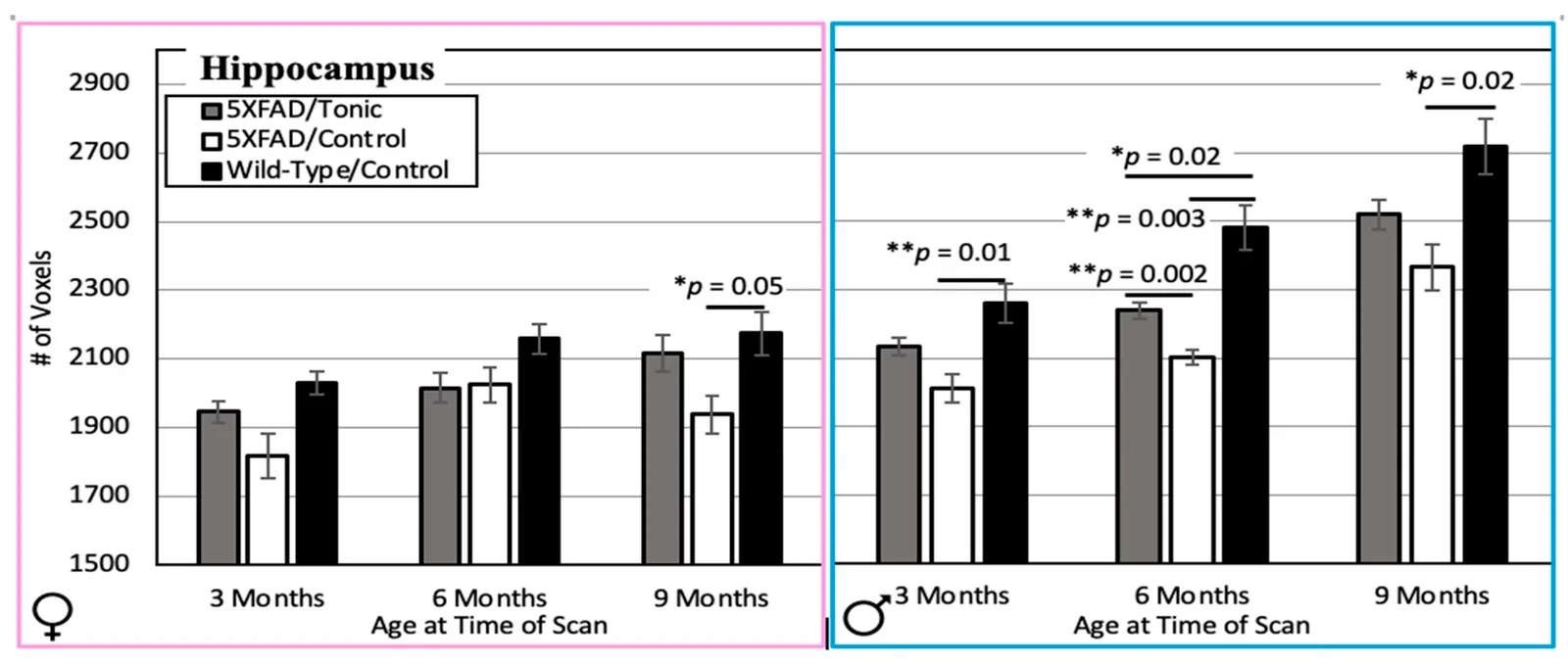
Longitudinal magnetic resonance imaging (MRI) analysis of hippocampal volume in female (pink) and male (blue) wild-type/control, 5XFAD/control, and Tonic-treated 5XFAD mice at 3, 6, and 9 months of age (*n* = 7–8 mice per group). Hippocampal volume was quantified by segmentation and expressed as the total number of voxels. Data are presented as mean ± SEM for male and female mice. Statistical significance was determined using Bonferroni multiple-comparisons tests following the two-way repeated measures analysis of variance (ANOVA). “#” = number, * *p* < 0.05, ** *p* < 0.01.

**Figure 7:**
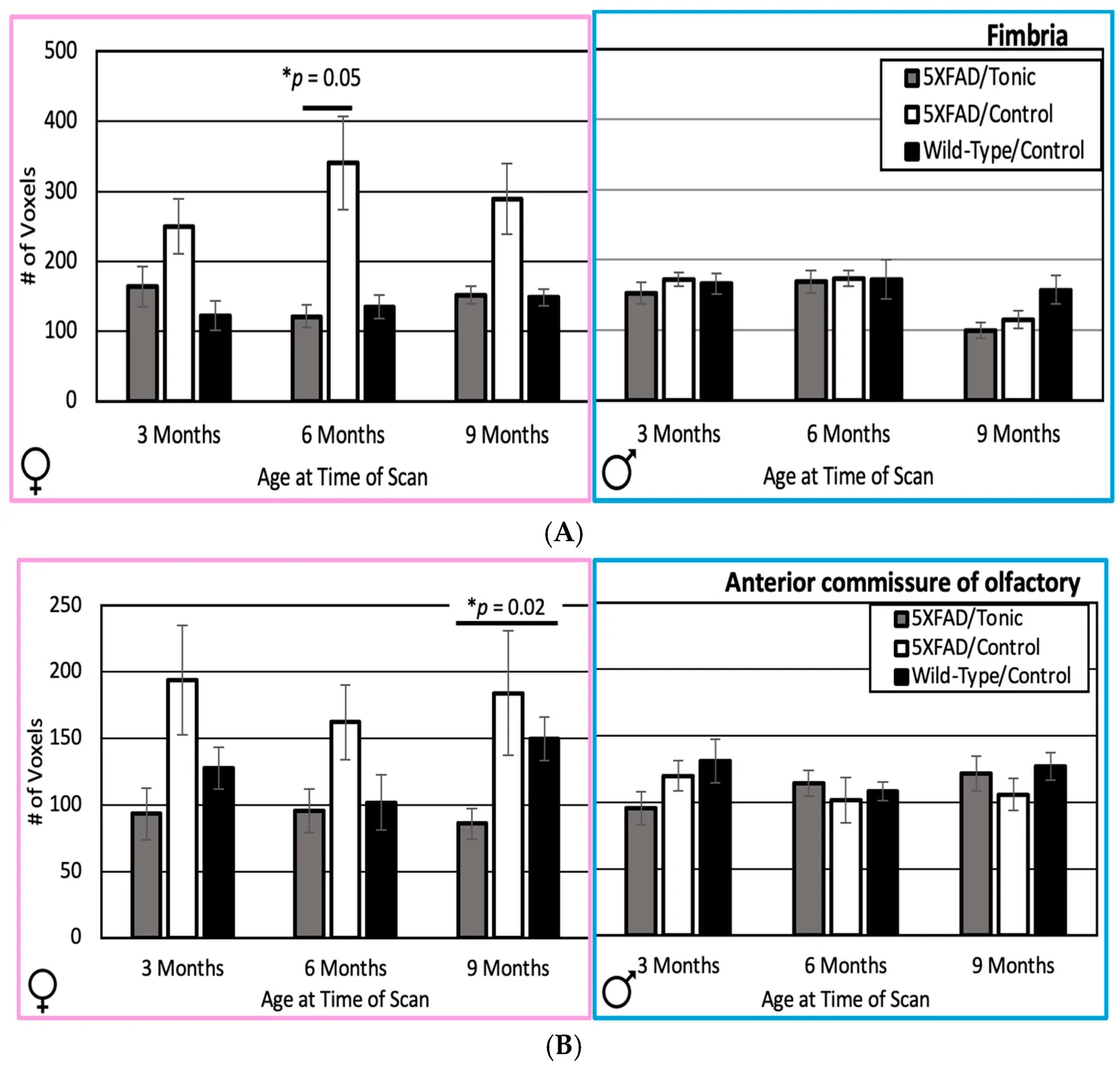

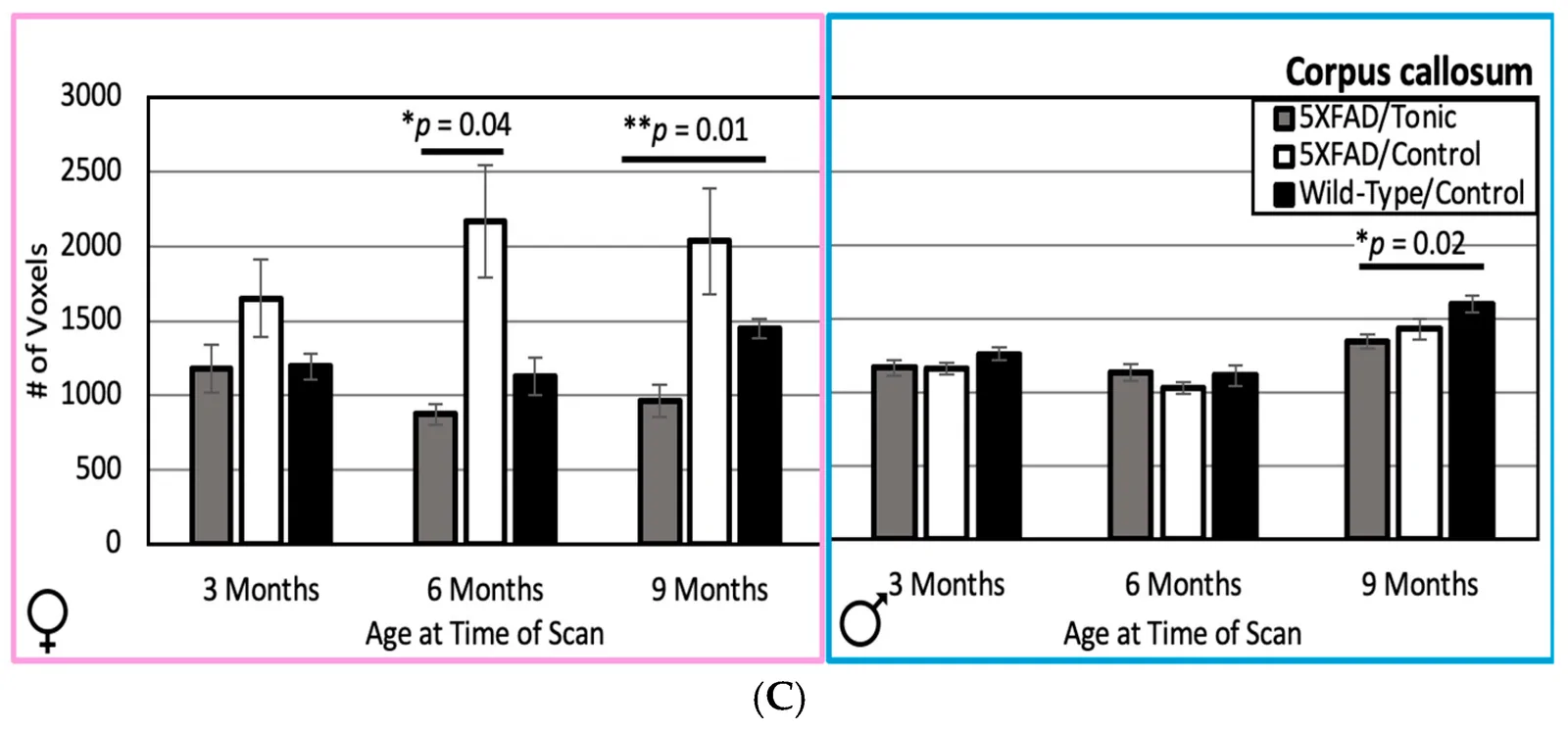
Longitudinal magnetic resonance imaging (MRI) quantification of white matter structures, including (**A**) the fimbria, (**B**) anterior commissure of the olfactory tract, and (**C**) corpus callosum, in female (pink) and male (blue) wild-type/control, 5XFAD/control, and 5XFAD/Tonic-treated mice at 3, 6, and 9 months of age (*n* = 7–8 mice per group). Regional volumes were determined by MRI segmentation and expressed as total voxel counts. Data are presented as mean ± SEM. Statistical significance was determined using Bonferroni multiple-comparisons tests following the two-way repeated measures analysis of variance (ANOVA). “#” = number, * *p* < 0.05, ** *p* < 0.01.

MRI-based volumetric segmentation revealed interesting age- and sex-dependent differences in several brain regions among wild-type/control, 5XFAD/control, and 5XFAD/Tonic groups at 3, 6, and 9 months of age. Figure 6 presents the quantified volumetric assessment of hippocampal volume (# of voxels), and Figure 7A–C presents assessment of volumetric segmentation of white matter structures (fimbria, anterior commissure of olfactory and corpus callosum). Data were analyzed separately by sex using two-way repeated-measures ANOVA, with age as the repeated-measures factor and group as the between-subjects factor. When significant effects were detected, Bonferroni-corrected multiple comparisons were used to identify differences between groups at individual timepoints. The analysis evaluated main effects of time and group, as well as time × group interactions. Supplementary Table S1 details statistical output from two-way repeated measures ANOVAs of the brain regions of interest. Bonferroni-corrected comparisons are reported in Supplementary Table S1.

#### 3.1.1. Hippocampal Volume

In females, hippocampal volume showed significant main effects of time (*F*(1.77, 33.69) = 10.85, *p* = 0.0004) and group (*F*(2,19) = 7.319, *p* = 0.0044), with no significant time × group interaction (*F*(3.55, 33.69) = 1.25, *p* = 0.31). The group effect was large (partial η^2^ = 0.46; *f* = 0.91), as was the time effect (partial η^2^ = 0.36; *f* = 0.76), indicating substantial group-associated and age-related variation in hippocampal volume. The time × group interaction was moderate (partial η^2^ = 0.12; *f* = 0.36), although it was not statistically significant, indicating that the overall longitudinal trajectories did not differ significantly between groups. Post hoc Bonferroni-corrected analysis nevertheless identified a significant difference between wild-type/control and 5XFAD/control mice at 9 months (corrected *p* = 0.045), with 5XFAD/Control mice exhibiting lower hippocampal volume. No significant differences between groups were detected at 3 or 6 months. Thus, although there was a substantial overall group-associated effect on hippocampal volume, the absence of a significant time × group interaction indicates that the longitudinal pattern of change did not significantly differ between groups.

In males, significant main effects of time (*F*(1.50, 29.96) = 82.86, *p* < 0.0001) and group (*F*(2,20) = 17.74, *p* < 0.0001) were observed, with no significant time × group interaction (*F*(3.00, 29.96) = 0.91, *p* = 0.45). Bonferroni-corrected comparisons revealed significantly lower hippocampal volume in 5XFAD/control compared with wild-type/control mice at 3 months (*p* = 0.01), 6 months (*p* = 0.002), and 9 months (*p* = 0.02). At 6 months, 5XFAD/Tonic mice also differed significantly from both 5XFAD/control (*p* = 0.002) and wild-type/control (*p* = 0.03) mice. The group effect was large (partial η^2^ = 0.73; *f* = 1.63), while the time effect was also large (partial η^2^ = 0.81; *f* = 2.04), indicating substantial group-associated and age-related variation. The time × group interaction was moderate (partial η^2^ = 0.08; *f* = 0.30) and did not indicate a significant differential longitudinal trajectory.

#### 3.1.2. Fimbria Volume

Female fimbria volume showed a significant main effect of group (*F*(2,19) = 11.48, *p* = 0.0005), but neither the main effect of time (*F*(1.530, 29.08) = 0.53, *p* = 0.55) nor the time × group interaction (*F*(3.06, 29.08) = 1.68, *p* = 0.19) was significant. The group effect was large (partial η^2^ = 0.64; *f* = 1.35), whereas the time effect was small (partial η^2^ = 0.03; *f* = 0.17). The time × group interaction was large by η^2^ criteria (partial η^2^ = 0.15; *f* = 0.42), although it was not statistically significant. Therefore, fimbria volume was strongly associated with experimental group, while relatively little variation was attributable to age, with a comparatively substantial but statistically unsupported indication that longitudinal changes may have varied by group. Bonferroni-corrected comparisons identified a significant difference between 5XFAD/Tonic and 5XFAD/Control mice at 6 months (*p* = 0.05), with lower fimbria volume in Tonic-treated mice. No other group comparisons at individual timepoints reached significance.

In males, fimbria volume demonstrated a significant main effect of time (*F*(1.33, 26.53) = 40.18, *p* < 0.0001), while neither the main effect of group (*F*(2,20) = 0.82, *p* = 0.46) nor the time × group interaction (*F*(2.65, 26.53) = 0.42, *p* = 0.72) was significant. The time effect was large (partial η^2^ = 0.67; *f* = 1.42), whereas the group effect was small (partial η^2^ = 0.03; *f* = 0.18), and the interaction was small (partial η^2^ = 0.04; *f* = 0.21), with neither reaching statistical significance. Therefore, in males, age was the predominant source of variation in fimbria volume, with comparatively little evidence that experimental group or treatment altered either overall volume or its longitudinal trajectory.

#### 3.1.3. Anterior Commissure of the Olfactory Volume

In females, anterior commissure volume showed a significant main effect of group (*F*(2,19) = 10.40, *p* = 0.0009), with no significant effect of time (*F*(1.79, 34.04) = 0.50, *p* = 0.59) or time × group interaction (*F*(3.58, 34.04) = 0.33, *p* = 0.84). The group effect was large (partial η^2^ = 0.29; *f* = 0.64), while the time effect (partial η^2^ = 0.03; *f* = 0.16) and interaction (partial η^2^ = 0.03; *f* = 0.19) were small. The effect sizes indicate that experimental group accounted for a substantial proportion of variation in female anterior commissure volume, whereas age- and group-dependent longitudinal changes contributed relatively little. Bonferroni-corrected comparisons identified a significant difference between 5XFAD/Tonic and wild-type/control mice at 9 months (*p* = 0.02). No other timepoint-specific group comparisons were significant.

No significant main effects of time (*F*(1.82, 36.48) = 0.59, *p* = 0.54), group (*F*(2,20) = 0.78, *p* = 0.47), or time × group interaction (*F*(3.65, 36.48) = 1.23, *p* = 0.32) were detected in males. The group effect (partial η^2^ = 0.05; *f* = 0.23), time effect (partial η^2^ = 0.03; *f* = 0.17), and interaction (partial η^2^ = 0.11; *f* = 0.35) were small to moderate, with no statistically significant effects. Thus, male anterior commissure volume showed relatively limited variation attributable to group, age, or their interaction, consistent with broadly comparable volumes across groups over time.

#### 3.1.4. Corpus Callosum Volume

In females, corpus callosum volume demonstrated significant main effects of group (*F*(2,19) = 7.32, *p* = 0.004) and a significant time × group interaction (*F*(3.22, 30.58) = 3.41, *p* = 0.03), whereas the main effect of time was not significant (*F*(1.61, 30.58) = 0.97, *p* = 0.37). The group effect was large (partial η^2^ = 0.70; *f* = 1.51), as was the time × group interaction (partial η^2^ = 0.26; *f* = 0.60), indicating substantial group-associated variation and a meaningful group-dependent component of longitudinal variation. The time effect was small (partial η^2^ = 0.05; *f* = 0.23), indicating that age alone contributed relatively little to variation. Bonferroni-corrected comparisons revealed significantly higher corpus callosum volume in 5XFAD/control compared with 5XFAD/Tonic mice at 6 months (*p* = 0.04). At 9 months, 5XFAD/Tonic mice also exhibited significantly lower corpus callosum volume than wild-type/control mice (*p* = 0.01). Therefore, the result suggests high variation in female corpus callosum volume across the longitudinal testing period and also between group conditions.

In males, corpus callosum volume showed a significant main effect of time (*F*(1.52, 30.32) = 53.09, *p* < 0.0001), but neither the main effect of group (*F*(2,20) = 2.58, *p* = 0.10) nor the time × group interaction (*F*(3.03, 30.32) = 2.40, *p* = 0.09) was significant. The time effect was large (partial η^2^ = 0.73; *f* = 1.63), whereas the group effect was large by η^2^ criteria (partial η^2^ = 0.24; *f* = 0.55), and the interaction was large by η^2^ criteria (partial η^2^ = 0.19; *f* = 0.49), although neither the group nor interaction effect was statistically significant. The effect sizes indicate that age was the predominant source of variation in male corpus callosum volume, while the group and interaction effects suggest some potential group-associated and trajectory-related variation that was not sufficiently supported statistically. Bonferroni-corrected comparisons identified a significant difference between 5XFAD/Tonic and wild-type/control mice at 9 months (*p* = 0.021); however, because the overall group effect was not significant, this isolated pairwise difference should be interpreted cautiously.

### 3.2. Nissl Stain Results

#### Corpus Callosum Nissl Body Density

**Figure 8:**
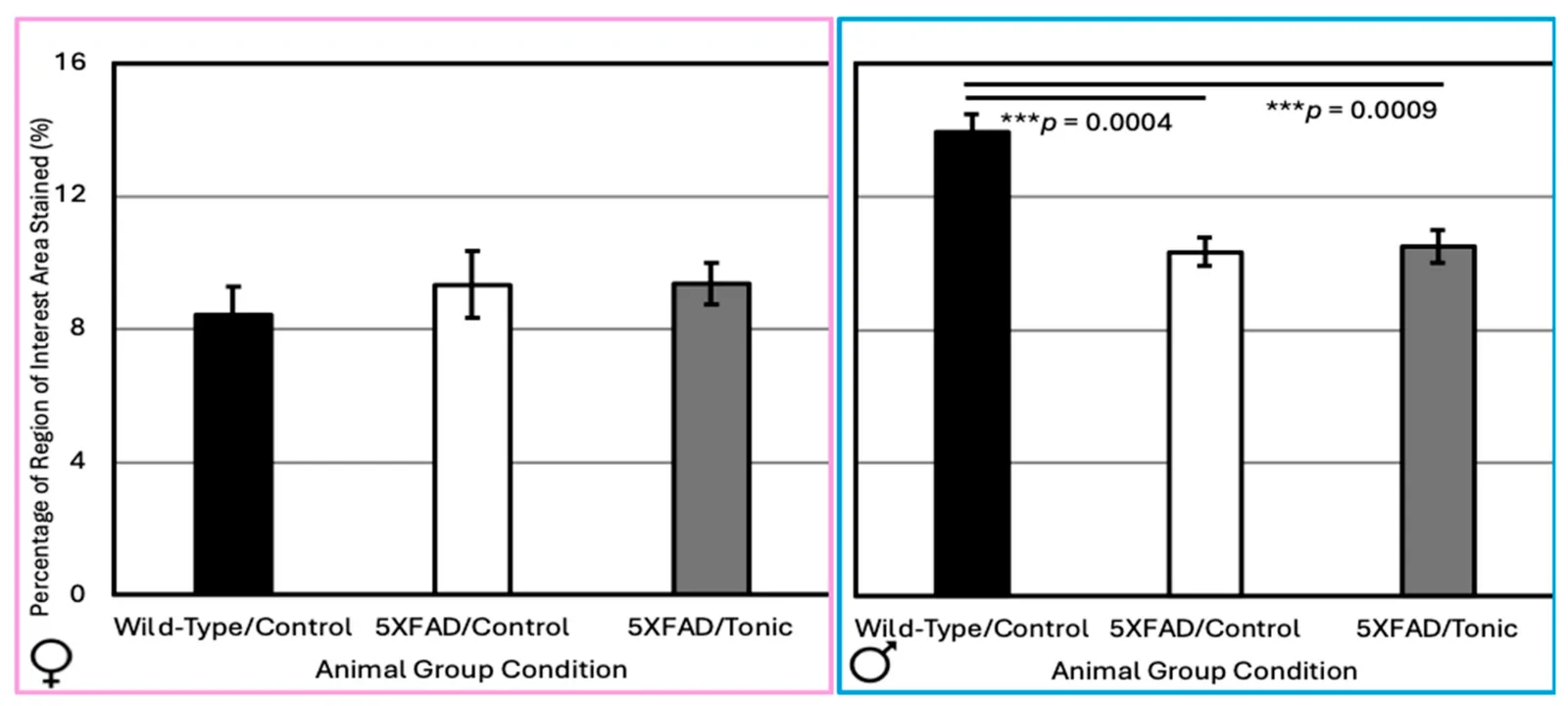
Quantitative analysis of Nissl-positive staining within the corpus callosum of male (blue) and female (pink) wild-type/control, 5XFAD/control, and 5XFAD/Tonic-treated mice (*n* = 3 mice per group, 3 fields per mouse). The percentage of the region of interest occupied by Nissl staining was quantified using ImageJ. Data are presented as mean ± SEM. Statistical significance was determined using one-way analysis of variance (ANOVA) followed by Bonferroni-corrected Welch’s *t*-tests. *** *p* < 0.001.

Figure 8 represents corpus callosum Nissl stain results for cell density as % of corpus callosum stained. In female mice, there was no significant effect of treatment on corpus callosum Nissl body area percentage (one-way ANOVA, *F*(2,21) = 0.38, *p* = 0.69, η^2^ = 0.04). Mean Nissl body area was similar among 5XFAD/control mice (M = 14.01 ± 1.20%), Tonic-treated 5XFAD mice (M = 12.47 ± 1.92%), and wild-type/controls (M = 10.69 ± 2.33%). The treatment effect was small and nonsignificant, indicating little group-associated variance in Nissl body area percentage.

In male mice, treatment significantly affected corpus callosum Nissl body area percentage (one-way ANOVA, *F*(2,20) = 17.62, *p* < 0.001, η^2^ = 0.64). Wild-type/control mice exhibited a greater Nissl body area (M = 9.02 ± 1.22%) than both 5XFAD control mice (M = 7.82 ± 1.44%) and Tonic-treated 5XFAD mice (M = 8.15 ± 0.61%). The treatment effect was large, indicating substantial group-associated variance. The pairwise Cohen’s *d* values further suggest a very large difference between untreated 5XFAD and wild-type/control mice (*d* = −2.511) and between Tonic-treated 5XFAD and WT/control mice (*d* = −2.322), whereas the difference between untreated and Tonic-treated 5XFAD mice was negligible (*d* = −0.132). One-way ANOVA results are reported in Supplementary Table S2. Pairwise Bonferroni-corrected comparisons are reported in Supplementary Table S2. Qualitative representative images from male and female brains at 20× and 40× magnification are provided in Supplementary Figure S1.

### 3.3. Immunofluorescence Analysis Results

Complete one-way ANOVA and pairwise comparisons are summarized in Supplementary Table S3, respectively.

#### 3.3.1. Aβ

**Figure 9:**
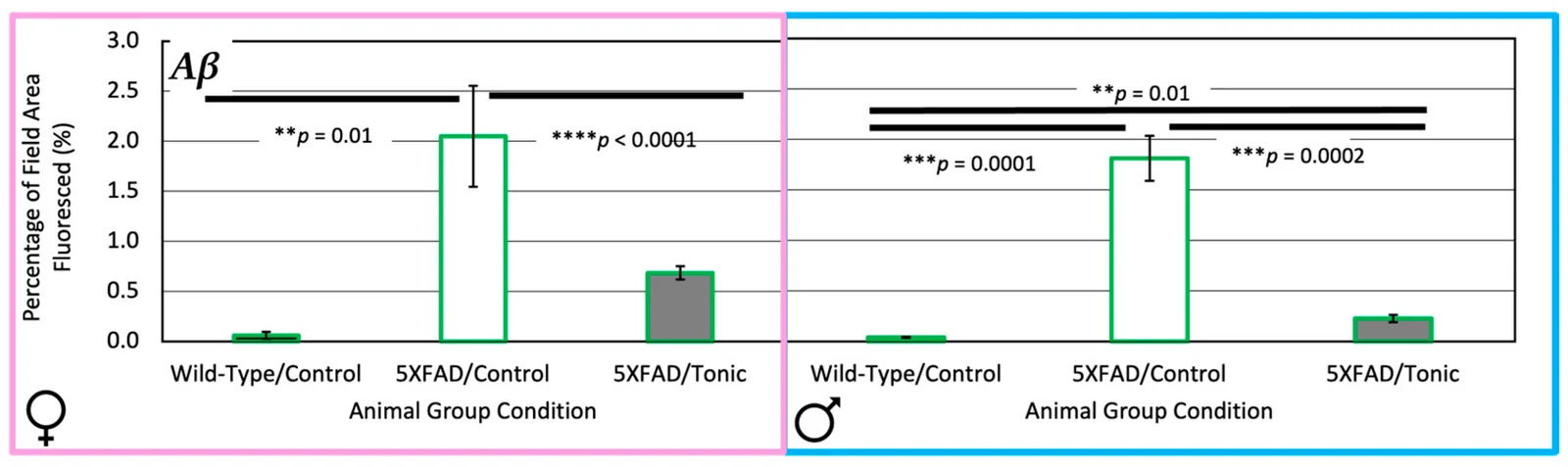
Quantitative analysis of amyloid-β (Aβ) immunoreactivity in the dentate gyrus and CA1 (Cornu Ammonis area 1) region of the hippocampus of male (blue) and female (pink) wild-type/control, 5XFAD/control, and 5XFAD/Tonic-treated mice. Aβ burden was quantified as the percentage of imaging field area (20× magnification) that met or surpassed threshold fluorescence using ImageJ (*n* = 3 mice per group, 3 fields per mouse). Data are presented as mean ± SEM. Statistical significance was determined using one-way analysis of variance (ANOVA) followed by Bonferroni-corrected Welch’s *t*-tests. ** *p* < 0.01, *** *p* < 0.001, **** *p* < 0.00001.

As shown in Figure 9, Aβ immunoreactivity was significantly altered among experimental groups in both male and female mice (males: one-way ANOVA, *F*(2,20) = 42.24, *p* < 0.0001, η^2^ = 0.81; females: *F*(2,24) = 11.95, *p* = 0.0003, η^2^ = 0.50). Male 5XFAD/control mice exhibited approximately a 45-fold increase in Aβ immunoreactivity compared with wild-type/controls (M = 1.81 ± 0.22 vs. 0.04 ± 0.01%), while Tonic treatment reduced amyloid burden to M = 0.21 ± 0.04%. Similarly, untreated female 5XFAD/control mice demonstrated markedly greater Aβ accumulation than wild-type/control animals (M = 2.05 ± 0.50 vs. 0.06 ± 0.03%), with Tonic treatment reducing amyloid deposition to M = 0.69 ± 0.07%. The male group effect was very large, while the female effect was large. In males, the untreated 5XFAD versus wild-type comparison showed a very large difference (*d* = −3.384), and the untreated versus Tonic comparison was also very large (*d* = 3.241). Tonic-treated males remained significantly different from wild-type/controls, with a very large effect (*d* = −1.945). In females, wild-type versus 5XFAD/control also showed a very large difference (*d* = −1.856), while wild-type versus Tonic was particularly large (*d* = −3.902). The untreated versus Tonic comparison was large (*d* = 1.266) but was not statistically significant after correction. The effect sizes therefore indicate that experimental group accounted for substantial variation in amyloid burden in both sexes, with particularly strong separation among groups in males and a marked difference between untreated 5XFAD and Tonic-treated animals, supporting a substantial reduction in Aβ immunoreactivity in males. Bonferroni-adjusted post hoc comparisons demonstrated significant increases in Aβ expression in 5XFAD/control mice compared with wild-type/controls in both sexes. Tonic treatment significantly reduced amyloid burden in males and produced a substantial, although not statistically significant following correction, reduction in females. Supplementary Figure S2A provides qualitative representative images of Aβ immunoreactivity in both males and females.

#### 3.3.2. APP

**Figure 10:**
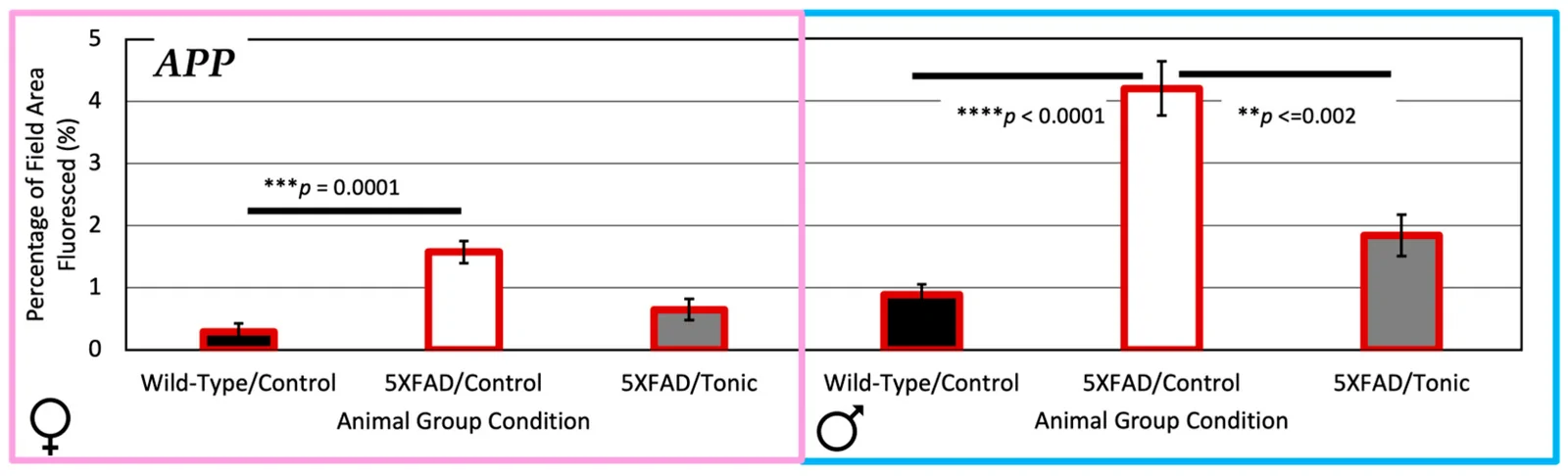
Quantitative analysis of amyloid precursor protein (APP) expression in the dentate gyrus and CA1 (Cornu Ammonis area 1) region of the hippocampus of male (blue) and female (pink) wild-type/control, 5XFAD/control, and 5XFAD/Tonic-treated mice. APP immunoreactivity was quantified as the percentage of imaging field area (20× magnification) that met or surpassed threshold fluorescence using ImageJ (*n* = 3 mice per group, 3 fields per mouse). Data are presented as mean ± SEM. Statistical significance was determined using one-way analysis of variance (ANOVA) followed by Bonferroni-corrected Welch’s *t*-tests. ** *p* < 0.01, *** *p* < 0.001, **** *p* < 0.0001.

APP immunoreactivity differed significantly among experimental groups in both male (*F*(2,20) = 21.56, *p* < 0.0001, η^2^ = 0.68) and female mice (*F*(2,24) = 16.52, *p* < 0.0001, η^2^ = 0.58), as shown in Figure 10. 5XFAD/control mice exhibited markedly elevated APP expression compared with wild-type/controls in both males (M = 4.20 ± 0.44 vs. 0.85 ± 0.20%) and females (M = 1.58 ± 0.18 vs. 0.29 ± 0.13%). Group effects were large in both males and females. In males, 5XFAD/control versus wild-type/control showed a very large difference (*d* = −3.143), while the untreated versus Tonic comparison was also very large (*d* = 2.091). The Tonic versus wild-type comparison was large (*d* = −1.097) but nonsignificant. In females, wild-type/control versus 5XFAD/control showed a very large difference (*d* = −2.711), and untreated versus Tonic showed a very large difference (*d* = 1.754), whereas Tonic versus wild-type showed a large but nonsignificant standardized difference (*d* = −0.775). The resulting effect sizes indicate that experimental group accounted for substantial variation in APP immunoreactivity in both sexes, with the large pairwise separation between untreated 5XFAD and Tonic-treated mice indicating a pronounced treatment-associated reduction that brought APP levels closer to those of wild-type/controls. Tonic treatment reduced APP immunoreactivity to M = 1.75 ± 0.35% in males and M = 0.65 ± 0.17% in females, restoring expression to levels that were not significantly different from wild-type/control animals. Supplementary Figure S2B provides qualitative representative images of APP immunoreactivity.

#### 3.3.3. Iba-1

**Figure 11:**
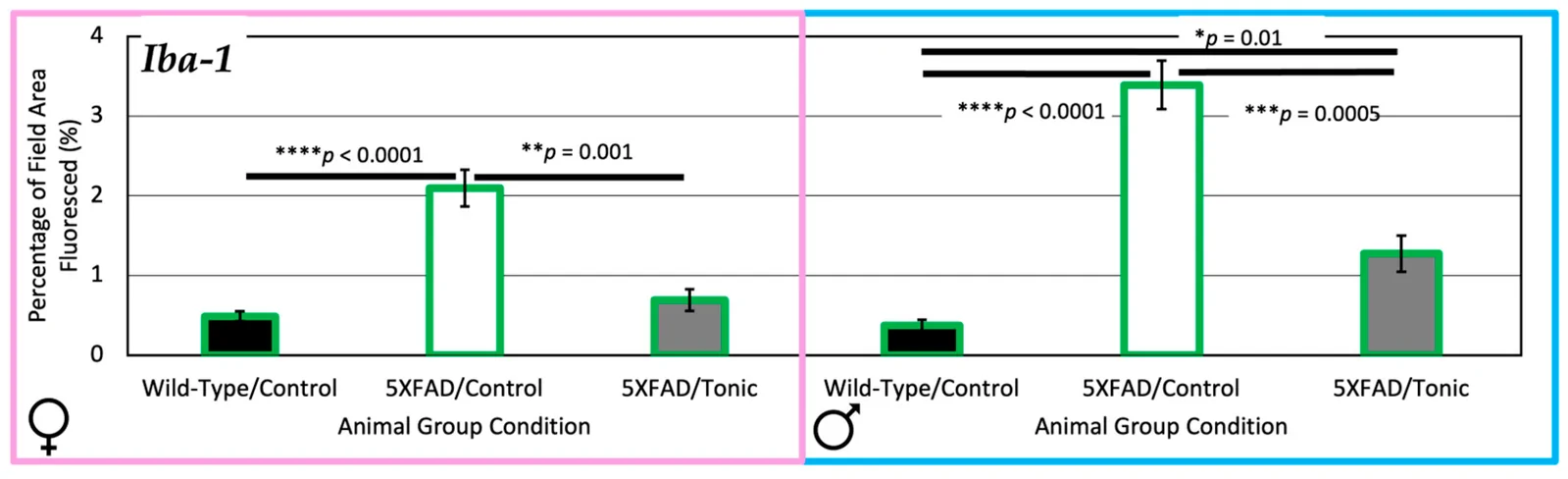
Quantitative analysis of Ionized calcium-binding adapter molecule 1 (Iba-1) expression in the dentate gyrus and CA1 (Cornu Ammonis area 1) region of the hippocampus of male (blue) and female (pink) wild-type/control, 5XFAD/control, and 5XFAD/Tonic-treated mice. Iba-1 immunoreactivity was quantified as the percentage of imaging field area (20× magnification) that met or surpassed threshold fluorescence using ImageJ as a measure of microglial activation (*n* = 3 mice per group, 3 fields per mouse). Data are presented as mean ± SEM. Statistical significance was determined using one-way analysis of variance (ANOVA) followed by Bonferroni-corrected Welch’s *t*-tests. * *p* < 0.05, ** *p* < 0.01, *** *p* < 0.001, **** *p* < 0.0001.

Iba-1 immunoreactivity, indicative of microglial activation, differed significantly among experimental groups in both males (*F*(2,23) = 45.33, *p* < 0.0001, η^2^ = 0.80) and females (*F*(2,19) = 22.50, *p* < 0.0001, η^2^ = 0.70), seen in Figure 11. The group effect (η^2^) was very large in males and large in females. As for magnitude of difference for each comparison, male wild-type/control versus 5XFAD/control (*d* = −4.632), wild-type/control versus 5XFAAD/Tonic (*d* = −1.786), and untreated versus Tonic (*d* = 2.606) all represented very large standardized differences. In females, wild-type versus untreated 5XFAD (*d* = −2.982) and untreated versus Tonic (*d* = 2.287) were very large, whereas Tonic versus wild-type was moderate (*d* = −0.552) and nonsignificant. The effect sizes indicate that experimental group accounted for substantial variation in microglial immunoreactivity, particularly in males, with large separation between untreated and Tonic-treated 5XFAD mice consistent with a marked treatment-associated reduction in Iba-1 expression. Untreated male 5XFAD/control mice demonstrated a nearly ninefold increase in Iba-1 expression compared with wild-type/controls (M = 3.35 ± 0.32 vs. 0.37 ± 0.07%), while Tonic treatment reduced expression to M = 1.27 ± 0.23%. Female mice displayed a similar pattern, with 5XFAD/control mice exhibiting significantly elevated Iba-1 expression (M = 2.09 ± 0.23%) relative to wild-type/controls (M = 0.49 ± 0.08%) whereas Tonic-treated 5XFAD mice exhibited levels comparable with wild-type/control animals (M = 0.69 ± 0.19%). Qualitative representative images are provided in Supplementary Figure S2C.

#### 3.3.4. LC3B

**Figure 12:**
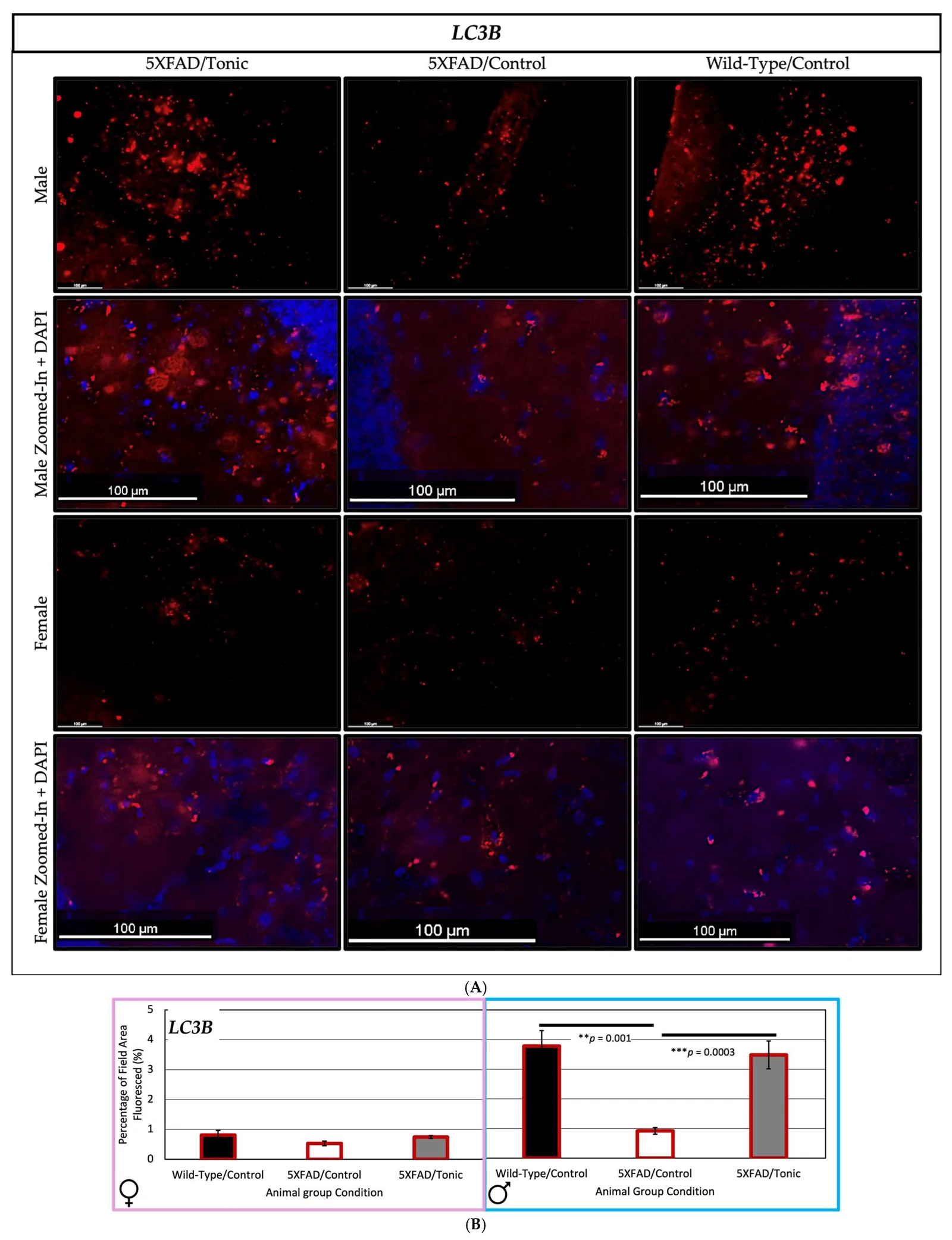
(**A**) Representative immunofluorescence images and zoomed-in photos with DAPI for puncta assessment and (**B**) quantitative analysis of microtubule-associated protein 1 light chain 3B (LC3B) expression in the dentate gyrus and CA1 (Cornu Ammonis area 1) region of the hippocampus of male (blue) and female (pink) wild-type/control, 5XFAD/control, and 5XFAD/Tonic-treated mice. Representative images were taken at 20× magnification and scale bar = 100 μm. LC3B immunoreactivity was quantified as the percentage of imaging field area that met or surpassed threshold fluorescence using ImageJ (*n* = 3 mice per group, 3 fields per mouse). Data are presented as mean ± SEM. Statistical significance was determined using one-way analysis of variance (ANOVA) followed by Bonferroni-corrected Welch’s *t*-tests. ** *p* < 0.01, *** *p* < 0.001.

As shown in Figure 12A,B, LC3B immunoreactivity differed significantly among male groups (*F*(2,23) = 14.92, *p* = 0.00007, η^2^ = 0.57), but not female groups (*F*(2,19) = 1.19, *p* = 0.33, η^2^ = 0.11). The male group effect was large. Wild-type versus untreated 5XFAD showed a very large difference (*d* = 2.446), while untreated versus Tonic also showed a very large difference (*d* = −2.030). The Tonic versus wild-type comparison was small (*d* = 0.490) and nonsignificant, indicating that the treated group was relatively close to wild-type levels. The female group effect was small to moderate and nonsignificant, suggesting the magnitude of expression within each group was negligible and relatively similar amongst all subjects. Male 5XFAD/control mice exhibited substantially reduced LC3B expression compared with wild-type/controls (M = 0.92 ± 0.12 vs. 3.78 ± 0.52%), whereas Tonic treatment restored LC3B immunoreactivity to levels comparable with wild-type/control mice (M = 3.48 ± 0.47%). In females, LC3B expression remained relatively consistent across groups, ranging from M = 0.53 ± 0.08 to 0.79 ± 0.08%, with no significant differences detected.

Qualitatively looking at the zoomed-in representative images of LC3B puncta with DAPI (Figure 12A), 5XFAD/control males show relatively sparse LC3B signal with fewer obvious punctate structures, whereas 5XFAD/Tonic males show prominent LC3B signal/puncta than untreated 5XFAD, similar to male WT/control. Now despite female overall immune-response being lesser than males, the female 5XFAD/Tonic and wild-type/control mice appear to have increased LC3B immunoreactivity and more prominent punctate signal compared with untreated female 5XFAD/control, which show very sparse fluorescent signals. In all, Tonic treatment appeared to alter the spatial organization of LC3B immunoreactivity by producing a more distinct punctate pattern with numerous LC3B-positive structures and localized puncta clusters, whereas 5XFAD/control mice had more diffuse LC3B signal.

#### 3.3.5. IL-1β

**Figure 13:**
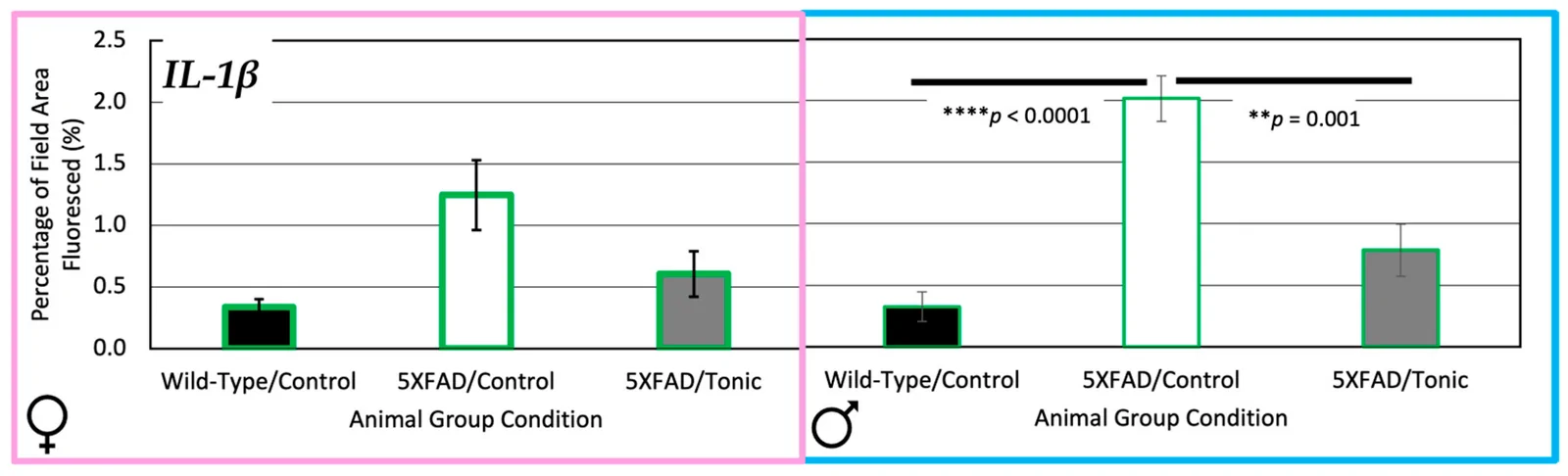
Quantitative analysis of interleukin-1β (IL-1β) expression in the dentate gyrus and CA1 (Cornu Ammonis area 1) region of the hippocampus of male (blue) and female (pink) wild-type/control, 5XFAD/control, and 5XFAD/Tonic-treated mice. IL-1β immunoreactivity was quantified as the percentage of imaging field area (20× magnification) that met or surpassed threshold fluorescence using ImageJ (*n* = 3 mice per group, 3 fields per mouse). Data are presented as mean ± SEM. Statistical significance was determined using one-way analysis of variance (ANOVA) followed by Bonferroni-corrected Welch’s *t*-tests. ** *p* < 0.01, **** *p* < 0.0001.

IL-1β immunoreactivity differed significantly among male experimental groups (*F*(2,24) = 24.63, *p* < 0.0001, η^2^ = 0.67), represented in Figure 13. Wild-type versus 5XFAD/controls showed a very large difference in immunoreactivity (*d* = −3.616), while untreated versus Tonic also showed a very large difference (*d* = 2.071). The 5XFAD/Tonic versus wild-type/control difference was large (*d* = −0.890) but nonsignificant. Therefore, the effect sizes indicate substantial group-associated variation in IL-1β immunoreactivity in males, with a pronounced separation between untreated and Tonic-treated 5XFAD mice. Untreated male 5XFAD/control mice demonstrated elevated IL-1β expression (2.02 ± 0.19%) relative to wild-type/controls (M = 0.33 ± 0.12%), while Tonic treatment reduced expression to M = 0.79 ± 0.21%. Female mice also exhibited a significant overall group effect (*F*(2,20) = 4.15, *p* = 0.03, η^2^ = 0.29); however, Bonferroni-adjusted pairwise comparisons were not statistically significant despite a trend toward elevated IL-1β expression in 5XFAD/Control mice (M = 1.28 ± 0.38%) compared with wild-type/controls (M = 0.32 ± 0.07%). The pairwise effects were large for wild-type versus untreated 5XFAD/control (*d* = −1.392), Tonic versus wild-type (*d* = −0.813), and untreated versus Tonic (*d* = 0.776), despite the absence of statistical significance. Qualitative immunofluorescence representative images are provided in Supplementary Figure S2D.

#### 3.3.6. Beclin-1

**Figure 14:**
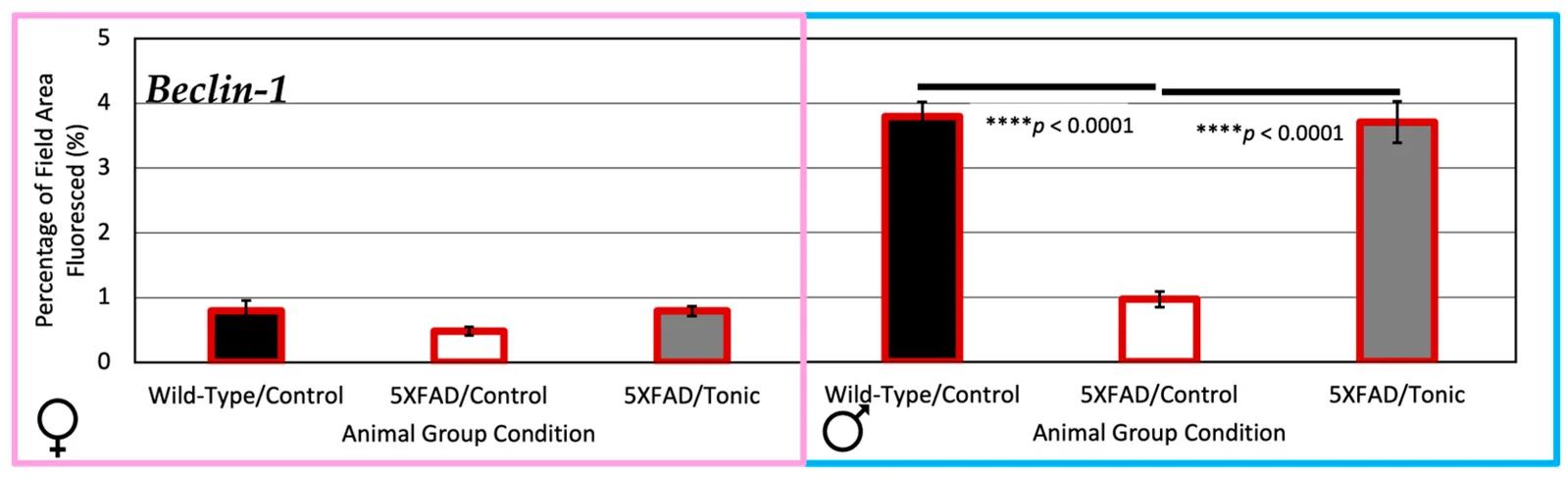
Quantitative analysis of Beclin-1 expression in the hippocampus of male (blue) and female (pink) wild-type/control, 5XFAD/control, and 5XFAD/Tonic-treated mice. Beclin-1 immunoreactivity was quantified as the percentage of imaging field area (20× magnification) that met or surpassed threshold fluorescence using ImageJ (*n* = 3 mice per group, 3 fields per mouse). Data are presented as mean ± SEM. Statistical significance was determined using one-way analysis of variance (ANOVA) followed by Bonferroni-corrected Welch’s *t*-tests. **** *p* < 0.0001.

Beclin-1 immunoreactivity differed significantly among male groups (*F*(2,24) = 46.05, *p* < 0.0001), and the group effect was large (η^2^ = 0.79); however, no significant differences were detected among female groups (*F*(2,20) = 1.80, *p* = 0.19, η^2^ = 0.15). See Figure 14 for quantification of Beclin-1 immunoreactivity. Male wild-type versus untreated 5XFAD showed an extremely large standardized difference (*d* = 5.173), and untreated versus Tonic also showed a very large difference (*d* = −3.783). In contrast, Tonic versus wild-type showed a negligible difference (*d* = 0.105), consistent with the treated group approaching wild-type/control levels. The effect sizes indicate that group condition accounted for substantial variation in Beclin-1 immunoreactivity in males, with Tonic-treated mice closely resembling wild-type/controls and showing marked separation from untreated 5XFAD mice. On the other hand, the female group effect was large by η^2^ criteria, although it was nonsignificant. Male 5XFAD/control mice exhibited markedly reduced Beclin-1 expression compared with wild-type/controls (M = 0.97 ± 0.36 vs. 3.79 ± 0.23%). Tonic treatment restored Beclin-1 immunoreactivity (M = 3.71 ± 0.32%) to levels comparable with wild-type/control mice. Female Beclin-1 expression remained similar among groups, ranging from M = 0.50 ± 0.07 to 0.81 ± 0.10%. Supplementary Figure S2E displays qualitative representative images for Beclin-1 immunoreactivity.

#### 3.3.7. Hippocampal α-GFAP

**Figure 15:**
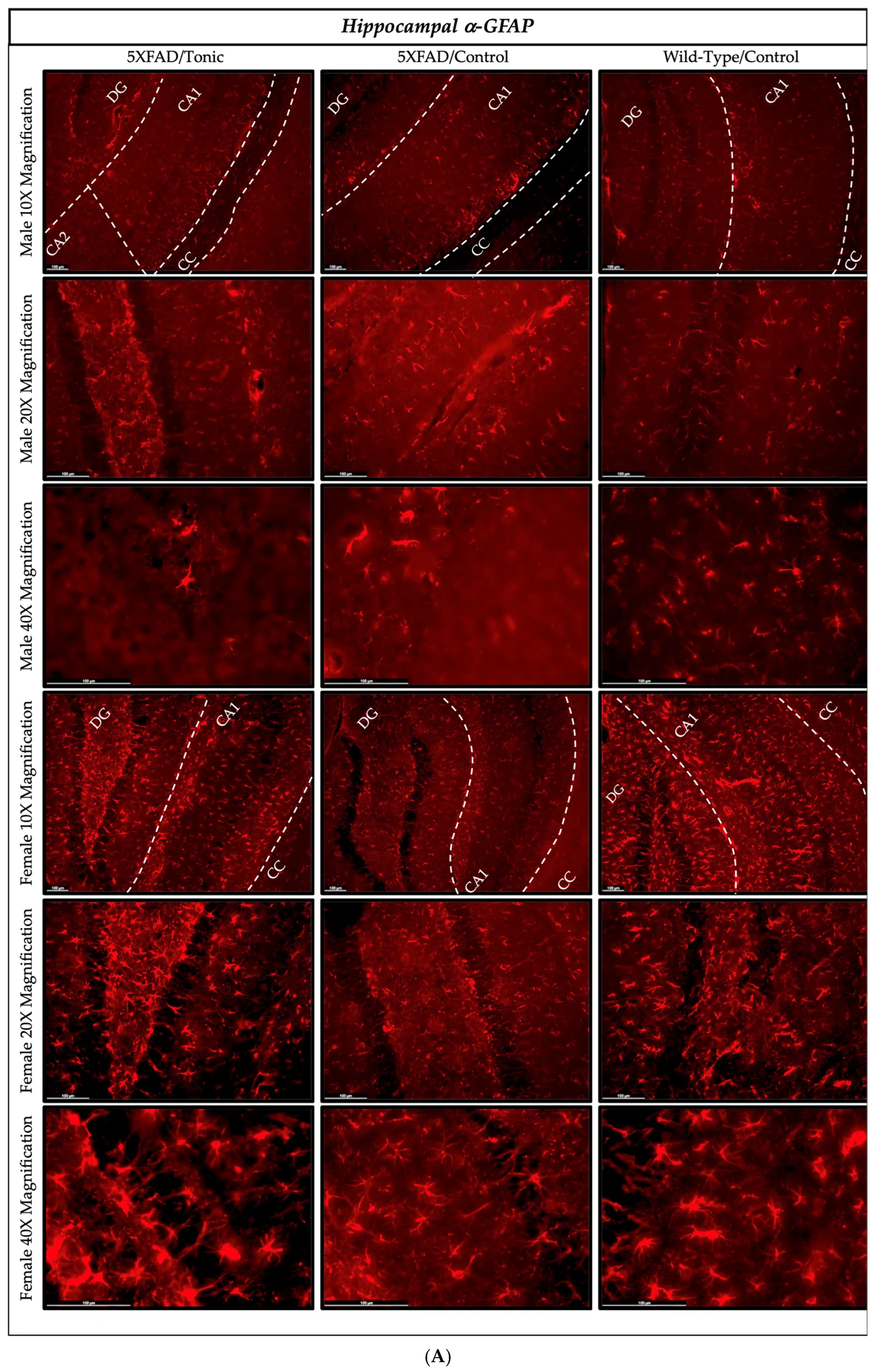

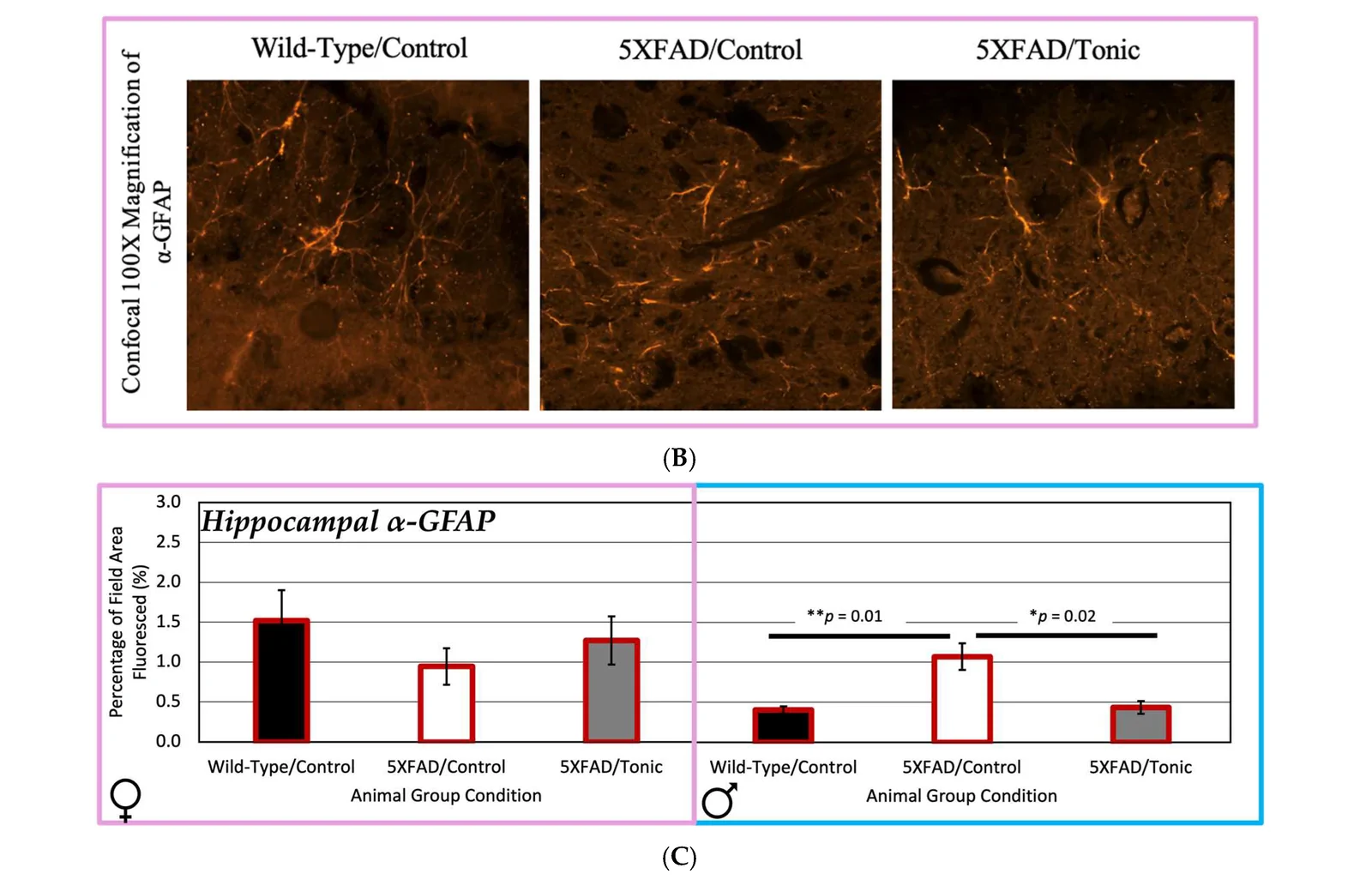
(**A**) Representative immunofluorescence images (10× with anatomical dashed lines and labels of brain region in frame, 20×, and 40× magnification; Scale bar = 100 μm), (**B**) representative confocal microscopy images of the morphological characteristics of female hippocampal astrocytes at 100× magnification, and (**C**) quantitative analysis of α-glial fibrillary acidic protein (α-GFAP) expression in the hippocampus of male (blue) and female (pink) wild-type/control, 5XFAD/control, and 5XFAD/Tonic-treated mice. α-GFAP immunoreactivity was quantified as the percentage of imaging field area that met or surpassed threshold fluorescence using ImageJ (*n* = 3 mice per group, 3 fields per mouse). Data are presented as mean ± SEM. Statistical significance was determined using one-way analysis of variance (ANOVA) followed by Bonferroni-corrected Welch’s *t*-tests. DG = Dentate gyrus, “CC” = Corpus callosum, “CA1” = Cornu Ammonis area 1, “CA2” = Cornu Ammonis area 2. * *p* < 0.05, ** *p* < 0.01.

Hippocampal α-GFAP immunoreactivity differed significantly among male groups (*F*(2,24) = 11.68, *p* = 0.0003, η^2^ = 0.49), but not among female groups (*F*(2,24) = 0.87, *p* = 0.43, η^2^ = 0.07). Wild-type/control versus untreated 5XFAD/control showed a very large difference (*d* = −1.816), and the untreated versus Tonic comparison was very large (*d* = 1.617), whereas 5XFAD/Tonic versus wild-type showed a negligible difference (*d* = −0.158). In females, the group effect was small, although pairwise effects were large: wild-type versus untreated 5XFAD (*d* = 2.186), Tonic versus wild-type (*d* = 0.204), and untreated versus Tonic (*d* = −1.905). The latter pairwise comparisons should be interpreted in conjunction with their statistical significance rather than as evidence of a treatment effect based on magnitude alone. In other words, the effect sizes indicate substantial group-associated variation in hippocampal α-GFAP immunoreactivity in males, with Tonic-treated mice closely resembling wild-type/controls, whereas the comparatively small overall female effect indicates limited group-associated variation despite some large pairwise differences. Male 5XFAD/control mice demonstrated elevated astrocytic GFAP expression (M = 1.07 ± 0.17%) compared with wild-type/controls (M = 0.40 ± 0.05%). Tonic treatment reduced hippocampal α-GFAP expression to levels comparable with wild-type/control mice (M = 0.43 ± 0.08%). In females, hippocampal α-GFAP expression remained relatively consistent among groups (M = 0.94 ± 0.23%). Data for hippocampal α-GFAP expression is shown in Figure 15A–C.

Qualitatively, the 40× magnification images (Figure 15A), morphological characteristics of male and female 5XFAD/control GFAP expression resemble reactive-state astrocytes, since they show thicker, less complex processes, hypertrophic cell bodies, and clustered fluorescent distribution. In contrast, male and female 5XFAD/Tonic do show some slight clustered distribution, but their overall complex branching and thin processes more closely resemble wild-type/control. The images acquired by confocal microscopy (Figure 15B) show female 5XFAD/control mice have more fragmented astrocytes and less immune-expression, consistent with quantitative assessment and suggestive of atrophy or stress.

#### 3.3.8. Corpus Callosum α-GFAP

**Figure 16:**
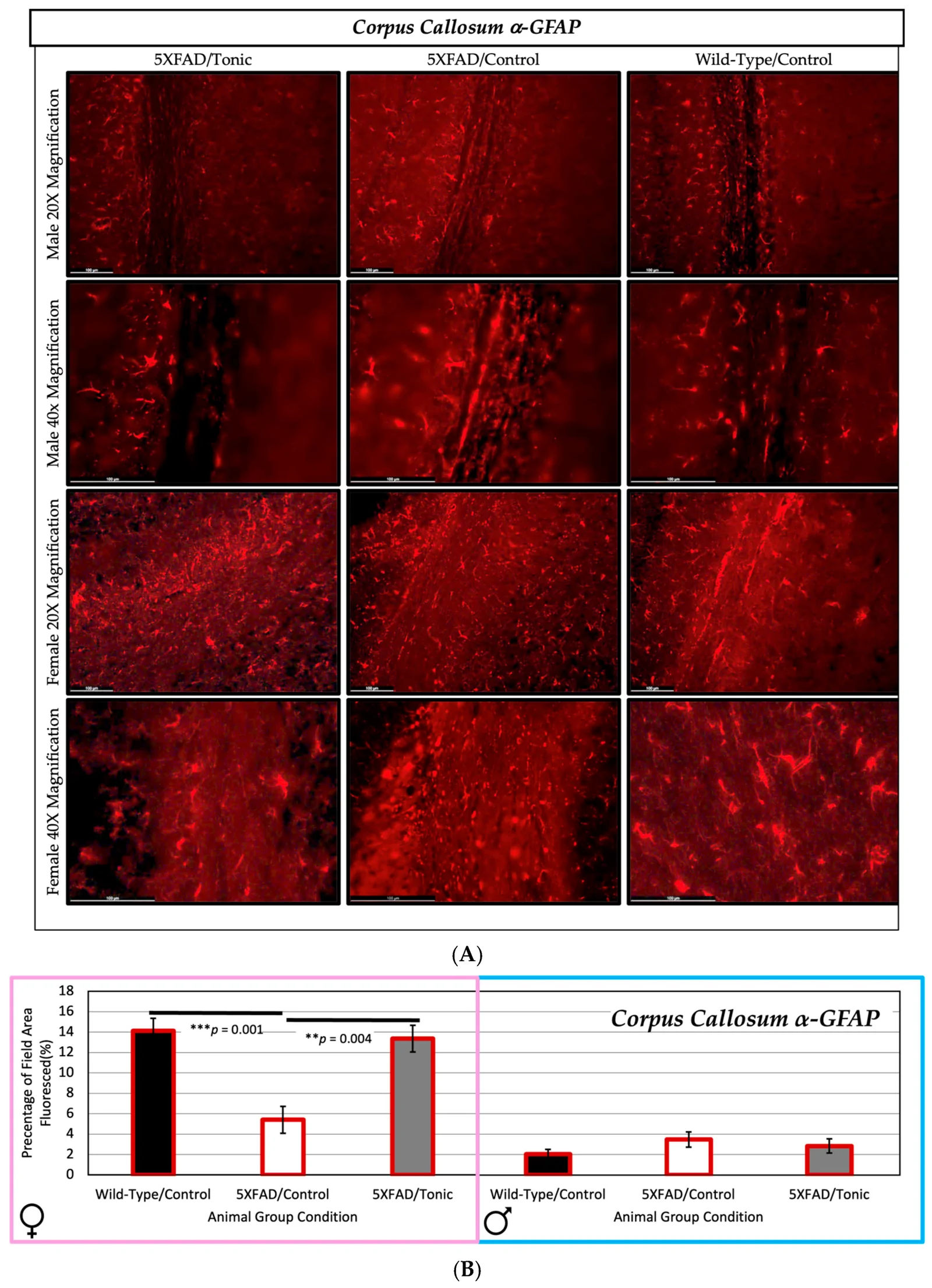
(**A**) Representative immunofluorescence images (20× and 40× magnification; scale bar = 100 μm) and (**B**) quantitative analysis of α-glial fibrillary acidic protein (α-GFAP) expression within the corpus callosum of male (blue) and female (pink) wild-type/control, 5XFAD/control, and 5XFAD/Tonic-treated mice. Immunoreactivity was quantified as the percentage of imaging field area that met or surpassed threshold fluorescence using ImageJ (*n* = 3 mice per group, 3 fields per mouse). Data are presented as mean ± SEM. Statistical significance was determined using one-way analysis of variance (ANOVA) followed by Bonferroni-corrected Welch’s *t*-tests. ** *p* < 0.01, *** *p* < 0.001.

Corpus callosum α-GFAP immunoreactivity, as represented in Figure 16A,B, demonstrated a distinct sex-dependent response. No significant differences were observed among male groups (*F*(2,24) = 1.22, *p* = 0.31, η^2^ = 0.09). In contrast, female mice exhibited significant differences (*F*(2,23) = 11.60, *p* = 0.0003, η^2^ = 0.50). Untreated female 5XFAD/control mice demonstrated substantially reduced corpus callosum α-GFAP immunoreactivity (M = 6.03 ± 1.32%) compared with both wild-type/control (M = 14.112 ± 1.22%) and 5XFAD/Tonic-treated mice (M = 13.34 ± 1.31%). Tonic treatment restored α-GFAP expression to levels comparable with wild-type/control animals.

Qualitatively looking at the 40× magnification representative images (Figure 16A), the male 5XFAD/control mice show typical active-astrocyte morphological characteristics with thicker and less branched processes, enlarged cell bodies, and increased fluorescent expression. On the other hand, male and female 5XFAD/Tonic and wild-type/control mice show more even distribution of astrocytes, with thin, complex processes, consistent with the homeostatic astrocytic state. However, female 5XFAD/control mice show fragmented GFAP fluorescent expression, which may indicate astrocyte atrophy or damage.

### 3.4. Golgi–Cox Staining Results

**Figure 17:**
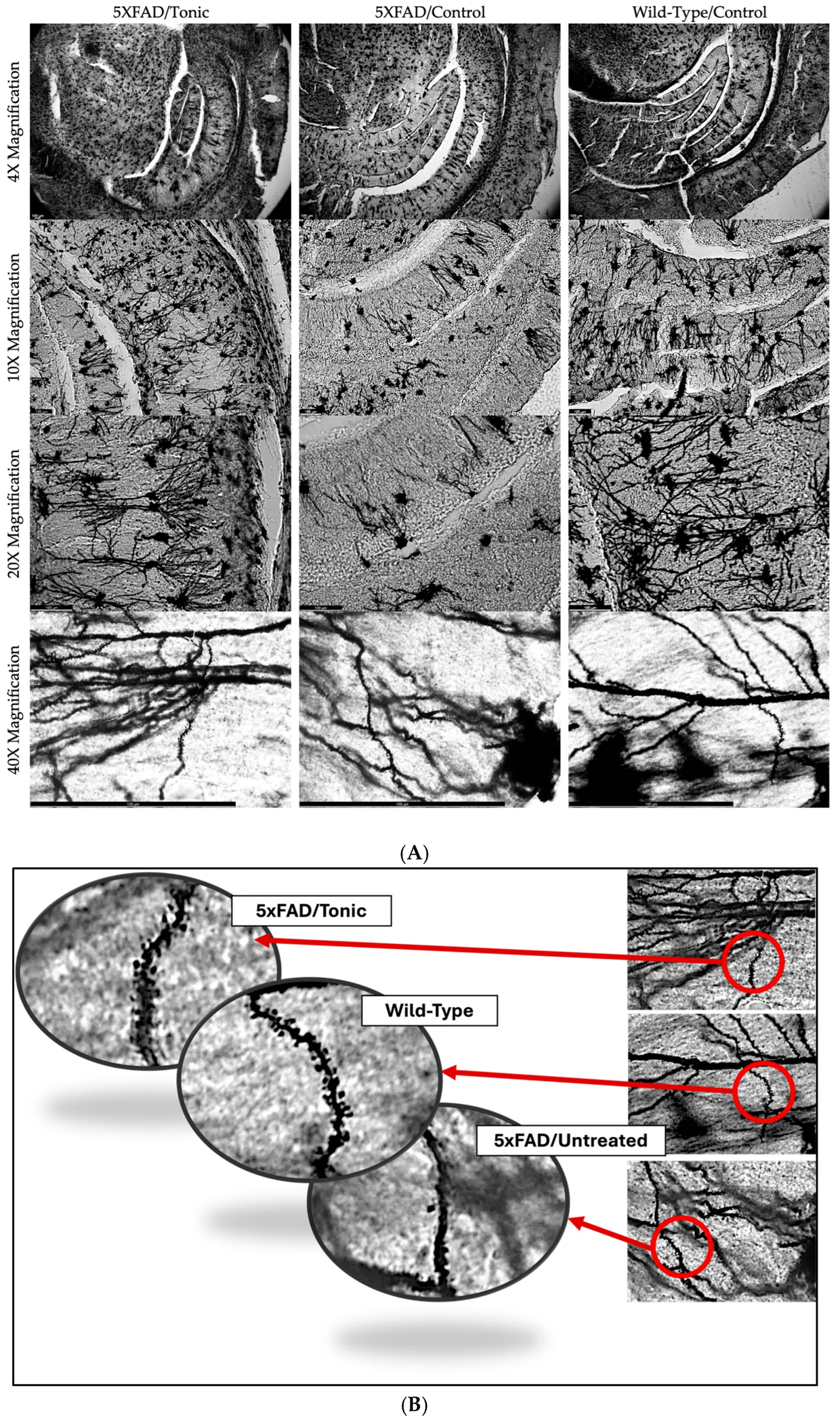
Representative images of dendritic morphology in male 5XFAD/Tonic, 5XFAD/untreated control, and wild-type/control mice following Golgi–Cox staining. (**A**) Images are shown at 4×, 10×, 20×, and 40× magnification to demonstrate dendritic architecture and spine morphology across groups. Scale bar = 100 µm. (**B**) Higher-magnification images highlight individual dendritic segments and associated dendritic spines. Enlarged regions of interest (red circles) are shown in the lower panel for each experimental group, with arrows indicating the corresponding dendritic segments. Representative images demonstrate differences in dendritic structure and spine morphology between untreated 5XFAD mice and Tonic-treated and wild-type mice.

Figure 17A provides representative images of Golgi–Cox-stained dendrites from 5XFAD/Tonic, 5XFAD/control, and wild-type/control mice at increasing magnifications. Figure 17B gives a closer visual as to the dendritic architecture used for the current quantitative analyses. Refer to Supplementary Table S4. for ANOVA summary of results. Pairwise Bonferroni-corrected comparisons are presented in Supplementary Table S4.

#### 3.4.1. Percentage of Mature Dendrites

**Figure 18:**
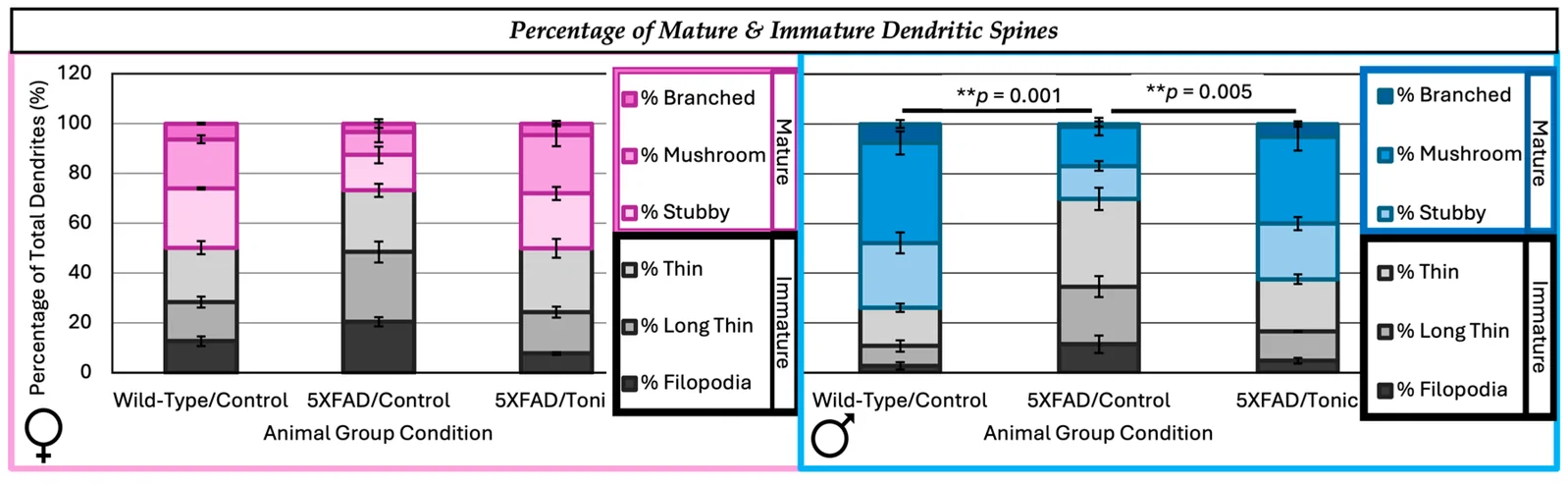
Percentage of dendritic spines classified as branched, mushroom, stubby, thin, long-thin, and filopodia in male (blue) and female (pink) wild-type/control, 5XFAD/control, and Tonic-treated 5XFAD mice (*n* = 3 mice per group condition, 3 microscope image fields per mice). Branched and mushroom spines were categorized as mature spines, whereas stubby, thin, long thin, and filopodia were classified as immature spines according to established morphological criteria. Data are presented as the percentage of total dendritic spines (mean ± SEM). Statistical significance was determined using one-way analysis of variance (ANOVA) followed by Bonferroni-corrected Welch’s *t*-tests. ** *p* < 0.01.

Figure 18 shows the results of Golgi–Cox staining quantification for percentage of mature (branched, mushroom, stubby) vs. immature (thin, long thin, filopodia) dendritic spine morphology. In female mice, treatment significantly affected the proportion of mature dendrites (one-way ANOVA, *F*(2,6) = 6.27, *p* = 0.03, η^2^ = 0.68). Tonic versus untreated 5XFAD showed a very large standardized difference (*d* = 2.067), while Tonic versus wild-type was negligible (*d* = 0.030). Mean mature dendrite proportions were 0.27 ± 0.07 in 5XFAD/control mice, 0.50 ± 0.06 in Tonic-treated 5XFAD mice, and 0.50 ± 0.01 in wild-type/controls.

In male mice, treatment also significantly affected the proportion of mature dendrites (one-way ANOVA, *F*(2,6) = 68.00, *p* < 0.001, η^2^ = 0.96). 5XFAD/Tonic versus 5XFAD/control showed an extremely large difference (*d* = 6.283), while Tonic versus wild-type remained large (*d* = −2.554) but was not significant. The 5XFAD/control versus wild-type/control comparison was also extremely large (*d* = −9.429). These pairwise effect sizes indicate substantial separation among the male groups despite the nonsignificant Tonic versus wild-type comparison. Mean mature dendrite proportions were 0.30 ± 0.03 in 5XFAD/control mice, 0.62 ± 0.03 in Tonic-treated 5XFAD mice, and 0.74 ± 0.02 SEM in wild-type controls.

The effect sizes indicate that experimental group accounted for a sizeable proportion of the variation in mature/immature dendrites in both sexes, with particularly prominent group-associated differences in males. The large pairwise separation between untreated and Tonic-treated 5XFAD mice, together with the similarity between Tonic-treated and wild-type/control mice in the corresponding comparisons, is consistent with substantial preservation or restoration of mature dendritic structure following Tonic treatment.

#### 3.4.2. Dendritic Length

**Figure 19:**
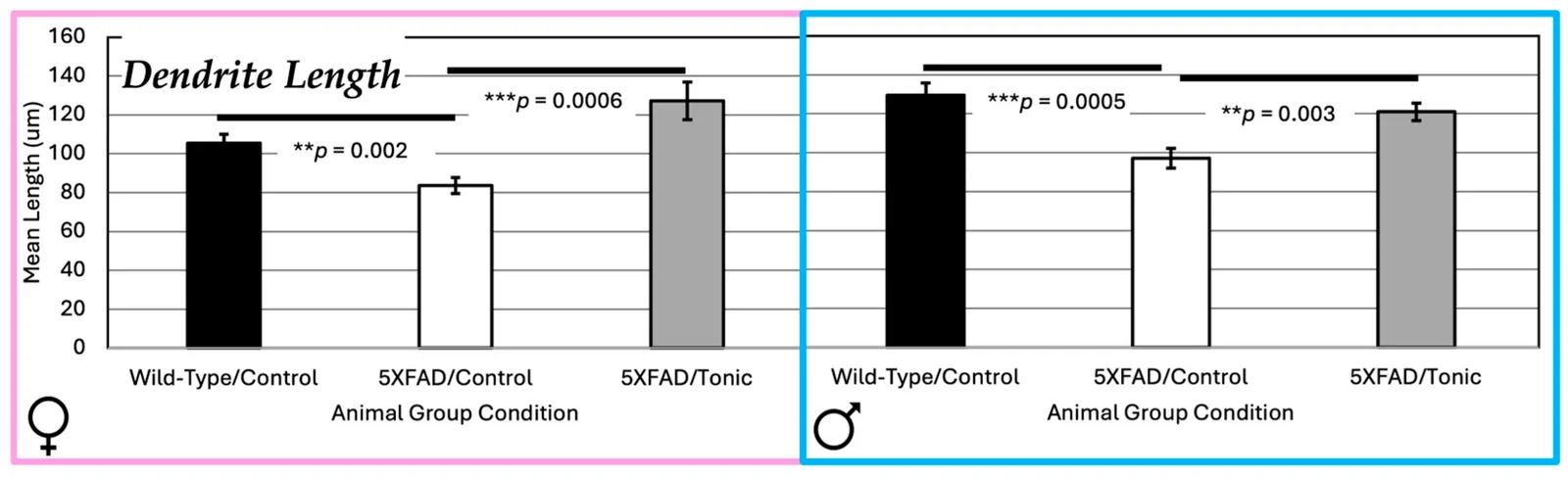
Mean dendritic length in Golgi–Cox-stained hippocampal neurons from male (blue) and female (pink) wild-type/control, 5XFAD/control, and Tonic-treated 5XFAD mice (*n* = 3 mice per group condition, 3 microscope image fields per mice). Dendritic length was measured using ImageJ after calibration with a 100 μm scale bar. Data are presented as mean ± SEM. Statistical significance was determined using one-way analysis of variance (ANOVA) followed by Bonferroni-corrected Welch’s *t*-tests. ** *p* < 0.01, *** *p* < 0.001.

Figure 19 displays the results for dendritic length quantification for males and females via Golgi–Cox staining. Treatment significantly affected dendritic length in female mice (one-way ANOVA, *F*(2,78) = 10.90, *p* < 0.001, η^2^ = 0.22). Mean dendritic length was 83.56 ± 4.58 μm in 5XFAD/control mice, 127.22 ± 9.65 μm in Tonic-treated 5XFAD mice, and 105.49 ± 4.58 μm in wild-type/controls. Tonic versus untreated 5XFAD/control showed a large effect (*d* = 1.132), while 5XFAD/Tonic versus wild-type/control was moderate (*d* = 0.554) and nonsignificant. The pairwise effects indicate that Tonic-treated mice had greater dendritic length than untreated 5XFAD mice, while their measurements remained relatively similar to wild-type/control mice.

Similarly, treatment significantly affected dendritic length in male mice (one-way ANOVA, *F*(2,78) = 10.17, *p* < 0.001, η^2^ = 0.21). Mean dendritic length was 97.07 ± 5.10 μm in 5XFAD/control mice, 121.04 ± 4.46 μm in Tonic-treated 5XFAD mice, and 129.54 ± 6.15 μm in wild-type/controls. Tonic versus untreated 5XFAD showed a large effect (*d* = 0.963), whereas Tonic versus wild-type was small (*d* = −0.305) and nonsignificant. Thus, the treated groups were relatively close to wild-type for dendritic length and had substantial treatment-associated differences from untreated 5XFAD/control mice.

#### 3.4.3. Dendritic Thickness

**Figure 20:**
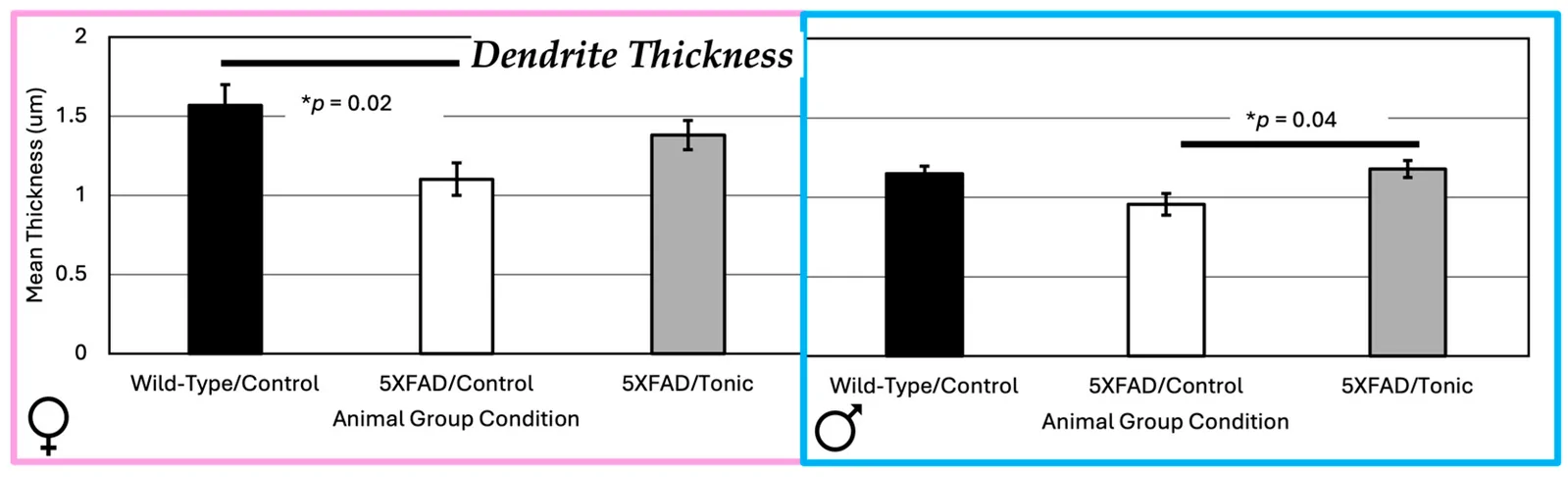
Mean dendritic thickness in Golgi–Cox-stained hippocampal neurons from male (blue) and female (pink) wild-type/control, 5XFAD/control, and Tonic-treated 5XFAD mice (*n* = 3 mice per group condition, 3 microscope image fields per mice). Dendritic thickness was quantified using ImageJ following calibration with a 100 μm scale bar. Data are presented as mean ± SEM. Statistical significance was determined using one-way analysis of variance (ANOVA) followed by Bonferroni-corrected Welch’s *t*-tests. *p* < 0.05 was considered statistically significant. * *p* < 0.05.

Figure 20 shows the results of male and female quantification of dendritic thickness via Golgi–Cox staining for dendritic morphology. Treatment significantly effected dendritic thickness in female mice (one-way ANOVA, *F*(2,78) = 4.52, *p* = 0.01, η^2^ = 0.10). 5XFAD/Tonic versus untreated 5XFAD/control showed a moderate effect (*d* = 0.546) but was nonsignificant, while 5XFAD/Tonic versus wild-type/control was small (*d* = −0.318). The untreated versus wild-type/control comparison was moderate in females (*d* = −0.759), which indicates that experimental group accounted for a comparatively limited proportion of the variation in dendritic thickness. Mean dendritic thickness was 1.10 ± 0.10 μm in 5XFAD/control mice, 1.38 ± 0.09 μm in Tonic-treated 5XFAD mice, and 1.57 ± 0.13 μm in wild-type/controls, with pairwise comparisons suggesting that 5XFAD/Tonic and wild-type/control dendritic thickness having limited separation.

A significant treatment effect was also observed in male mice (one-way ANOVA, *F*(2,78) = 4.40, *p* = 0.02, η^2^ = 0.10). Tonic versus untreated 5XFAD showed a moderate effect (*d* = 0.696), whereas Tonic versus wild-type was negligible (*d* = 0.114), and the untreated versus wild-type/control comparison was small (*d* = −0.626). Therefore, results suggest treatment had a modest influence on dendritic thickness. Mean dendritic thickness was 0.96 ± 0.07 μm in 5XFAD/control mice, 1.179 ± 0.05 μm in Tonic-treated 5XFAD mice, and 1.14 ± 0.04 μm in wild-type/controls. Similar to females, pairwise comparisons indicate that wild-type/control and 5XFAD/Tonic groups were relatively similar in their dendritic thickness.

#### 3.4.4. Dendritic Spine Density

**Figure 21:**
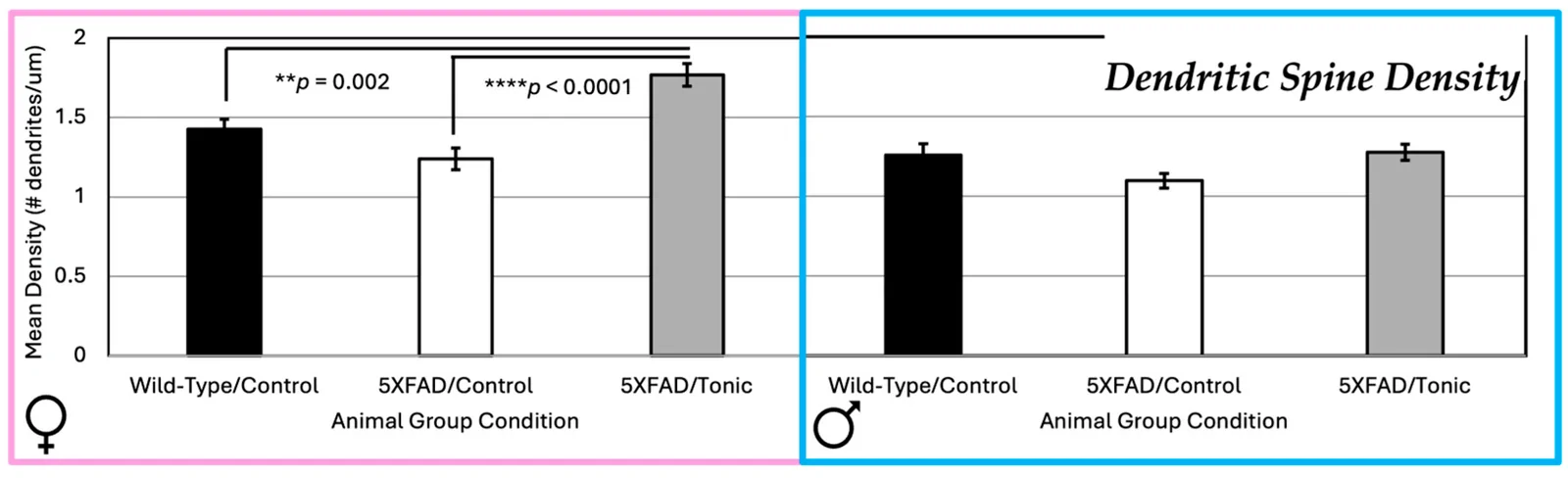
Mean dendritic density in Golgi–Cox-stained hippocampal neurons from male (blue) and female (pink) wild-type/control, 5XFAD/control, and Tonic-treated 5XFAD mice (*n* = 3 mice per group condition, 3 microscope image fields per mice). Density was quantified as the number of dendrites per micrometre using ImageJ. Data are presented as mean ± SEM. Statistical significance was determined using one-way analysis of variance (ANOVA) followed by Bonferroni-corrected Welch’s *t*-tests. ** *p* < 0.01, **** *p* < 0.0001.

Figure 21 represents the results of male and female Golgi–Cox quantification of dendritic spine density. Female mice exhibited a significant treatment effect on dendritic spine density (one-way ANOVA, *F*(2,78) = 15.66, *p* < 0.001, η^2^ = 0.29). The female effect was large with a very large 5XFAD/Tonic versus untreated 5XFAD/control difference (*d* = 1.464). Tonic versus wild-type was also large (*d* = 0.969), while 5XFAD/control versus wild-type/control was moderate (*d* = −0.545). Mean spine density was 1.24 ± 0.07 spines/μm in 5XFAD/control mice, 1.77 ± 0.07 spines/μm in Tonic-treated 5XFAD mice, and 1.43 ± 0.06 spines/μm in wild-type/controls.

No significant differences in dendritic spine density were observed in male mice (one-way ANOVA, *F*(2,78) = 2.98, *p* = 0.06). The omnibus effect was moderate (η^2^ = 0.07). 5XFAD/Tonic versus 5XFAD/control showed a medium-to-large effect (*d* = 0.711), whereas Tonic versus wild-type was negligible (*d* = 0.054) and untreated versus wild-type was moderate (*d* = −0.516). Mean spine density was 1.10 ± 0.05 spines/μm in 5XFAD/control mice, 1.28 ± 0.05 spines/μm in Tonic-treated 5XFAD mice, and 1.26 ± 0.07 spines/μm in wild-type/controls.

The effect sizes indicate that group-associated variation in spine density was more prominent in females than males, suggesting that Tonic treatment had a greater influence on dendritic spine density in female mice. Surprisingly, pairwise effects in females indicate greater spine density following Tonic treatment relative to both untreated 5XFAD mice and wild-type, whereas the comparatively small male effect suggests that spine density was less strongly associated with experimental group in males.

### 3.5. Frailty Index Results

**Figure 22:**
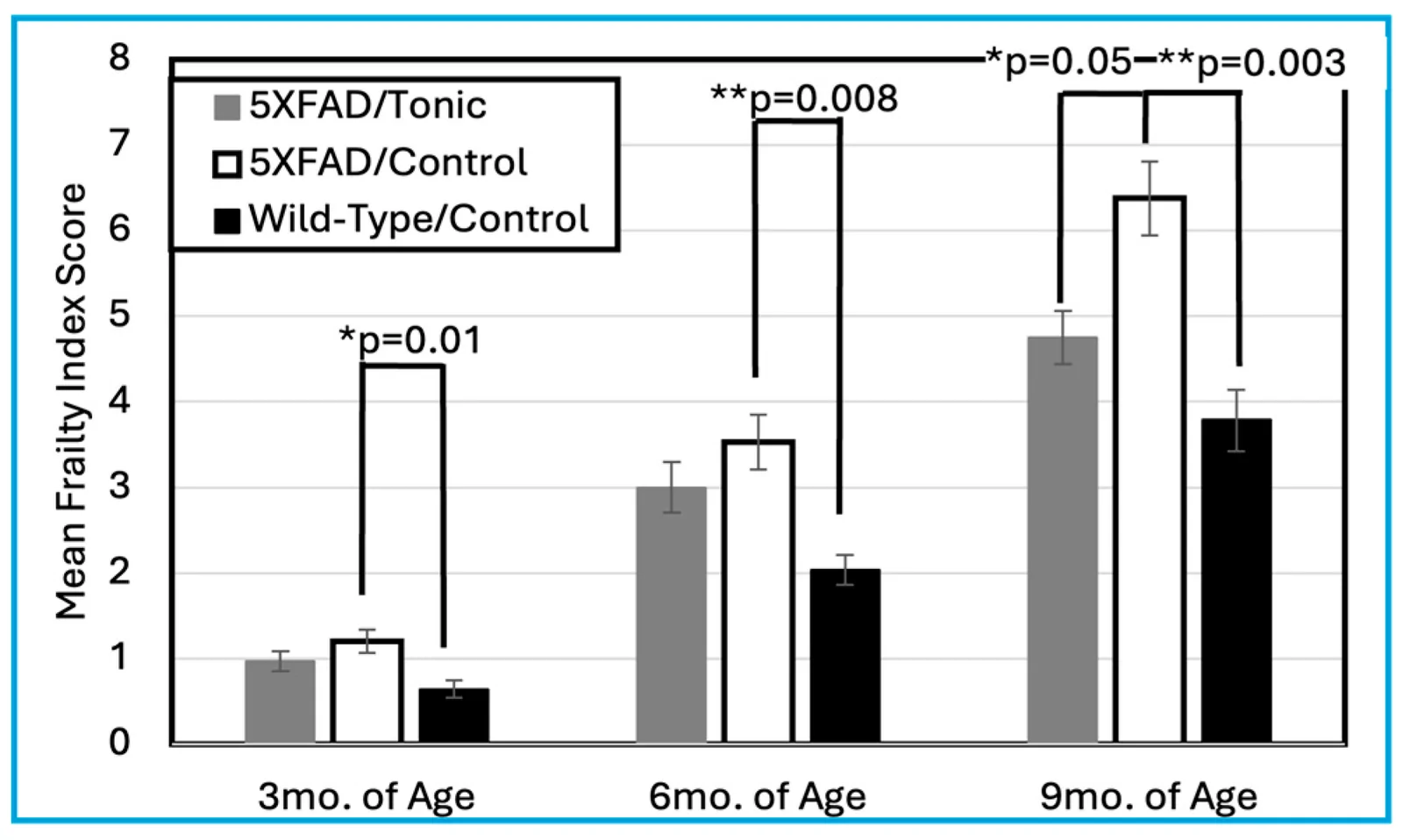
Longitudinal frailty index scores of male wild-type/control, 5XFAD/control, and Tonic-treated 5XFAD mice assessed at 3, 6, and 9 months of age (*n* = 7–8 mice per group condition). Frailty was evaluated using the 31-item mouse frailty index and expressed as the normalized frailty score (mean ± SEM). Between-group comparisons at each time point were analyzed using two-way repeated measures analysis of variance (ANOVA) followed by Bonferroni-corrected comparisons for between-group differences. * *p* < 0.05, ** *p* < 0.01.

Frailty scores increased progressively across the three assessment timepoints in all experimental groups (Figure 22). A two-way repeated-measures ANOVA demonstrated significant main effects of time (*F*(1.69, 33.74) = 138.10, *p* < 0.0001, partial η^2^ = 0.87) and group condition (*F*(2,20) = 19.89, *p* < 0.0001, partial η^2^ = 0.50). A significant time × group interaction was also observed (*F*(3.37, 33.74) = 3.22, *p* = 0.030, partial η^2^ = 0.24), indicating that the progression of frailty differed between experimental groups over time. The Geisser–Greenhouse correction was applied to account for violations of sphericity (ε = 0.84). The longitudinal time effect was very large (partial η^2^ = 0.87; *f* = 2.63), demonstrating substantial age-related progression in frailty. The overall group effect was large (partial η^2^ = 0.50; *f* = 1.00), while the time × group interaction was also large (partial η^2^ = 0.57; *f* = 0.57), indicating that the trajectory of frailty differed according to group condition.

At 3 months of age, mean frailty scores were 0.97 ± 0.19 SEM for 5XFAD/Tonic mice, 1.20 ± 0.19 SEM for 5XFAD controls, and 0.64 ± 0.16 SEM for wild-type controls. Bonferroni-adjusted comparisons revealed a significantly higher frailty score in 5XFAD controls compared with wild-type controls (mean difference = 0.56, 95% CI: 0.06–1.06, Bonferroni-adjusted *p* = 0.03), whereas neither the 5XFAD/Tonic group differed from 5XFAD controls (adjusted *p* = 0.72) nor wild-type controls (adjusted *p* = 0.19).

At 6 months of age, frailty scores increased to 3.00 ± 0.46 SEM in 5XFAD/Tonic mice, 3.53 ± 0.46 SEM in 5XFAD/controls, and 2.04 ± 0.39 SEM in wild-type/controls. 5XFAD/controls exhibited significantly greater frailty than wild-type/controls (mean difference = 1.49, 95% CI: 0.39–2.59, adjusted *p* = 0.009). The 5XFAD/Tonic group did not differ significantly from either 5XFAD/controls (adjusted *p* = 0.83) or wild-type/controls (adjusted *p* = 0.07).

At 9 months of age, frailty scores further increased to 4.75 ± 0.57 SEM in 5XFAD/Tonic mice, 6.38 ± 0.57 SEM in 5XFAD/controls, and 3.79 ± 0.51 SEM in wild-type/controls. 5XFAD/controls exhibited significantly greater frailty than both the 5XFAD/Tonic group (mean difference = 1.63, 95% CI: 0.05–3.20, adjusted *p* = 0.04) and wild-type/controls (mean difference = 2.59, 95% CI: 0.92–4.26, adjusted *p* = 0.003). The 5XFAD/Tonic and wild-type/control groups did not differ significantly (adjusted *p* = 0.25).

Overall, the significant time × group interaction indicates that frailty progression was influenced by group condition, with untreated 5XFAD/Control mice exhibiting the greatest increase in frailty over time. The comparatively lower frailty scores observed in 5XFAD/Tonic mice at 9 months suggest that Tonic treatment shifted the age-related increase in frailty observed in untreated 5XFAD mice.

Refer to Supplementary Table S5 for the ANOVA summary of results. Pairwise Bonferroni-corrected comparisons of between-subject results are presented in Supplementary Table S5. Supplementary Table S5 provides representative photos of how the criteria was applied to three individual mice at 9 months of age to give an example of the variation in aging outcomes in a high-scored mouse (5XFAD/control), moderate-scored mouse (5XFAD/Tonic), and low-scored mouse (wild-type/control).

### 3.6. Spatial and Non-Spatial Memory Analysis Using the Novel Object Recognition and Novel Location Recognition Tests

**Figure 23:**
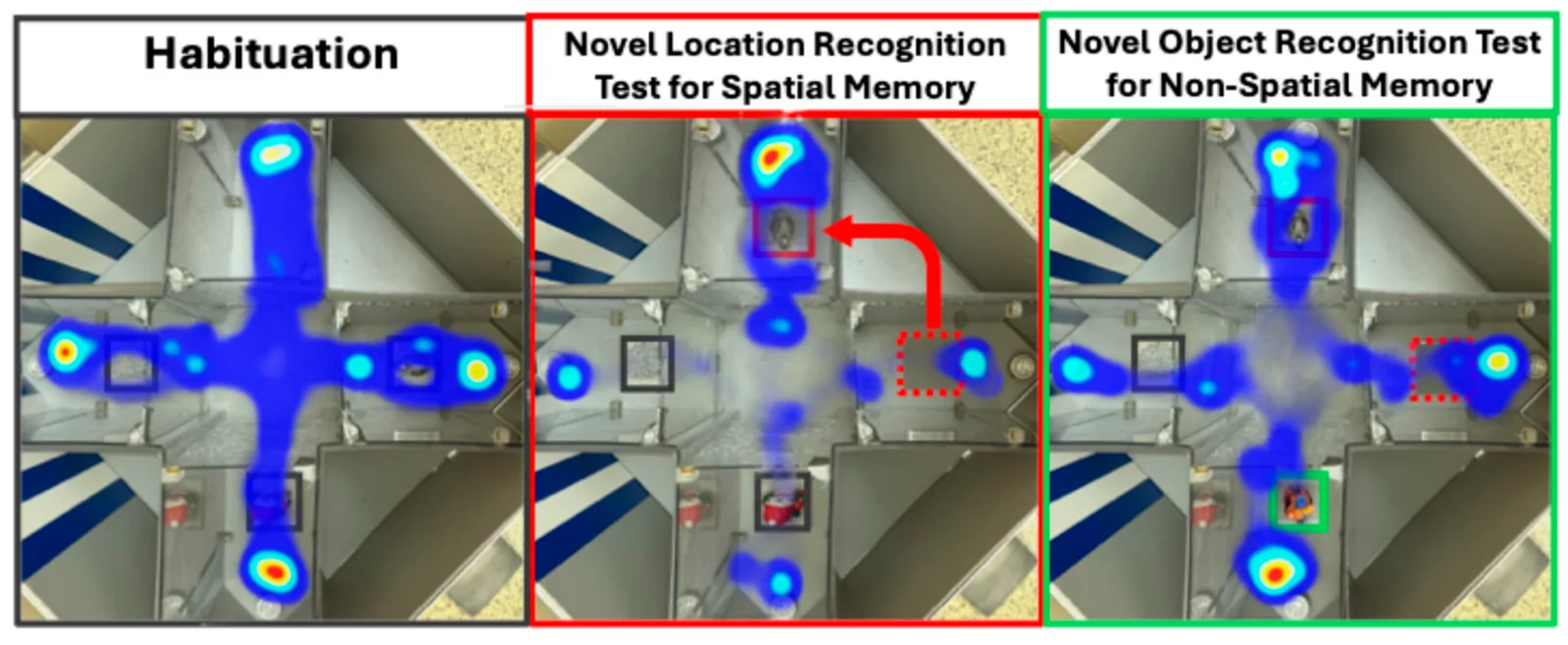
Example of one mouse’s heatmap during habituation, Novel Location Recognition, and Novel Object Recognition test, overlayed on representative images of object placements and manipulations on habituation and test trials (Figure 5). The color around each image of the maze corresponds with color of the box around the object and arrow representing the manipulation in that test. The heatmap is produced based on the amount of time the mouse spent in each coordinate of the maze per trial. Colors span from blue, representing low rate of occupation, to red, representing high rate of occupation.

Figure 23 gives an example of a heatmap of one mouse’s exploration behavior across habituation, NLR, and NOR test trials to show typical exploratory behavior during these novelty-recognition tasks.

For the behavioral/cognitive experiments, the reported Cohen’s *d* values represent within-subject changes between task conditions or trials. They therefore indicate the magnitude of the animals’ behavioral response to the relevant task manipulation and should not be interpreted as direct treatment-effect sizes.

#### 3.6.1. Habituation to the Testing Arena

**Figure 24:**
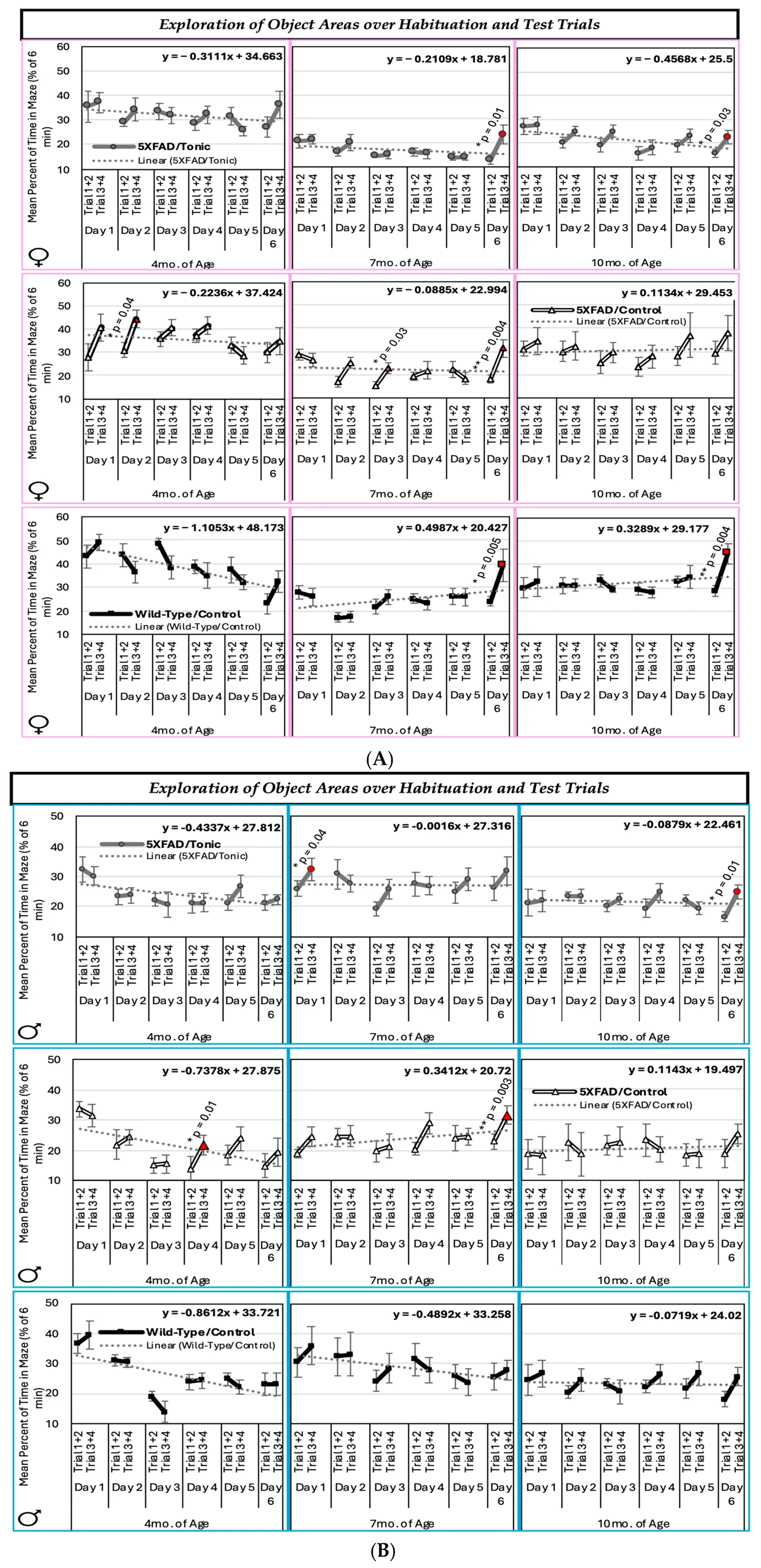
(**A**) Female and (**B**) male exploration behavior in all object areas over habituation days 1–5 and testing (novel object recognition and novel location recognition test) Day 6. Total exploration time across all object regions was compared between the first two trials (ΣTrials 1 + 2 exploration) and the final two trials (ΣTrials 3 + 4 exploration) for each day (Days 1–6) at 4, 7, and 10 months of age in Tonic-treated 5XFAD, 5XFAD/control, and wild-type/control mice. Data are presented as mean percentage of the total 6 min exploration period (mean ± SEM). Statistical significance was determined using paired samples *t*-tests. * *p* < 0.05, ** *p* < 0.01.

Figure 24A,B shows results of female and male mice exploratory behavior in all object areas over habituation Days 1–5 and testing Day 6. Habituation was assessed by comparing total object exploration during the first two habituation trials (Trials 1 + 2) with the final two habituation trials (Trials 3 + 4) using paired-samples *t*-tests for each testing day.

Across the 6-day habituation period, exploration generally remained stable within treatment groups, indicating successful acclimation to the testing environment. However, in female mice, 5XFAD/control animals showed little evidence of progressive habituation across testing timepoints, with linear slopes of −0.22, −0.09, and 0.11 at 4 months, 7 months, and 10 months of age, respectively. Consistent with these relatively flat or positive trajectories, untreated female 5XFAD mice had significant increases in exploration on Day 2 of habituation at 4 months of age (*t*(6) = −2.58, *p* = 0.04, *d* = −0.572) and Day 3 of habituation at 7 months of age (*t*(6) = −2.80, *p* = 0.03, *d* = −0.655), with large with-in subject response sizes. Therefore, this reduces the assumption that any differences seen on testing trials is due to recognition of novelty, such as their significant increase and large within-subject magnitude of exploration at 7 months of age Day 6 (*t*(6) = −4.48, *p* = 0.004, *d* = −0.917). In contrast, female Tonic-treated 5XFAD mice demonstrated consistently negative linear slopes across all three testing timepoints (−0.31, −0.21, and −0.46, respectively), indicative of a more consistent reduction in exploration across repeated testing. Wild-type/control females demonstrated a steeper decline during the first timepoint (slope = −1.01), but positive slopes at the second and third timepoints (0.50 and 0.33, respectively). No significant differences in exploration were observed in 5XFAD/Tonic-treated or wild-type/control females across the habituation period, which is evidence of habituation and therefore reinforces that significant increases in exploration on testing trials is due to recognition of novelty. On that note, female 5XFAD/Tonic and wild-type/control stood alone as producing statistically increased exploration when introduced to novelties on Day 6 at 7 months of age (5XFAD/Tonic, *t*(6) = −3.50, *p* = 0.01, *d* = −1.167; wild-type/control, *t*(7) = −2.39, *p* = 0.05, *d* = −0.653) and 10 months of age (5XFAD/Tonic, *t*(6) = −2.89, *p* = 0.03, *d* = −0.978; wild-type/control, *t*(7) = −4.20, *p* = 0.004, *d* = −0.768), and their large effect sizes further suggest that these groups displayed substantial changes in their behavior when exposed to novelty.

Male mice generally demonstrated a more typical habituation pattern. At 4 months of age, all male groups exhibited negative linear slopes, with values of −0.43, −0.74, and −0.86 for Tonic-treated 5XFAD, untreated 5XFAD/control, and wild-type/control mice, respectively. At 7 months of age, the 5XFAD/control group showed a positive slope (0.341), whereas the Tonic-treated 5XFAD and wild-type/control mice showed slopes of −0.0016 and −0.489, respectively. A similar pattern was observed at 10 months of age, with a positive slope in 5XFAD/control males (0.114) compared with negative slopes in Tonic-treated 5XFAD (−0.0879) and wild-type/control mice (−0.0719). Male 5XFAD/control mice exhibited greater exploration on Day 4 at 4 months of age (*t*(7) = −3.54, *p* = 0.009, *d* = −1.219), which impacts the interpretation of any increased exploration on testing trials, especially considering the large effect size. Male Tonic-treated 5XFAD mice demonstrated increased exploration on 7 months of age Day 1 (*t*(7) = −2.61, *p* = 0.04, *d* = −0.648), which is typical of re-introduction to the maze. On testing Day 6, male 5XFAD/control animals had significantly increased exploration at 7 months of age when introduced to novelty (*t*(7) = −4.36, *p* = 0.003, *d* = −1.053), and 5XFAD/Tonic had significantly increased exploration to novelty at 10 months of age (*t*(7) = −3.37, *p* = 0.001, *d* = −1.407). Wild-type/control male mice did not show any significant differences in exploration during any timepoint or day.

Together, these results suggest that female 5XFAD/Tonic and wild-type/control mice showed more consistent habituation through significant differences in exploration seen exclusively when novelty was introduced. 5XFAD/control female mice showed seemingly random significant differences in exploration on habituation days with no novelty to respond to and did not show any significant difference in exploration on any day (including testing day) during the final testing timepoint at 10 months of age. In contrast, habituation was more evident in male mice through consistently negative slope over habituation days in 5XFAD/Tonic and wild-type/control groups. Although male 5XFAD/control mice had a negative slope during the first testing timepoint, it became positive at 7 months of age and continued in that direction at 10 months of age. Male 5XFAD/Tonic and wild-type/control mice consistently displayed reduced exploration over habituation trials throughout all testing timepoints.

Complete habituation *t*-test statistics for all testing days are presented in Supplementary Table S6.

#### 3.6.2. Spatial Memory: NLR

**Figure 25:**
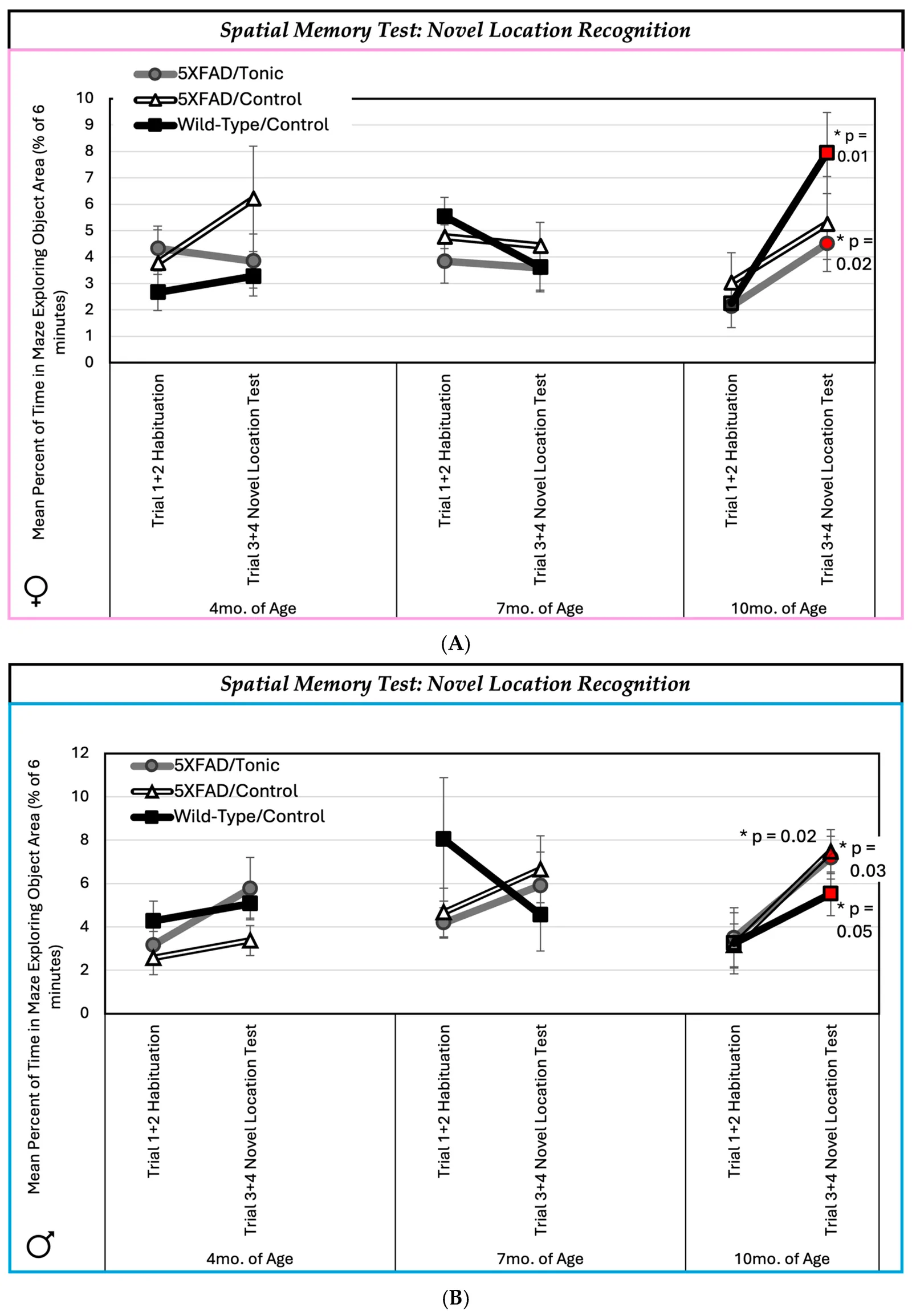
Spatial memory performance assessed using the Novel Location Recognition (NLR) task. Exploration time within the object region containing the relocated object was compared between habituation (ΣTrials 1 + 2 exploration) and testing (ΣTrials 3 + 4 exploration) at 4, 7, and 10 months of age in (**A**) female and (**B**) male mice. Data are presented as the mean percentage of the 6 min exploration period (mean ± SEM). Statistical significance was determined using paired samples *t*-tests. * *p* < 0.05.

Spatial memory was assessed on Day 6 by relocating one familiar object to a novel location, as shown in Figure 25A,B. Exploration of the relocated object location was compared between the habituation (Trials 1 + 2) and testing (NLR; Trials 3 + 4) phases using paired-samples *t*-tests.

Female 5XFAD/control mice did not display any significant changes to novel location-specific object exploration on Day 6 (all *p* > 0.05), however, 5XFAD/Tonic and wild-type/control female mice had significantly increased exploration in response to the spatial novelty at 10 months of age (5XFAD/Tonic, *t*(6) = −3.02, *p* = 0.02, *d* = −1.183; wild-type/control, *t*(7) = −3.56, *p* = 0.009, *d* = −1.715), with very large effect sizes indicating that their magnitude of response to the moved object was strong. Male mice showed more variation in their response to spatial novelty, and all mice showed a significant increase in exploration to this manipulated object at 10 months of age Day 6 (5XFAD/Tonic, *t*(7) = −2.76, *p* = 0.03, *d* = −1.021; 5XFAD/control, *t*(7) = −3.13, *p* = 0.02, *d* = −1.425; wild-type/control, *t*(6) = −2.47, *p* = 0.05, *d* = −0.649), with large effect sizes across groups, either indicating a sex-dependant exploratory pattern or lesser memory disruption in male mice at equivalent timepoints to females. Detailed object-specific statistics are summarized in Supplementary Table S6.

#### 3.6.3. Non-Spatial Memory: NOR

**Figure 26:**
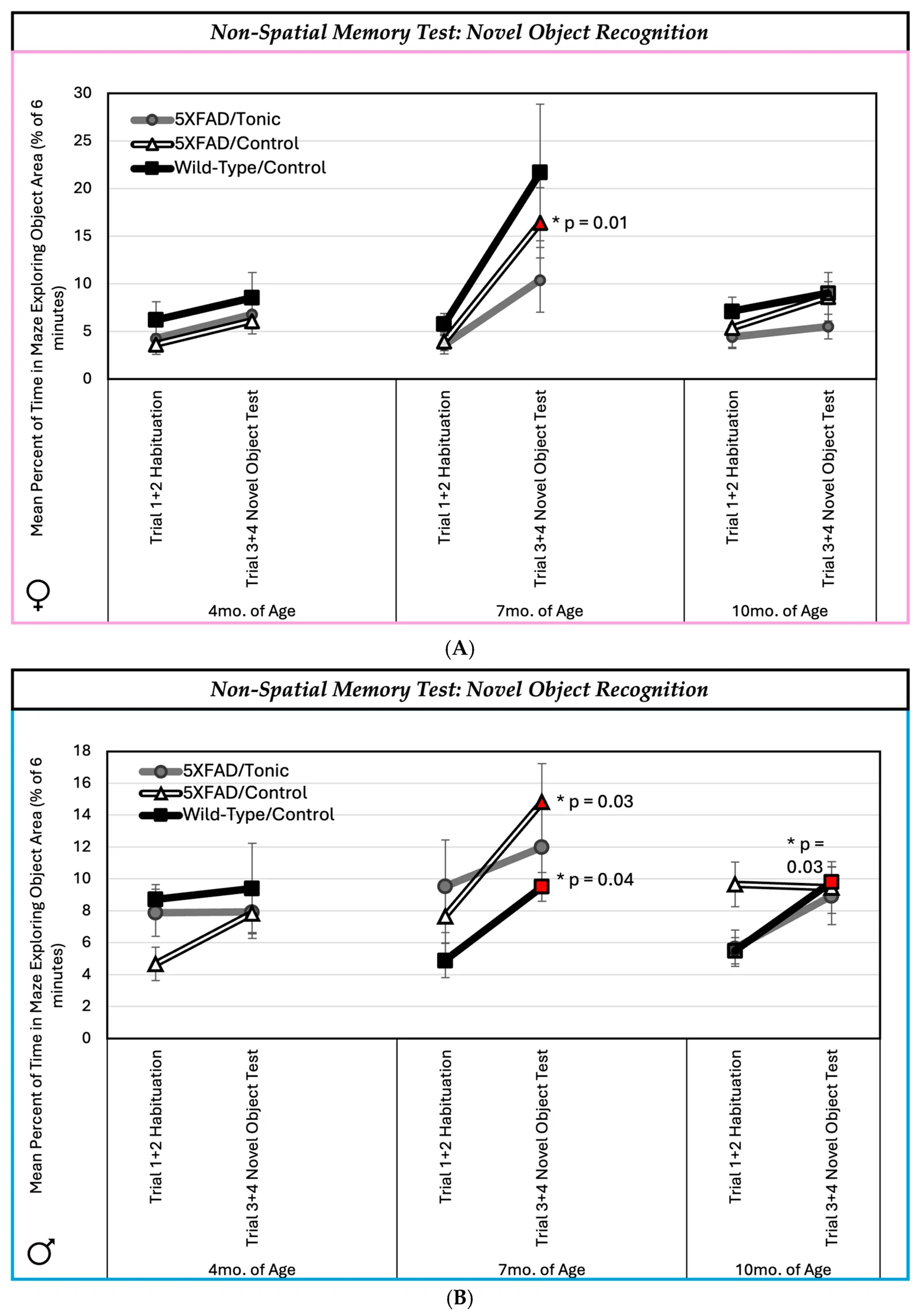
Non-spatial memory performance assessed using the Novel Object Recognition (NOR) task. Exploration time within the object region containing the novel object was compared between habituation (ΣTrials 1 + 2 exploration) and testing (ΣTrials 3 + 4 exploration) at 4, 7, and 10 months of age in (**A**) female and (**B**) male mice. Data are presented as the mean percentage of the 6 min exploration period (mean ± SEM). Statistical significance was determined using paired samples *t*-tests. * *p* < 0.05.

Non-spatial recognition memory was evaluated on Day 6 by replacing one familiar object with a novel object, as shown in Figure 26A,B. Total exploration during the habituation (Trials 1 + 2) and testing (NOR) trials (3 + 4) did not differ significantly between Tonic-treated 5XFAD, 5XFAD/control, or wild-type/control mice (all *p* > 0.05).

Female mice displayed less consistent response to novel object recognition on testing days. 5XFAD/control mice stood alone as having significantly different exploration of the non-spatial novelty at 7 months of age Day 6 (*t*(6) = −3.65, *p* = 0.01, *d* = −1.626), with a very large effect size indicating their magnitude of behavioral change was strong in response to the novel object. On the other hand, male 5XFAD/Tonic and wild-type/control mice remained with generally increased exploration of the novel object on testing Day 6, whereas 5XFAD/control showed little to no response at 4 months and 10 months of age. However, wild-type/control mice were the only cohort to show significant differences and very large magnitudes of exploratory change in response to the object manipulation at 7 months of age (*t*(6) = −2.62, *p* = 0.04, *d* = −1.634) and 10 months of age (*t*(6) = −2.93, *p* = 0.03, *d* = −1.687). 5XFAD/control had significant recognition to non-spatial novelty on Day 6 at 7 months of age (*t*(7) = −2.62, *p* = 0.04, *d* = −1.131), but at 10 months of age, their exploration did not change in response to the novel object. Complete paired-samples *t*-test statistics for each object region are presented in Supplementary Table S6.

## 4. Discussion

AD is a complex neurodegenerative disorder characterized by the interaction of multiple pathological processes, including amyloid accumulation, neuroinflammation, oxidative and mitochondrial dysfunction, impaired proteostasis, synaptic degeneration, and progressive loss of neuronal and systemic function [7,8,9,10,11,12,13,14,15,16,17]. The interconnected nature of these processes presents a significant challenge for therapeutic development, since targeting a single pathology may not be sufficient to address most of the complex biological disturbances that contribute to AD neurodegeneration [6,36,37,38]. This work evaluated whether a novel therapeutic combination of ethanolic ASH and a water-solubilized CoQ10 (UQ, patented by Micillic Lda, Lisbon, Portugal) could provide diverse and successful protection against AD. Combining ASH and UQ therefore posits potential to influence several interconnected AD processes. Preclinical results discussed previously suggest that the co-administered nutraceutical agents may indeed target several core AD etiologies simultaneously [42,43,44,48].

Here we assessed Tonic’s effects against AD-associated pathological and functional decline in a stringent 5XFAD transgenic mouse model of AD [76]. We aimed to elaborate on previous work from our lab using the Tonic treatment [42,43,44,48] by incorporating a multi-dimensional experimental design to evaluate longitudinal efficacy of the therapy from 2.5 to 11 months of age via pre-mortem MRI, frailty, and cognitive memory tests, and post-mortem Golgi–Cox, Nissl, and immunofluorescence staining. In addition, we directly compared experimental outcomes in male and female mice to determine potential sex-dependent treatment effects.

### 4.1. Summary of Multi-Targeting Potential of Tonic-Mediated Protection

Across all analyses, Tonic treatment provided very beneficial results in both male and female mice. Altogether, our findings indicate that the effects of Tonic are vast, yet different depending on sex and experimental conditions. A summary of overall findings is reported in Table 5 and pre-mortem longitudinal results are summarized in Table 6.

#### 4.1.1. UQ and ASH Protective Effects on Brain Structure: Discussion of Golgi–Cox, Nissl, and MRI Results

Synaptic loss is considered one of the strongest structural correlates of cognitive decline in AD, often preceding substantial neuronal death and correlating more closely with disease severity than amyloid plaque burden itself [85,86,87,88,89,90]. Progressive dendritic atrophy reduces neuronal connectivity, disrupts hippocampal function, and impairs information processing required for learning and memory. In untreated 5XFAD mice, reductions in dendritic length, thickness, and spine maturity were consistent with the extensive dendritic remodeling previously described in both patients with AD and transgenic mouse models [90,143]. However, the intervention of our Tonic treatment substantially improved these structural measures, as seen in their dendritic morphology very closely mimicking wild-type animals.

Mature mushroom and branched spines represent stable excitatory synapses that support long-term potentiation and memory consolidation, whereas filopodia and long-thin spines are generally considered immature and highly dynamic structures involved in synapse formation [94]. The greater proportion of mature spines following treatment therefore suggests improved stabilization of functional synaptic connections [137]. These findings follow expected outcomes based on previous reports demonstrating that ASH promotes neurite outgrowth, dendritic branching, and synaptic plasticity through upregulation of neurotrophic signaling pathways [67,72,144], while CoQ10/UQ reduces oxidative injury that contributes to cytoskeletal instability and synaptic degeneration [134]. The combination of these complementary mechanisms likely provided a favourable environment to preserve dendritic integrity despite the genetic susceptibility of aggressive amyloid pathology.

Longitudinal MRI findings demonstrated preservation of structural integrity in several brain regions susceptible to AD. Hippocampal volumes in male and female 5XFAD mice treated with the Tonic showed adequate growth with age, similar to wild-type control animals. Meanwhile, 5XFAD untreated control mice showed slowed growth (females), or decline (males) in hippocampal volume with age. This result coincides with the expectation that un-intervened 5XFAD neuronal stress and degeneration would result in lower hippocampal volumes over time [22]. As for the white matter volumetric assessment, all groups of male mice showed little to no difference in these structures over time. This leads us to our surprisingly different finding in females: we found female untreated 5XFAD control mice exhibited obvious enlargement of corpus callosum, fimbria, and fornix volumes very early on in the disease progression, at about 3 months of age, and continued this trend throughout the remainder of the imaging timepoints. In this regard, the Tonic showed promise in neurostructural preservation since treated females maintained these volumes comparable to wild-type mice at each phase of imaging. These results went against our original thought that white matter atrophy would be the most obvious volumetric result [77], but our findings suggest the opposite of this, and perhaps enlarged white matter structures may be a prelude to degradation. Although tissue enlargement is often interpreted as preservation, increasing evidence suggests that transient increases in white matter volume may instead reflect inflammatory edema, reactive gliosis, or abnormal myelin remodeling rather than healthy tissue expansion [77,78,84]. This is because white matter is particularly susceptible to chronic neuroinflammation since oligodendrocytes are highly sensitive to inflammatory cytokines and oxidative stress [77,78,79,80]. Activated astrocytes and microglia release mediators capable of disrupting myelin maintenance, impairing oligodendrocyte survival, and ultimately compromising axonal conduction. The normalization of white matter volumes observed following Tonic treatment therefore likely reflects reduced inflammatory pathology rather than simple tissue loss or gain, supported by the accompanying reductions in inflammatory biomarkers, suggesting that structural preservation occurred secondary to suppression of the neuroinflammatory cascade.

Our structural results provide an important connection between the molecular and functional outcomes of our Tonic solution. Pre-mortem longitudinal MRI showed preservation of hippocampal volume in male and female Tonic-treated animals, and post-mortem Golgi–Cox analysis provided interesting evidence that the protection of brain tissue may in part relate to preservation of the hippocampal dendritic architecture. This also leads to an interesting note that perhaps the Tonic may be able to adapt to AD pathological decline through enhanced complexity of neuronal communicatory pathways to compensate for those that may be threatened by the disease.

Furthermore, Nissl staining was done to corroborate MRI findings. We wanted to see if these enlarged white matter structures in the female untreated 5XFAD control mice were because they simply had more cells in these regions. The results revealed no significant differences in cellular density within the corpus callosum of female mice across all group conditions, whereas male 5XFAD mice exhibited reduced cresyl violet staining in Tonic-treated and control 5XFAD mice, which correlate well with MRI results for this cohort. Because the corpus callosum contains relatively few neuronal cell bodies, Nissl staining in this region may therefore reflect primarily glial nuclei rather than neuronal survival [145]. Consequently, these findings likely indicate alterations in glial organization rather than direct neuronal loss.

#### 4.1.2. UQ and ASH Protective Effects on Molecular and Pathological Modulation: Discussion of Immunofluorescence Staining Results

Immunofluorescent analysis was done to look at the Tonic’s potential to protect against amyloid pathology, inflammation, and impaired autophagy-related processes. As expected, untreated 5XFAD control mice exhibited marked accumulation of APP and extracellular Aβ deposits compared with wild-type controls, which is typical of the aggressive amyloid pathology genetically characteristic of this transgenic model [76]. However, the Tonic substantially reduced amyloid burden in both sexes. These findings are consistent with previous reports demonstrating that ASH enhances amyloid clearance through upregulation of LRP1 to facilitate transport of Aβ from the brain into the peripheral circulation [64]. To compliment ASH, UQ has been shown to improve mitochondrial function and reduce oxidative stress, thereby decreasing cellular conditions that promote amyloidogenic APP processing [42,43,46,49,50,58]. The simultaneous reduction in APP expression and amyloid deposition observed in the present study suggests that the Tonic may influence both amyloid production and clearance simultaneously, ultimately providing a broader therapeutic effect than therapies that exclusively target existing extracellular plaques.

The reductions in amyloid pathology were accompanied by substantial suppression of neuroinflammatory markers. Activated microglia and astrocytes are increasingly recognized as central mediators of AD progression, by producing pro-inflammatory cytokines and ROS that exponentially increase neuronal injury and further impair protein clearance [13,14,15]. Consistent with this mechanism, 5XFAD control mice demonstrated significant increases in Iba-1 (activated microglia) in both males and females and IL-1β (pro-inflammatory cytokines) expression in males. Tonic treatment significantly reduced microglial markers, and inflammatory cytokine expression particularly in male mice, which suggests the treatment is beneficial toward dampening the persistent inflammatory environment characteristic of AD. The results here agree with previous reports demonstrating that withanolides inhibit NF-κB signaling while simultaneously activating antioxidant pathways mediated through Nrf2 [67,68,69]. Restoration of a less inflammatory microenvironment likely reduces secondary neuronal damage while also improving the efficiency of endogenous repair mechanisms [13,42,146].

Autophagy-related dysfunction represents another major contributor to AD, by allowing damaged organelles and misfolded proteins to accumulate within neurons [12]. Because oxidative stress, neuroinflammation, and impaired autophagy-related systems reinforce one another throughout disease progression, simultaneous improvement across these pathways likely contributes substantially to the overall neuroprotective effect observed. The wild-type-like expression of both LC3B and Beclin-1 markers observed in male mice treated with Tonic gives evidence of preserved autophagic activity. Functionally, LC3B participates in autophagosome formation, and Beclin-1 regulates autophagy initiation, and therefore both proteins are essential for intracellular protein turnover [147,148]. Previous studies have demonstrated that reduced expression of these proteins during AD progression results in impaired clearance of Aβ and dysfunctional mitochondria [149].

Looking at puncta morphology, LC3B immunoreactivity appeared more prominently punctate in Tonic-treated 5XFAD mice. Qualitatively, treated 5XFAD mice had more distinct and clustered structures compared with the diffuse fluorescent signal observed in untreated 5XFAD mice. The morphological assessment points toward enhanced formation or organization of autophagy-associated structures when Tonic treatment is administered [147,150]. Now, this makes sense mechanistically since UQ is known to support mitochondrial function and protect against oxidative stress, while ASH maintains cellular plasticity through neurotrophic signalling, ultimately providing a good environment for autophagic processing [42,43,44,48]. Altogether, the more organized punctate pattern in Tonic-treated mice is consistent with possible improvement of autophagy-related processes typically disrupted by AD pathology [147,150]. However, these markers should be interpreted cautiously. Beclin-1 and LC3B immunoreactivity provides evidence of changes in autophagy-associated pathways but does not, by itself, establish improved autophagic flux. However, qualitative assessment of LC3B-fluoresced autophagosomes did further support our prediction that autophagy procession is preserve in both male and female mice via more organized and discreet puncta morphology compared to the diffuse pattern seen in the untreated AD mice [147,150].

Moving forward to look at hippocampal α-GFAP immunoreactivity as a marker for astrocytic activity, male 5XFAD control mice exhibited substantially increased expression compared to wild-type and 5XFAD-Tonic-treated mice. The lower expression of astrocytic activity in Tonic-treated males likely reflects lessened inflammatory burden since IL1-β and Iba-1 markers show a similar trend. There were no significant differences in hippocampal astrocytic expression between female groups; however, female mice exhibited substantially greater astrocytic α-GFAP immunoreactivity throughout the corpus callosum than males. Nonetheless, the Tonic-treatment was able to bring astrocytic expression to levels equivalent with wild-type mice in both males and females, yet the differences were region-specific for each sex. Combined with the MRI and immunofluorescence data, the results suggest that the principal effects of the Tonic occur through modulation of glial function, inflammation, and white matter integrity.

#### 4.1.3. UQ and ASH Beneficial Effects on Functional Outcomes: Discussion of Frailty Index and Cognitive Memory Test Results

The longitudinal frailty assessment provided us with important insight into the Tonic’s influence on physiological disease progression. Frailty is increasingly recognized as a clinical feature of AD, and recent evidence demonstrates associations between physical decline, cognitive impairment, and accelerated disease progression [95,96,99]. Although frailty has traditionally been studied in aging research, its application in AD models provides a very relevant translational measure that touches on the multidimensional deterioration experienced by patients effecting their everyday quality of life [96,98]. In the present study, untreated 5XFAD control mice demonstrated exponentially increasing frailty across the experimental period, whereas Tonic-treated mice exhibited a slower progression of physical decline. While treatment did not completely prevent age-related increases in frailty, the statistical similarity to wild-type mice at the final testing timepoint suggests preservation of physiological resilience throughout disease progression that could lead to improved quality of life and functionality more similar to non-AD-suffering individuals.

From a mechanistic perspective, mitochondrial dysfunction contributes to reduced skeletal muscle function, impaired energy metabolism, oxidative stress, and systemic inflammation, all of which contribute to physical frailty [16,17,138]. Restoration of mitochondrial bioenergetics may therefore improve cellular energy production in both neural and peripheral tissues. ASH has likewise demonstrated antioxidant, anti-inflammatory, and adaptogenic properties that improve muscle function, physical endurance, and stress resilience in both clinical and preclinical studies [42,43,44,67,139,140]. The combination of these compounds therefore has the potential to influence both neurological and systemic aspects of AD progression by providing a broader therapeutic benefit than treatments directed solely toward cognition or extracellular protein clearance.

Now moving away from physical resilience, the Tonic treatment was also tested on its ability to preserve longitudinal cognitive resilience. Although habituation was generally stable across the testing period, looking at the individual habituation linear trajectories showed an interesting sex- and treatment-dependent pattern. The findings from our cognitive memory test suggest that Tonic treatment may preserve aspects of cognitive function in both males and females, but through different mechanisms. Tonic-treated 5XFAD mice generally demonstrated a more consistent habituation pattern than untreated 5XFAD control animals. After all, habituation is the simplest form of learning, so the protection of this process assumes that baseline sensitization and novel discrimination are also functional [151]. The more consistent habituation observed following Tonic treatment may also be due to simultaneous effects on anxiety-related or stress-responsive behavior. ASH has been associated with anxiolytic and stress-modulation [141], thus reduced anxiety could potentially alter the extent to which animals repeatedly explore a novel testing environment. However, the present habituation evaluation was not specifically intended to distinguish anxiety from learning, memory, or exploratory drive. In fact, it was actually designed to avoid these properties. Therefore, although the more consistent habituation trajectory in Tonic-treated females is consistent with a potential reduction in anxiety-related behavioral interference, this interpretation remains speculative and should be investigated using dedicated anxiety-related behavioral assays. The thought is nevertheless relevant when interpreting the subsequent NLR and NOR results.

During NLR and NOR working memory testing, total exploration time remained relatively unchanged between baseline and novelty exposure; however, analysis of individual object regions revealed selective increases in exploration of the relocated or novel object. This distinction is important to emphasize because spontaneous object recognition models rely on preferential allocation of exploration toward novelty rather than increased overall activity [125]. Healthy rodents seemed to naturally redistribute their exploration toward unfamiliar objects or locations while maintaining similar total exploration time. Consequently, the observed increase in exploration of novel stimuli without changes in total exploration is consistent with preserved recognition memory rather than hyperactivity. Thus, our findings support that Tonic maintained novelty-provoked exploratory behavior and that preservation of dendritic structure translated into functional improvements in hippocampal- and cortical-dependent memory processes [137]. To this point, the relationship between dendritic morphology and behavioral performance has been well established in both clinical and experimental studies of AD [85,88,89,90,137,143]. Hippocampal-dependent spatial memory relies on the integrity of dendritic arborization and stable excitatory synapses within the CA1 region and associated cortical networks [91,92]. Loss of dendritic complexity decreases synaptic integration and weakens long-term potentiation, ultimately impairing learning and memory. Although behavioral improvements were modest, this is consistent with previous studies showing that synaptic protection frequently precedes large improvements in cognitive performance, particularly in aggressive transgenic models such as 5XFAD where pathology develops rapidly [76,143,152].

### 4.2. Sex-Specific Differences in AD Pathology and the Tonic’s Adaptive Mechanisms of Protection

Perhaps the most unexpected, yet important finding across this study was that disease-associated therapeutic responses differed quite substantially between males and females. Nonetheless, both males and females showed benefits from the Tonic, but in very different ways, further emphasizing the great necessity of considering biological sex as a variable in preclinical investigations of novel therapeutics. Here, we will discuss the intricate ways in which our treatment provided sex-dependent benefits.

Firstly, premortem volumetric MRI observations showed early detection of white matter structure abnormalities, starting as early as 3 months of age. Specifically, female untreated 5XFAD mice demonstrated quite enlarged volumes of the corpus callosum, fimbria, and fornix, whereas the same regional volumes were generally unaffected among male groups. Furthermore, Nissl staining did not demonstrate increased cellular density within the female corpus callosum, and therefore the MRI-detected enlargement could not be explained by an overall increased number of cells. Therefore, we proceeded further to see if abnormal white matter volume may instead reflect a difference in the quality of the tissue. Moving forward, we employed several post-mortem histochemical tests to assess the astrocytic and neurostructural characteristics of the hippocampal and corpus callosum brain regions.

Immunofluorescence of astrocytes by assessment of α-GFAP immunoreactivity gave interesting sex- and treatment-specific outcomes. The female corpus callosum displayed much greater levels than males in general, but untreated 5XFAD mice showed reduced expression compared to Tonic-treated and wild-type cohorts. In contrast, male 5XFAD control mice demonstrated a more pronounced upregulation of hippocampal astrocytic expression that was reduced with intervention of the Tonic treatment. Now this observation makes sense considering males and females have different distributions of astrocytes within the brain and therefore may also rely on these astrocytic functions in different ways [153,154,155]. Recent studies indicate that female white matter contains a greater density of astrocytic processes and exhibits enhanced astrocyte reactivity within myelinated tracts compared to males [153,154,155]. This distinction is important when considering our results because increased GFAP immunoreactivity should not automatically be interpreted as harmful astrocytic activation. Astrocytes can take on different functional and morphological states depending on their environment and can contribute both inflammatory signaling and tissue maintenance or repair [80,156]. In white matter, specifically, astrocytes provide important support for oligodendrocytes and myelin integrity and contribute to myelin repair following inflammatory injury [153,154,155]. To add to this point, estrogen levels can heavily influence astrocyte morphology. For instance, during ovulation (high-estrogen phase of menstrual cycle for humans, equivalent to proestrus stage for mice), estrogen promotes rapid glial turnover in white matter, which can lead to increased α-GFAP expression in this region [154]. Thus, the higher astrocytic immunoreactivity observed in female white matter may not necessarily represent greater inflammatory reactivity and may instead reflect differences in astrocytic organization, baseline distribution, compensatory support, or repair of white matter [80,84,154,155]. The observation that Tonic-treated females exhibited white matter volumes and astrocytic responses more similar to wild-type animals suggests that treatment may have normalized the glial state toward a more supportive resting function.

From our assessment of neuronal architecture via Golgi–Cox staining, females showed the greatest improvement in dendritic spine density following treatment, whereas males exhibited more mature spine morphologies. Estrogen has well-established effects on dendritic spine formation and synaptic remodeling, and declining estrogen during aging has been correlated to accelerated cognitive decline [157,158]. However, during high-estrogen times of the female menstrual (human)/estrous (mouse) cycle, the hormone promotes rapid dendritic turnover [158,159]. Therefore, our observation that males had more mature dendrites and females had more dendritic spine density aligns well with other literature [158,159]. Perhaps female dendrites do not often reach mature levels due to frequent turnover and instead increase synaptic density to maintain neuronal communicatory pathways and synaptic resilience. Nonetheless, the Tonic solution provided evidence that it was able to promote more complex neuronal networks in males and females and shows the vast diversity of its therapeutic impact depending on sex-, disease-, and treatment-related conditions.

Moving forward, sex differences were also evident in autophagy-associated markers. Interestingly, restoration of LC3B and Beclin-1 expression in response to Tonic treatment was observed only in male mice, whereas female mice exhibited uniformly low expression across all groups. This sex difference has been observed in previous studies investigating autophagic activity and its regulation by sex hormones [160,161]. Estrogen signaling actually provides neuronal protection by suppressing Beclin-1 and LC3B and preventing the classical autophagy pathway to ultimately survive longer when neurons are in states of stress. Instead, estrogen prevents apoptosis of these stressed neurons by promoting alternative mechanisms of cellular homeostasis, such as forming lipid droplets, mobilizing fatty acids, and accumulating triglycerides [160,161]. Therefore, the observed lower basal expression of autophagy-related proteins in the females may be due to estrogen-mediated inhibition of these proteins or utilization of alternative protein clearance pathways [160,161]. To this point, the lower female immunoreactivity should not be interpreted as evidence of impaired autophagy. Instead, previous reports suggest that autophagy-associated proteins may be regulated differently between sexes, so their functional outcome in response to therapeutic intervention is near-incomparable and should be considered separately. That being said, the benefits of our Tonic were obvious in the male mice where we saw evidence that markers of autophagy initiation and autophagosome formation were normalized to wild-type levels. Therefore, this interesting difference brings us further insight into the flexibility of our treatment solution.

The overarching pattern observed throughout the current study suggests that the same treatment may work through very different cellular pathways in males and females. In reality, females represent > 66% of the AD population, and published reports have shown sex-specific differences in synaptic resilience, inflammatory signaling, mitochondrial function, and hormone-mediated neuroprotection [4,28,30,157,161]. These findings raise an important possibility: the therapeutic benefit of an intervention may be functionally equivalent between sexes even when the mechanisms producing that benefit differ substantially. In other words, sex may play a very important role in determining the pathological pathways that are most responsive to intervention and should be seriously considered when determining whether a treatment works. A truly effective treatment should produce similar overall protection and should be able to interact with different biological pathways in males and females depending on hormonal and disease-provoked cellular environments. Our Tonic treatment of UQ + ASH has shown promising evidence throughout this report that it may be able to do just that.

### 4.3. Limitations of the Present Findings and Future Directions

We would like to address several limitations here that should be considered when interpreting our outcomes. First, the 5XFAD model reproduces genetically induced amyloid pathology but does not fully recapitulate the full complexity of human AD [76]. Consequently, the therapeutic effects observed here should not be interpreted as evidence that Tonic will necessarily produce equivalent effects in patients. For instance, the 5XFAD mouse model is one of the most stringent mouse models of AD, containing five genetic mutations associated with early-onset AD [76]. This genetic condition would be extremely rare, if not non-existent in the human population. Moreover, the controlled lab-testing environment is not exactly comparable to the environmental, social, and genetic variability experienced in the real world. We did, however, put in substantial efforts to replicate the human experience to the best of our abilities with our little mice by providing group housing conditions and daily mouse-researcher interactions for socialization, as well as prioritizing non-anxiety provoking and non-invasive behavioral and MRI testing. Despite these efforts, it is absolutely necessary to properly test this treatment in clinical human models of AD at human equivalent dose (HED) [162]. Treatment dosing for mice is higher per kg of weight than what would be required for humans because of metabolic-rate differences between species. Therefore, before translating treatment directly to humans, its dosing should be normalized by using the following equation [162]:

HED mgkg=Animal no observed adverse effect levels (NOAL)mgkg×Weight animal  (kg)Weight human  (kg)1−0.67

With this calculation, our mouse NOAL is 8 mg/kg/day for UQ and 320 mg/kg/day for ASH, therefore translating to an average 60 kg human, equivalent doses would start at 0.61 mg/kg/day for UQ and 24.53 mg/kg/day for ASH. That being said, clinical trials are needed to confirm the translational efficacy and safety of this dose in humans in both preventative and therapeutic conditions.

Second, although the findings demonstrate clear sex-dependent patterns, the present study did not intend to investigate the molecular basis of these differences, and therefore we did not prepare to correlate our results with concurrent hormonal testing. Hormonal regulation, mitochondrial biology, inflammatory signaling, astrocytic function, and autophagy may all contribute, but the present experiments cannot determine the relative contribution of each mechanism. The proposed sex-specific mechanisms should therefore be considered as hypothesized. However, the current findings do raise a very important point, which deserves proper attention. Even so, our sex-dependent responses further contribute to the increasingly recognized influence of biological sex on AD progression [27,28,29,30,34,155,163].

Third, the post-mortem preparation of brain tissue limited the types of quantitative biochemical analyses that could be performed. Animals were transcardially perfused, and brains were subsequently post-fixed for histological and immunofluorescent analyses or prepared for Golgi–Cox staining. This procedure was necessary to preserve tissue architecture and enable the multidisciplinary histological analyses used throughout the study; however, fixation limits the suitability of these tissues for further quantitative biochemical techniques such as Western blotting. For this reason, the immunofluorescence findings provide valuable spatial information regarding the distribution and relative immunoreactivity of the selected markers but cannot independently establish quantitative protein amounts throughout the brain. A secondary linear mixed-effects analysis accounting for animal-specific variability further corroborated the reported findings overall (data not shown), supporting the observed post-mortem group effects and indicating sample-size sensitivity for female Aβ and spine measures in both sexes.

Finally, the current study did not directly investigate the complexity of the Tonic formulation itself. The objective of the present study was to evaluate the neuroprotective effects of the botanical extract as a whole rather than to identify the individual constituents responsible for its activity. Based on previous literature [42,43,44], when combined into the Tonic, the treatment demonstrated a broader and more balanced neuroprotective response across both AD and PD models, suggesting that the compounds may target complementary pathways of disease progression [48]. It is proposed that UQ primarily limits the upstream accumulation of mitochondrial and oxidative damage, and ASH may act downstream to promote neurotrophic support, glial homeostasis, synaptic remodeling, and neuronal recovery, thereby shifting the cellular environment from one dominated by oxidative and inflammatory injury toward one that is more permissive to repair and plasticity. However, it should be noted that the previous analyses were conducted using mostly qualitative immunohistochemical reports, and therefore, further quantitative investigation is necessary and encouraged to confirm the proposed therapeutic mechanisms of UQ + ASH. On that note, the current study did not include groups treated with UQ and ASH independently and, consequently, can only speculate on each components’ contributions based on what external literature has concluded. Moreover, the crude ASH extract tested in the previous reports may vary in phytochemical concentrations between batches and experiments, so more controlled pharmacokinetic/pharmacodynamic experimentation is also needed to determine the exact components responsible for the observed effects. The ASH component in the current study was administered as an ethanolic root extract containing multiple bioactive constituents, including withanolides and flavonoids, rather than as a single purified compound. Evidence suggests that the biological effects of ASH may in part be from interactions among multiple constituents, with some literature proposing synergistic effects among withanolides and potential modulation of component-specific toxicity within the broader phytochemical matrix [164]. Although the extract underwent phytochemical characterization, the concentration of individual bioactive compounds delivered to each animal and the distribution among tissues were not monitored. Treatment concentrations were calculated from average water consumption, and daily intake was monitored to improve dosing consistency; however, differences in individual water consumption, metabolism, absorption, and pharmacokinetics could have resulted in variability in actual exposure between animals. This limitation is particularly relevant because substantial variation in the phytochemical composition of ASH preparations has been reported, emphasizing the importance of chemical standardization and quality control [165,166].

Moving forward, we aim to build upon these preclinical findings through pharmacokinetic profiling and comprehensive chronic administration safety and toxicity assessments. UQ has received FDA-GRAS status, and preliminary histopathological evaluation conducted by a board-certified pathologist at the University of Guelph identified no overt lesions of toxicological significance in UQ-treated rats, including at doses up to 10-fold higher than the required dose [58]. Nevertheless, ASH and Tonic require additional toxicological evaluation to establish their safety profiles and support their further development. Future studies should include additional AD models, followed by appropriately powered and rigorously controlled translational studies, which are necessary before clinical efficacy can be inferred. In addition, further mechanistic studies should directly assess mitochondrial bioenergetics, oxidative stress, autophagic flux, synaptic function, and tau pathology. Moreover, our lab intends to evaluate pre- and post-mortem measurements in conjunction with measures of the female mouse estrous cycle to hopefully deduce the potential influence hormonal variability may have on the results.

## 5. Conclusions

The findings presented throughout this manuscript strongly support the concept that AD should be evaluated as a multi-factorial process in which amyloid pathology, inflammation, mitochondrial dysfunction, impaired proteostasis, glial dysregulation, neuronal degeneration, and systemic decline interact to produce progressive loss of function [7,8,9,10,11,12,13,14,15,16,17]. Here, we employed a multi-disciplinary experimental design using MRI, immunofluorescence, Golgi–Cox and Nissl staining, behavioral testing, and frailty assessment to investigate if UQ and ASH in a combination Tonic solution can diversely act on AD neurodegenerative progression.

The Tonic provided protection against a vast range of disease outcomes, yet this protection was not mechanistically identical between males and females. Female mice exhibited particularly prominent treatment responses involving white matter structure, astrocytic responses, and dendritic spine density, whereas males demonstrated stronger treatment-associated changes in inflammatory signaling, autophagy-associated markers, hippocampal astrocytic activity, and dendritic maturation. Functionally, both males and females responded with improved cognitive function through habituation and novelty-recognition measurements. Males treated with the Tonic additionally demonstrated long-term protection against accelerated AD physiological decline, indicating the Tonic provided no harmful side effects nor overt toxicity with long-term use. Thus, the present findings suggest that biological sex influences not only AD-related pathology but also the mechanisms through which a successful intervention may provide protection. Nonetheless, the results reported here are promising and strongly encourage the eventual evolution of UQ + ASH Tonic to human clinical trials.

## Figures and Tables

**Table 1:** Withanolide and flavonoid content of ethanolic ASH extract [46].

	Withanolides				Flavonoids
	Content (mg/mL)				Content (mg/100 mL)
	Withaferin A	12-Deoxy-Withastramonolide	Withanolide A	Withanolide B	Quercetin
**Assay 1**	13.0	3.7	5.3	1.8	Mean 1.63 ± 0.07
**Assay 2**	13.9	4.0	5.6	2.0	
**Assay 3**	13.8	3.8	5.6	1.7	
**Assay 4**	13.6	3.9	5.5	1.8	
**Mean ± standard deviation**	13.6 ± 0.4	3.8 ± 0.1	5.5 ± 0.1	1.9 ± 0.1	

**Table 2:** T2-weigthed 3-dimensional fast spin echo (TW 3D-FSE) magnetic resonance imaging (MRI) parameters for female and male mice.

T2W 3D-FSE Imaging Parameters	Female	Male
Typical field of view (mm)	20.16 × 20.16	18 × 18
Vertical resolution (mm)	0.16	0.14
Horizontal resolution (mm)	0.16	0.14
Number of slices	30	30
Slice thickness (mm)	0.6	0.6
Inter-slice gap	0	0
Number of excitations	1	1
Slice orientation	axial	axial
Repetition time (TR, ms)	3000	3000
Echo time (TE, ms)	111.92	113.35
Phase encoding	horizontal	horizontal
Number of phase encoding	128	128
ETL	16	16
Pixel bandwidth	158.7302	156.25
Total imaging time (sec)	750	750
Number of samples	126	128
Dwell time	50	50

Note: Minor modifications to field of view, image resolution, echo time, and acquisition settings were implemented between experimental cohorts to optimize image quality.

**Table 3:** Summary of immunofluorescent markers used in the current study and their intended purpose, grouped by general Alzheimer’s disease (AD)-related pathological process.

Pathological Process	Immunofluorescent Marker	Purpose
**Amyloid pathology**	β-Amyloid (Aβ)	To measure Aβ peptide load
	APP	To measure the amyloid precursor protein (APP), which is cleaved to generate Aβ, and its abnormal accumulation or distribution in AD pathology
**Neuroinflammation**	Interleukin-1β (IL-1β)	To assess pro-inflammatory signaling released by activated glial cells during neuroinflammation and AD progression.
	Ionized calcium-binding adapter molecule 1 (Iba1)	To evaluate microglial expression as an indicator of microglial involvement in AD-associated neuroinflammation.
	α-glial fibrillary acidic protein (α-GFAP)	To visualize the α-GFAP-containing astrocyte cytoskeleton, allowing assessment of astrocyte expression and morphology and identification of changes associated with reactive astrogliosis.
**Autophagy-Associated Processes**	Beclin-1	To assess initiation of autophagy, the cellular cleaning process, which is impaired in AD and can contribute to the accumulation of pathological proteins and debris.
	Microtubule-associated protein 1 light chain 3B (LC3B)	To measure autophagosome formation/maturation and autophagic activity involved with cellular clearance.

**Table 4:** Frailty index assessment criteria and definitions adapted from Whitehead et al. (2014) [96] and summary of how each was scored for the current project.

Criteria	Definition	How Criteria Was Scored
**Alopecia** **[100]**	Hair loss due to age-related balding and/or barbering (fur trimming)	Visual assessment of fur quality at time of test was used for assessment, in conjunction with monthly veterinarian reports. Alopecia was scored as extreme/full body coverage = 1, bad/large portion of body affected = 0.75, moderately bad/some of body affected = 0.5, some loss of fur in a small area = 0.25, absent = 0
**Loss of fur colour** **[100]**	Change in fur color from black to gray or brown	Photo of mouse’s belly and side profile was used for assessment. Here we looked at the amount of gray/lightened fur color on the belly and extending up their side, mainly around hips. Fur color loss was scored as complete = 1, full belly and some of side = 0.75, full belly but no side = 0.5, some belly = 0.25, no change = 0
**Dermatitis** **[101,102]**	Inflammation, overgrooming, barbering, or scratching causing skin erosion. Can result in open sores anywhere on the body	Visual assessment of skin conditions at time of test were used for assessment, in conjunction with monthly veterinarian reports. Dermatitis was scored as extreme/full body coverage = 1, bad/large portion of body affected = 0.75, moderately bad/some of body affected = 0.5, some skin flaking and irritation but no sores = 0.25, absent = 0
**Loss of whiskers** **[103,104]**	Loss of vibrissae (whiskers) due to aging and/or whisker trimming	Photo of mouses nose was used for assessment. Scored based on how much pink flesh is exposed around nose. Scored as completely absent whiskers with fully exposed skin = 1, few whiskers but mostly exposed skin = 0.75, moderately exposed skin = 0.5, minimally exposed skin, no skin exposed at all = 0
**Coat condition** **[101]**	Ruffled fur and/or matted fur. Ungroomed appearance. Coat does not look smooth, sleek, and shiny	Photo taken from under the mouse’s belly was used for assessment. Coat condition scored as extremely matted/tufted = 1, highly matted/tufted = 0.75, moderately matted/tufted 0.5, minimally matted/tufted, not at all matted/tufted = 0
**Tumours** **[101,102,105]**	Development of tumors or masses anywhere on the body	Monthly veterinarian reports were used for assessment. Tumors scored as extreme = 1, moderate = 0.5, absent = 0
**Distended abdomen** **[101]**	Enlarged abdomen. May be due to tumor growth, organ enlargement, or intraperitoneal fluid accumulation	Monthly veterinarian reports were used for assessment. Distended abdomen scored as extreme = 1, moderate = 0.5, absent = 0
**Kyphosis** **[100,101]**	Exaggerated outward curvature of the lower cervical/thoracic vertebral column. Hunched back or posture	Still photos from a video of mice walking up a moderately elevated notched board were used for assessment. Photos were compared to Yerger et al. (2022) [106] for scoring
**Tail stiffening** **[100]**	Tail appears stiff, even when animal is moving in the cage. Tail does not wrap freely when stroked	Full motion of tail and full wrap when stroked = 0, tip of tail is stiff and not able to wrap when stroked = 0.25, any additional stiff points along tail = +0.25 for a maximum score of 1
**Gait disorders** **[101]**	Lack of coordination in movement including hopping, wobbling, or uncoordinated gait. Wide stance. Circling or weakness	A video was taken of mice walking on a transparent floor. Evaluation of gait involved still photos of two full steps in active stride, when moving straight forward, all paws grounded, one foot completely weightbearing and one foot about to release to step forward. Mice were assessed using several criteria: (i) distance of weightbearing paw is to the same-side front paw, (ii) stepping foot alignment to the same-side front foot, and (iii) curve of line draw from nose point to tail base through the center of their body. Assessment was based on criteria from Liu, et al. (2015) [107].
**Tremor** **[101]**	Involuntary shaking at rest or during movement	Visual report at time of test was used for assessment. Tremor scored as extreme = 1, moderate = 0.5, absent = 0
**Forelimb grip strength** **[100]**	A decline in forelimb grip strength using the wire hang test	Recorded videos of the wire hang test were used for assessment. Mice were set onto thin metal rod 5 inches above the ground and timed by how long they can hold onto the wire. An average of 3 repeated trials per mouse was used to score their performance across timepoints. Timepoint 1 all mice scored 0, and at timepoint 2 and 3 their average time spent on the wire was subtracted from timepoint 1. Mice are scaled between subject by their difference from timepoint 1 to 2 or 3 from most positive to most negative. Highest 33% of values = 0, mid 33% of values = 0.5, lowest 33% values = 1
**Body condition score** **[100,101]**	Visual signs of muscle wasting or obesity based on the amount of flesh covering bony protuberances	Photo used for assessment and compared against veterinarian reference guide. Scaled from obese/emaciated = 1, over/under conditioned, = 0.5, well-conditioned = 0
**Vestibular disturbance** **[102]**	Disruption in the ability to perceive motion and gravity. Reflected in problems with balance, orientation, and acceleration	Rocked holding chamber from side-to- side and if mouse toppled completely over = disturbance (1), toppled over but recovered with some ease = 0.75, slightly lost balance but easily recovered or if 1–2 paws remained grounded = 0.5, slightly slipped but maintained balanced = 0.25, maintained full balance with all paws remaining in place = 0
**Hearing loss** **[102,108]**	Failure to respond to sudden sound (e.g., finger snap or clicker) indicative of hearing loss or impairment	Snapped fingers behind head/out of eyeline of mouse. Assessed ear movement in response to sound. Both ears respond = 0, both ears respond but at closer distance = 0.25, one ear responds = 0.5, one ear responds at closer distance = 0.75, no response = 1
**Cataracts** **[102,109,110]**	Clouding of the lens of the eye. An opaque spot in the center of the eye	Photos used for assessment and compared to photos referenced in the definition to the left.
**Corneal opacity** **[101,111]**	Development of white spots on the cornea. Cloudy cornea	Photos used for assessment and compared to Chen et al. (2018) [112] for scoring
**Eye discharge/swelling** **[101]**	Eyes are swollen or bulging (exopthalmia). They may exhibit abnormal secretions and/or crusting	Photos used for assessment. Eye discharge or swelling is scored as extreme = 1, moderate = 0.5, absent = 0
**Microphthalmia** **[101,102]**	Eyes are small and/or sunken. May involve one or both eyes	Photos used for assessment and compared to Blizzard et al. (2021) [113] for scoring
**Vision loss** **[102,114]**	Vision loss, indicated by failure to reach toward the ground when lowered by the tail	Scored by if they reach out with both front paws > 1 cm above ground = no disturbance (0), one hand extended (0.25), reached both hands out but <1 cm above the ground (0.5), reached one hand out < 1 cm above the ground (0.75), did not reach out hands at all and hit face on ground (1)
**Menace reflex** **[115]**	Rapid eye blink and closure of the palpebral fissure in response to a nontactile visual threat to the eye. Measures the integrity of the entire visual pathway including cortical components	Gently tapped face near eye to assess reflex eye blink response. Response = 0, no response = 1
**Nasal discharge** **[101]**	Signs of abnormal discharge from the nares	Photos used for assessment. Nose discharge is extreme = 1, moderate = 0.5, absent = 0
**Malocclusions** **[101]**	Incisor teeth are uneven or overgrown. Top teeth grow back into the roof of the mouth or bottom teeth are long and easily seen	Monthly veterinarian reports used for assessment. Malocclusion scored as extreme = 1, moderate = 0.5, absent = 0
**Rectal prolapse** **[101,116]**	Protrusion of the rectum just below the tail	Photos used for assessment. Prolapse scored as extreme = 1, moderate = 0.5, absent = 0
**Vaginal/uterine/penile prolapse** **[101,116,117,118]**	Vagina or uterus protrudes through the vagina and vulva. Penis cannot reenter the penile sheath	Photos used for assessment. Prolapse scored as extreme = 1, moderate = 0.5, absent = 0
**Diarrhoea** **[101]**	Feces on the walls of the home cage. Bedding adheres to feces in cage. Feces, blood, or bedding around the rectum	Visual assessment at time of test used for assessment. Diarrhea scored as extreme = 1, moderate = 0.5, absent = 0
**Breathing rate/depth** **[101,119]**	Difficulty breathing (dyspnea), pulmonary congestion (rales), and/or rapid breathing (tachypnea)	Monthly veterinarian reports used for assessment. Breathing scored as extreme = 1, moderate = 0.5, absent = 0
**Mouse Grimace Scale** **[120,121]**	Measure of pain/discomfort based on facial expression. Assessment of five facial features: orbital tightening, nose bulge, cheek bulge, ear position (drawn back), or whisker change (either backward or forward)	Photos used for assessment. Each facial feature described in the definition that is present is +0.25 for a maximum score of 1
**Piloerection** **[103]**	Involuntary bristling of the fur due to sympathetic nervous system activation	Photos used for assessment and scored as extreme = 1, moderately high = 0.75, moderate = 0.5, moderately low = 0.25, absent = 0
**Temperature score** **[103,122]**	Body temperature at time of test	Temperature taken by infrared thermometer positioned under belly. Between 34 °C and 38 °C is normal (0), any deviations scored as 1 [123]
**Body weight score** **[103,122,124]**	Body weight at time of test	Body weight scaled from largest to smallest weight. Largest 33% of values = 0, mid 33% of values = 0.5, and lowest 33% of values values = 1

**Table 5:** Summary of all results from our 45 male and female 5XFAD and wild-type mice along with proposed mechanisms of therapeutic protection against AD pathology, sourced from previous literature.

Biological Process	Pathological Marker/Experimental Test	Experimental Outcome	Proposed Therapeutic Mechanism from External Literature	Sex-Specific Observation
**Amyloid Pathology**	Amyloid-β (Aβ) and amyloid precursor protein (APP) immunofluorescence	Marked accumulation in untreated 5XFAD; Tonic treatment significantly reduced Aβ and APP levels, restoring expression to levels comparable to wild-type controls.	Ashwagandha (ASH) may promote Aβ clearance through pathways involving low-density lipoprotein receptor-related protein (LRP1)-mediated transport [64]. Ubisol-Q10 (UQ) may contribute to reduced amyloid pathology through improved mitochondrial function and reduced amyloidogenic processing [131].	Significant reduction observed in males and females, despite females showing higher basal levels.
**Neuroinflammation**	Ionized calcium-binding adapter molecule 1 (Iba-1) and interleukin-1β (IL-1β) immunofluorescence	Significant elevation of microglial markers and pro-inflammatory cytokines in 5XFAD controls; Tonic treatment substantially reduced expression.	ASH withanolides have been reported to modulate nuclear factor kappa-light-chain-enhancer of activated B cells (NF-κB) and nuclear factor erythroid 2-related factor 2 (Nrf2)-related inflammatory and antioxidant pathways [68,69]. UQ has been reported to reduce Aβ-induced microglial activation [132].	Suppression of inflammatory signaling was more pronounced in Tonic-treated males; treated females reached wild-type levels for Iba-1.
**Autophagy**	Microtubule-associated protein 1 light chain 3B (LC3B) and Beclin-1 immunofluorescence	Substantially reduced expression and disrupted puncta morphology in 5XFAD; Tonic treatment improved immunoreactivity and autophagosome formation.	UQ may support mitochondrial bioenergetics and autophagy-related processes [47,74]. ASH may contribute support through maintenance of neurotrophic and cellular homeostatic signaling [43,66].	Wild-type-like normalization of marker expression was specifically demonstrated in males; females showed uniformly low expression across all groups.
**Neuronal Morphology and Dendritic/Synaptic Health**	Dendritic length, thickness, spine density, and maturity by Golgi–Cox staining	Reductions in dendritic architecture and spine maturity in male and female 5XFAD control mice; Tonic treatment improved dendritic length, thickness, spine density, and maturity.	ASH has been reported to promote neurite outgrowth, branching, and synaptic plasticity [67,72]. UQ may reduce oxidative injury and associated cytoskeletal instability [133,134].	Females showed the greatest improvement in spine density; males showed a more pronounced increase in mature spine morphology.
**Astrocytic Activity**	α-glial fibrillary acidic protein (α-GFAP) immunofluorescence in hippocampus and corpus callosum	Elevated hippocampal α-GFAP in 5XFAD control males; reduced corpus callosum expression in untreated 5XFAD control females. Tonic treatment restored expression to wild-type levels in both regions.	UQ may modulate glial responses through stabilization of mitochondrial permeability transition pore (mPTP) and Bcl-2-associated X protein (BAX)-mediated apoptosis [63,135]. ASH may support neuroprotective astrocytic signaling and brain-derived neurotrophic factor (BDNF)-related pathways [22,43].	Females had pronounced normalization in white matter; Males had pronounced reduction in hippocampal astrocytic response.
**Brain Structure**	Hippocampal and white matter volume (corpus callosum, fimbria, fornix) by magnetic resonance imaging (MRI)	Untreated 5XFAD showed hippocampal decline and females displayed abnormal white matter enlargement; Tonic treatment preserved volumes.	The preservation of brain structure, both white and gray matter, may be associated with modulation of glial activity and reduction of neuroinflammatory processes, possibly tempering inflammatory tissue degradation [72,80,133,135].	Normalization of abnormal white matter enlargement was particularly prominent in females treated with Tonic; untreated males showed more significant differences in hippocampal volume over time.
**Cognitive/Behavioral**	Habituation, Novel Location Recognition (NLR), and Novel Object Recognition (NOR) test for working spatial and non-spatial memory assessment	Impaired novelty-recognition and habituation in untreated 5XFAD mice; Tonic treatment preserved habituation and recognition memory.	Preservation of cognitive performance may be related to maintenance of dendritic architecture, mitochondrial energy production/antioxidant defense, and functional synaptic stability in hippocampal and cortical networks [72,74,132,136,137].	Untreated females showed more evidence of spatial memory decline; untreated males showed more variation in non-spatial novelty response.
**Physiological Resilience and Well-Being**	Mouse frailty index [96]	5XFAD mice showed exponentially increasing physiological decline; Tonic treatment slowed frailty progression to wild-type equivalent levels.	UQ may support mitochondrial bioenergetics [50,138], while ASH may contribute anti-stress, antioxidant, and anti-inflammatory activity [139,140,141] in peripheral tissues that supports physiological resilience [142].	Test was only conducted in male mice.

**Table 6:** Summary of pre-mortem longitudinal results from our 45 male and female 5XFAD/control, 5XFAD/Tonic, and wild-type/control mice by experiment, sex, and age at time of evaluation.

Experiment	Sex	3–4 Months	6–7 Months	9–10 Months
MRI: Brain structure	Female	All groups: hippocampal and white matter volumes n.s., but 5XFAD/control white matter appears ↑	All groups: hippocampal volume n.s.; 5XFAD/control: ↑ white matter volume and Tonic ≅ wild-type	Tonic: ↓ white matter volumes relative to wild-type and 5XFAD/control still appears enlarged but n.s.; 5XFAD/control: ↓ hippocampal volume and Tonic ≅ wild-type
	Male	5XFAD/control: ↓ hippocampal volume and Tonic ≅ wild-type; all groups: white matter structures n.s.	5XFAD/control: ↓ hippocampal volume; all groups: white matter structures n.s.	5XFAD/control: ↓ hippocampal volume and Tonic ≅ wild-type; all groups: white matter structures n.s. except Tonic corpus callosum ↓ relative to wild-type
Frailty index	Female	Only conducted in males		
	Male	5XFAD/control ↑ frailty relative to wild-type	5XFAD/control ↑ frailty relative to wild-type	5XFAD/control ↑ frailty relative to wild-type and Tonic mice
Habituation	Female	5XFAD/control: ↑ on Day 2 (inconsistent habituation); all groups: n.s. exploratory response on test day	5XFAD/control: ↑ exploration on Day 3 habituation and Day 6 test (inconsistent habituation); Tonic and wild-type: ↑ exploration on test day	5XFAD/control: exploratory response n.s. on test day; Tonic and wild-type: ↑ exploration on test day
	Male	All groups: ↓ exploratory slope over habituation days; 5XFAD/control: ↑ Day 4 exploration (inconsistent habituation)	5XFAD/control: ↑ exploratory slope; Tonic and wild-type: ↓ exploratory slope over habituation days	5XFAD/control: ↑ exploratory slope; Tonic and wild-type: ↓ exploratory slope over habituation days
Spatial Memory (NLR)	Female	All groups novelty response n.s.	All groups: novelty response n.s.	Tonic and wild-type: ↑ novelty response; 5XFAD/control: n.s.
	Male	All groups: novelty response n.s.	All groups: novelty response n.s.	All groups: ↑ novelty response
Non-Spatial Memory (NOR)	Female	All groups: novelty response n.s.	5XFAD/control: ↑ novelty response; Tonic and wild-type: n.s.	All groups: novelty response n.s.
	Male	All groups: novelty response n.s.	5XFAD/control and wild-type: ↑ novelty response; Tonic: n.s.	Wild-type: ↑ novelty response; 5XFAD/control: n.s. and no increase; Tonic: ↑ exploration, but n.s.

Note: ↑ indicates an increase or improvement relative to the relevant comparison, ↓ indicates a decrease or reduction, “n.s.” = not significant, “MRI” = magnetic resonance imaging, “NLR” = Novel Location Recognition, “NOR” = Novel Object Recognition.

## Data Availability

The data supporting the reports within the current study are available upon request. Raw data is not publicly available due to privacy and ethical reasons.

## Supplementary Materials

The following supporting information can be downloaded at: https://www.mdpi.com/article/10.3390/nu18193270/s1, Table S1: (**A**) Two-way repeated-measures analysis of variance (ANOVA) summary of magnetic resonance imaging (MRI) brain region segmentation results. (**B**) Bonferroni-adjusted multiple comparisons of magnetic resonance imaging (MRI) brain region segmentation results.; Table S2: (**A**) One-way analysis of variance (ANOVA) summary of Nissl stain results in corpus callosum. (**B**) Bonferroni-corrected pairwise comparisons of Nissl stain results in corpus callosum.; Figure S1: Representative Nissl-stained images (20× and 40× magnification).; Table S3: (**A**) One-way analysis of variance (ANOVA) summary of immunofluorescence results. (**B**) Bonferroni-corrected pairwise comparisons of immunofluorescent results.; Figure S2: (**A**) Qualitative representative images of hippocampal amyloid-β (Aβ) immunoreactivity in the dentate gyrus and CA1 (Cornu Ammonis area 1) region of the hippocampus of male and female wild-type/control, 5XFAD/control, and 5XFAD/Tonic-treated mice. Representative images were taken at 20× magnification and scale bar = 100 μm. (**B**) Qualitative representative images of hippocampal amyloid precursor protein (APP) immunoreactivity in the dentate gyrus and CA1 (Cornu Ammonis area 1) region of the hippocampus of male and female wild-type/control, 5XFAD/control, and 5XFAD/Tonic-treated mice. Representative images were taken at 20× magnification and scale bar = 100 μm. (**C**) Qualitative representative images of hippocampal ionized calcium-binding adapter molecule 1 (Iba-1) immunoreactivity in the dentate gyrus and CA1 (Cornu Ammonis area 1) region of the hippocampus of male and female wild-type/control, 5XFAD/control, and 5XFAD/Tonic-treated mice. Representative images were taken at 20× magnification and scale bar = 100 μm. (**D**) Qualitative representative images of hippocampal interleukin-1β (IL-1β) immunoreactivity in the dentate gyrus and CA1 (Cornu Ammonis area 1) region of the hippocampus of male and female wild-type/control, 5XFAD/control, and 5XFAD/Tonic-treated mice. Representative images were taken at 20× magnification and scale bar = 100 μm. (**E**) Qualitative representative images of hippocampal Beclin-1 immunoreactivity in the dentate gyrus and CA1 (Cornu Ammonis area 1) region of the hippocampus of male and female wild-type/control, 5XFAD/control, and 5XFAD/Tonic-treated mice. Representative images were taken at 20× magnification and scale bar = 100 μm.; Table S4: (**A**) One-way analysis of variance (ANOVA) summary of Golgi–Cox staining results. (**B**) Bonferroni-corrected pairwise comparisons of Golgi–Cox staining results.; Table S5: (**A**) Two-way repeated-measures analysis of variance (ANOVA) summary of frailty index results. (**B**) Bonferroni-adjusted multiple comparisons of frailty index results. (**C**) Frailty index criteria and representative photos of how the frailty index was implemented in the current study. The table follows three individual mice through each criteria to show the physiological variation in aging outcomes from the highest scored mouse, a modestly scored mouse, and the lowest scored mouse at 9 months of age. Examples of the highest scored mouse per criteria is also provided if the other three mice do not exemplify this score. Refer to Table 4 for definitions of criteria and explanation of scoring.; Table S6: (**A**) Paired sample *t*-test summary for total object area exploration on habituation and testing days. (**B**) Spatial (Novel Location Recognition) and non-spatial (Novel Object Recognition) memory test summary of paired sample *t*-test comparing exploration of individual object areas on habituation (Day 6 Trials 1 + 2) vs. test (Day 6 Trials 3 + 4) conditions.

## Abbreviations

The following abbreviations are used in this manuscript:

ATP	Adenosine triphosphate
AD	Alzheimer’s disease
PD	Parkinson’s disease
NHPs	Natural health products
Aβ	Amyloid-β
ROS	Reactive oxygen species
NMDA	N-methyl-D-aspartate
CoQ10	Coenzyme Q10
ETC	Electron transport chain
UQ	Ubisol-Q10
PTS	Polyoxyethanyl-α-tocopheryl sebacate
ASH	Ashwagandha
NFTs	Neurofibrillary tangles
AChE	Acetylcholinesterase
LRP1	Low-density lipoprotein receptor-related protein
MRI	Magnetic resonance imaging
T2W	T2-weighted
3D	3-dimensional
FSE	Fast Spin Echo
CSF	Cerebrospinal fluid
ANOVA	Analysis of variance
SEM	Standard error of the mean
APP	Amyloid precursor protein
IL-1β	Interleukin-1β
Iba1	Ionized calcium-binding adapter molecule 1
α-GFAP	α-glial fibrillary acidic protein
LC3B	Microtubule-associated protein 1 light chain 3B
FAD	Familial Alzheimer’s disease
SAD	Sporadic Alzheimer’s disease
NLR	Novel Location Recognition
NOR	Novel Object Recognition
HED	Human equivalent dose

## References

[1] Neațu M., Covaliu A., Ioniță I., Jugurt A., Davidescu E.I., Popescu B.O. (2023). Monoclonal Antibody Therapy in Alzheimer’s Disease. Pharmaceutics.

[2] Fasoro O.S., Anyanwu F.C., Jayeola A.O., Okobi O.E., Osaigbovo J.O., Onojedje A.C., Balogun R.O., Abah O.C., Okoro A., Okeke-Chikelu C. (2025). Incidence of Dementia in Canada: A National Trend Analysis of Newly Diagnosed Cases. Cureus.

[3] Khoury R., Ghossoub E. (2019). Diagnostic biomarkers of Alzheimer’s disease: A state-of-the-art review. Biomark. Neuropsychiatry.

[4] (2023). 2023 Alzheimer’s disease facts and figures. Alzheimers Dement..

[5] Girard S.D., Jacquet M., Baranger K., Migliorati M., Escoffier G., Bernard A., Khrestchatisky M., Féron F., Rivera S., Roman F.S. (2014). Onset of hippocampus-dependent memory impairments in 5XFAD transgenic mouse model of Alzheimer’s disease. Hippocampus.

[6] Pei Y., Davies J., Zhang M., Zhang H.T. (2020). The Role of Synaptic Dysfunction in Alzheimer’s Disease. J. Alzheimer’s Dis..

[7] Selkoe D.J. (1994). Alzheimer’s Disease: A Central Role for Amyloid. J. Neuropathol. Exp. Neurol..

[8] Sheng M., Sabatini B.L., Sudhof T.C. (2012). Synapses and Alzheimer’s Disease. Cold Spring Harb. Perspect. Biol..

[9] Ballatore C., Lee V.M.-Y., Trojanowski J.Q. (2007). Tau-mediated neurodegeneration in Alzheimer’s disease and related disorders. Nat. Rev. Neurosci..

[10] Marcus J.N., Schachter J. (2011). Targeting Post-translational Modifications on Tau as a Therapeutic Strategy for Alzheimer’s Disease. J. Neurogenet..

[11] Medeiros R., Baglietto-Vargas D., LaFerla F.M. (2011). The Role of Tau in Alzheimer’s Disease and Related Disorders. CNS Neurosci. Ther..

[12] Li Q., Liu Y., Sun M. (2017). Autophagy and Alzheimer’s Disease. Cell. Mol. Neurobiol..

[13] Glass C.K., Saijo K., Winner B., Marchetto M.C., Gage F.H. (2010). Mechanisms Underlying Inflammation in Neurodegeneration. Cell.

[14] Alkhalifa A.E., Al Mokhlf A., Ali H., Al-Ghraiybah N.F., Syropoulou V. (2025). Anti-Amyloid Monoclonal Antibodies for Alzheimer’s Disease: Evidence, ARIA Risk, and Precision Patient Selection. J. Pers. Med..

[15] Wu Y., Eisel U.L.M. (2023). Microglia-Astrocyte Communication in Alzheimer’s Disease. J. Alzheimers Dis..

[16] Rojas-Gutierrez E., Muñoz-Arenas G., Treviño S., Espinosa B., Chavez R., Rojas K., Flores G., Díaz A., Guevara J. (2017). Alzheimer’s disease and metabolic syndrome: A link from oxidative stress and inflammation to neurodegeneration. Synapse.

[17] Brookes P.S., Yoon Y., Robotham J.L., Anders M.W., Sheu S.-S. (2004). Calcium, ATP, and ROS: A mitochondrial love-hate triangle. Am. J. Physiol. Cell Physiol..

[18] Braak H., Thal D.R., Ghebremedhin E., Del Tredici K. (2011). Stages of the Pathologic Process in Alzheimer Disease: Age Categories from 1 to 100 Years. J. Neuropathol. Exp. Neurol..

[19] Rahman M.A., Rahman M.S., Uddin M.J., Mamum-Or-Rashid A.N.M., Pang M.-G., Rhim H. (2020). Emerging risk of environmental factors: Insight mechanisms of Alzheimer’s diseases. Environ. Sci. Pollut. Res..

[20] Braak H., Braak E. (1991). Neuropathological stageing of Alzheimer-related changes. Acta Neuropathol..

[21] Stefanoska K., Gajwani M., Tan A.R.P., Ahel H.I., Asih P.R., Volkerling A., Poljak A., Ittner A. (2022). Alzheimer’s disease: Ablating single master site abolishes tau hyperphosphorylation. Sci. Adv..

[22] Kuboyama T., Tohda C., Komatsu K. (2014). Effects of Ashwagandha (Roots of *Withania somnifera*) on Neurodegenerative Diseases. Biol. Pharm. Bull..

[23] Lipton P.A., Eichenbaum H. (2008). Complementary roles of hippocampus and medial entorhinal cortex in episodic memory. Neural Plast..

[24] Frisoni G.B., Fox N.C., Jack C.R., Scheltens P., Thompson P.M. (2010). The clinical use of structural MRI in Alzheimer disease. Nat. Rev. Neurol..

[25] Mahrous R., Ghareeb D.A., Fathy H.M., EL-Khair R.M., Omar A.A. (2017). Protective Effect of Egyptian *Withania somnifera* Against Alzeheimer’s. Med. Aromat. Plants.

[26] Yang T., Bavley R.L., Fomalont K., Blomstrom K.J., Mitz A.R., Turchi J., Rudebeck P.H., Murray E.A. (2014). Contributions of the hippocampus and entorhinal cortex to rapid visuomotor learning in rhesus monkeys. Hippocampus.

[27] Lopez-Lee C., Torres E.R.S., Carling G., Gan L. (2024). Mechanisms of sex differences in Alzheimer’s disease. Neuron.

[28] Waters A., Laitner M.H. (2021). Biological sex differences in Alzheimer’s preclinical research: A call to action. Alzheimers Dement. Transl. Res. Clin. Interv..

[29] Laws K.R., Irvine K., Gale T.M. (2016). Sex differences in cognitive impairment in Alzheimer’s disease. World J. Psychiatry.

[30] Lutshumba J., Wilcock D.M., Monson N.L., Stowe A.M. (2023). Sex-based differences in effector cells of the adaptive immune system during Alzheimer’s disease and related dementias. Neurobiol. Dis..

[31] Manasa M., Bethanagere Ramesha G., Krishna K.L., Ambika M.S., Nadig A.P.R., Pathak S., Jalawadi P. (2026). Harnessing estrogen’s neuroprotective potential in mitigating Alzheimer’s pathologies in postmenopausal women: A review. Brain Res..

[32] Mishra P., Esfahani E.K., Fernyhough P., Albensi B.C. (2025). Estradiol Prevents Amyloid Beta-Induced Mitochondrial Dysfunction and Neurotoxicity in Alzheimer’s Disease via AMPK-Dependent Suppression of NF-κB Signaling. Int. J. Mol. Sci..

[33] Simpkins J.W., Yi K.D., Yang S.-H., Dykens J.A. (2010). Mitochondrial mechanisms of estrogen neuroprotection. Biochim. Biophys. Acta.

[34] Mazure C.M., Swendsen J. (2016). Sex differences in Alzheimer’s disease and other dementias. Lancet Neurol..

[35] Chopade P., Chopade N., Zhao Z., Mitragotri S., Liao R., Chandran Suja V. (2023). Alzheimer’s and Parkinson’s disease therapies in the clinic. Bioeng. Transl. Med..

[36] Peng Y., Jin H., Xue Y., Chen Q., Yao S., Du M., Liu S. (2023). Current and future therapeutic strategies for Alzheimer’s disease: An overview of drug development bottlenecks. Front. Aging Neurosci..

[37] Zemek F., Drtinova L., Nepovimova E., Sepsova V., Korabecny J., Klimes J., Kuca K. (2014). Outcomes of Alzheimer’s disease therapy with acetylcholinesterase inhibitors and memantine. Expert Opin. Drug Saf..

[38] Broadstock M., Ballard C., Corbett A. (2014). Latest treatment options for Alzheimer’s disease, Parkinson’s disease dementia and dementia with Lewy bodies. Expert Opin. Pharmacother..

[39] Graham W.V., Bonito-Oliva A., Sakmar T.P. (2017). Update on Alzheimer’s Disease Therapy and Prevention Strategies. Annu. Rev. Med..

[40] Van Bulck M., Sierra-Magro A., Alarcon-Gil J., Perez-Castillo A., Morales-Garcia J.A. (2019). Novel Approaches for the Treatment of Alzheimer’s and Parkinson’s Disease. Int. J. Mol. Sci..

[41] Scarpini E., Schelterns P., Feldman H. (2003). Treatment of Alzheimer’s disease; current status and new perspectives. Lancet Neurol..

[42] Vegh C., Walach G., Dube K., Malakoti-Negad N.J., Wear D., Jayawardena H., Patel M., Polsinelli A., Eren S., Cohen J. (2026). Anti-Inflammatory and Neuroprotective Effects of a Novel Water-Soluble Formulation of Coenzyme-Q10 and Ashwagandha Root Extract in a Paraquat-Induced Rat Model of Parkinson’s Disease. Biomed. Hub.

[43] Vegh C., Walach G., Dube K., Dobson B., Talukdar R., Wear D., Jayawardena H., Dufault K., Culmone L., Srikantha S. (2025). Investigation into Efficacy and Mechanisms of Neuroprotection of Ashwagandha Root Extract and Water-Soluble Coenzyme Q10 in a Transgenic Mouse Model of Alzheimer’s Disease. Nutrients.

[44] Vegh C., Wear D., Okaj I., Huggard R., Culmone L., Eren S., Cohen J., Rishi A.K., Pandey S. (2021). Combined Ubisol-Q10 and Ashwagandha Root Extract Target Multiple Biochemical Mechanisms and Reduces Neurodegeneration in a Paraquat-Induced Rat Model of Parkinson’s Disease. Antioxidants.

[45] Muthukumaran K., Kanwar A., Vegh C., Marginean A., Elliott A., Guilbeault N., Badour A., Sikorska M., Cohen J., Pandey S. (2017). Ubisol-Q10 (a Nanomicellar Water-Soluble Formulation of CoQ_10_) Treatment Inhibits Alzheimer-Type Behavioral and Pathological Symptoms in a Double Transgenic Mouse (TgAPEswe, PSEN1dE9) Model of Alzheimer’s Disease. J. Alzheimers Dis..

[46] Wear D., Vegh C., Sandhu J.K., Sikorska M., Cohen J., Pandey S. (2021). Ubisol-Q10, a Nanomicellar and Water-Dispersible Formulation of Coenzyme-Q10 as a Potential Treatment for Alzheimer’s and Parkinson’s Disease. Antioxidants.

[47] Vegh C., Pupulin S., Wear D., Culmone L., Huggard R., Ma D., Pandey S. (2019). Resumption of Autophagy by Ubisol-Q_10_ in Presenilin-1 Mutated Fibroblasts and Transgenic AD Mice: Implications for Inhibition of Senescence and Neuroprotection. Oxidative Med. Cell. Longev..

[48] Dube K., Stoinescu A., Pandey S. (2026). Curcumin, Coenzyme-Q10, and Bioactive Compounds in Ashwagandha Extract: Multi-Targeting Potential of Co-Administered Natural Health Compounds as Therapeutic and Preventative Interventions in Alzheimer’s and Parkinson’s Disease Models. Nutrients.

[49] Flint Beal M. (2002). Coenzyme Q_10_ as a Possible Treatment for Neurodegenerative Diseases. Free Radic. Res..

[50] Mancuso M., Orsucci D., Calsolaro V., Choub A., Siciliano G. (2009). Coenzyme Q10 and Neurological Diseases. Pharmaceuticals.

[51] Bhagavan H.N., Chopra R.K. (2007). Plasma coenzyme Q10 response to oral ingestion of coenzyme Q10 formulations. Mitochondrion.

[52] Beal M.F., Oakes D., Shoulson I., Henchcliffe C., Galpern W.R., Haas R., Juncos J.L., Nutt J.G., Voss T.S., Ravina B. (2014). A Randomized Clinical Trial of High-Dosage Coenzyme Q10 in Early Parkinson Disease. JAMA Neurol..

[53] Galasko D.R., Peskind E., Clark C.M., Quinn J.F., Ringman J.M., Jicha G.A., Cotman C., Cottrell B., Montine T.J., Thomas R.G. (2012). Antioxidants for Alzheimer Disease: A Randomized Clinical Trial with Cerebrospinal Fluid Biomarker Measures. Arch. Neurol..

[54] Maciejewska-Stupska K., Czarnecka K., Szymański P. (2024). Bioavailability enhancement of coenzyme Q_10_: An update of novel approaches. Arch. Pharm..

[55] Mantle D., Dybring A. (2020). Bioavailability of Coenzyme Q10: An Overview of the Absorption Process and Subsequent Metabolism. Antioxidants.

[56] Zaki N.M. (2016). Strategies for oral delivery and mitochondrial targeting of CoQ_10_. Drug Deliv..

[57] Boyuklieva R., Katsarov P., Zagorchev P., Abarova S., Hristozova A., Pilicheva B. (2024). Idebenone-Loaded Nanocomposite Microspheres for Nasal Administration—A Perspective in the Treatment of Alzheimer’s Disease. Discov. Med..

[58] Muthukumaran K., Leahy S., Harrison K., Sikorska M., Sandhu J.K., Cohen J., Keshan C., Lopatin D., Miller H., Borowy-Borowski H. (2014). Orally delivered water soluble Coenzyme Q10 (Ubisol-Q10) blocks on-going neurodegeneration in rats exposed to paraquat: Potential for therapeutic application in Parkinson’s disease. BMC Neurosci..

[59] Onoue S., Terasawa N., Nakamura T., Yuminoki K., Hashimoto N., Yamada S. (2014). Biopharmaceutical characterization of nanocrystalline solid dispersion of coenzyme Q10 prepared with cold wet-milling system. Eur. J. Pharm. Sci..

[60] Yang R., Li Y., Li J., Liu C., Du P., Zhang T. (2018). Application of scCO_2_ technology for preparing CoQ_10_ solid dispersion and SFC-MS/MS for analyzing in vivo bioavailability. Drug Dev. Ind. Pharm..

[61] Fišar Z., Jirák R., Zvěřová M., Setnička V., Habartová L., Hroudová J., Vaníčková Z., Raboch J. (2019). Plasma amyloid beta levels and platelet mitochondrial respiration in patients with Alzheimer’s disease. Clin. Biochem..

[62] Naderi J., Lopez C., Pandey S. (2006). Chronically increased oxidative stress in fibroblasts from Alzheimer’s disease patients causes early senescence and renders resistance to apoptosis by oxidative stress. Mech. Ageing Dev..

[63] Naderi J., Somayajulu-Nitu M., Mukerji A., Sharda P., Sikorska M., Borowy-Borowski H., Antonsson B., Pandey S. (2006). Water-soluble formulation of Coenzyme Q10 inhibits Bax-induced destabilization of mitochondria in mammalian cells. Apoptosis.

[64] Sehgal N., Gupta A., Valli R.K., Joshi S.D., Mills J.T., Hamel E., Khanna P., Jain S.C., Thakur S.S., Ravindranath V. (2012). *Withania somnifera* reverses Alzheimer’s disease pathology by enhancing low-density lipoprotein receptor-related protein in liver. Proc. Natl. Acad. Sci. USA.

[65] Dar N.J., Ahmad M. (2020). Neurodegenerative diseases and *Withania somnifera* (L.): An update. J. Ethnopharmacol..

[66] Limanaqi F., Biagioni F., Busceti C.L., Ryskalin L., Polzella M., Frati A., Fornai F. (2019). Phytochemicals Bridging Autophagy Induction and Alpha-Synuclein Degradation in Parkinsonism. Int. J. Mol. Sci..

[67] Kumar P., Banik S.P., Goel A., Chakraborty S., Bagchi M., Bagchi D. (2024). Revisiting the Multifaceted Therapeutic Potential of Withaferin A (WA), a Novel Steroidal Lactone, W-ferinAmax Ashwagandha, from *Withania Somnifera* (L) Dunal. J. Am. Nutr. Assoc..

[68] White P.T., Subramanian C., Motiwala H.F., Cohen M.S. (2016). Natural Withanolides in the Treatment of Chronic Diseases. Adv. Exp. Med. Biol..

[69] Zhao M., Wang B., Zhang C., Su Z., Guo B., Zhao Y., Zheng R. (2021). The DJ1-Nrf2-STING axis mediates the neuroprotective effects of Withaferin A in Parkinson’s disease. Cell Death Differ..

[70] Mantle D., Heaton R.A., Hargreaves I.P. (2021). Coenzyme Q10 and Immune Function: An Overview. Antioxidants.

[71] Cristóvão A.C., Campos F.L., Je G., Esteves M., Guhathakurta S., Yang L., Beal M.F., Fonseca B.M., Salgado A.J., Queiroz J. (2020). Characterization of a Parkinson’s disease rat model using an upgraded paraquat exposure paradigm. Eur. J. Neurosci..

[72] Kuboyama T., Tohda C., Komatsu K. (2005). Neuritic regeneration and synaptic reconstruction induced by withanolide A. Br. J. Pharmacol..

[73] Zahiruddin S., Basist P., Parveen A., Parveen R., Khan W., Gaurav Ahmad S. (2020). Ashwagandha in brain disorders: A review of recent developments. J. Ethnopharmacol..

[74] Fišar Z., Hroudová J. (2024). CoQ_10_ and Mitochondrial Dysfunction in Alzheimer’s Disease. Antioxidants.

[75] Farooqui A.A., Farooqui T., Madan A., Ong J.H.-J., Ong W.-Y. (2018). Ayurvedic Medicine for the Treatment of Dementia: Mechanistic Aspects. Evid.-Based Complement. Altern. Med..

[76] Oakley H., Cole S.L., Logan S., Maus E., Shao P., Craft J., Guillozet-Bongaarts A., Ohno M., Disterhoft J., Van Eldik L. (2006). Intraneuronal beta-amyloid aggregates, neurodegeneration, and neuron loss in transgenic mice with five familial Alzheimer’s disease mutations: Potential factors in amyloid plaque formation. J. Neurosci..

[77] Zhu M., Gao W., Wang X., Shi C., Lin Z. (2012). Progression of Corpus Callosum Atrophy in Early Stage of Alzheimer’s Disease. Acad. Radiol..

[78] Au C.K.F., Abrigo J., Liu C., Liu W., Lee J., Au L.W.C., Chan Q., Chen S., Leung E.Y.L., Ho C.L. (2021). Quantitative Susceptibility Mapping of the Hippocampal Fimbria in Alzheimer’s Disease. J. Magn. Reson. Imaging.

[79] Nowrangi M.A., Rosenberg P.B. (2015). The Fornix in Mild Cognitive Impairment and Alzheimer’s Disease. Front. Aging Neurosci..

[80] Bugiani M., Plug B.C., Man J.H.K., Breur M., van der Knaap M.S. (2022). Heterogeneity of white matter astrocytes in the human brain. Acta Neuropathol..

[81] Demer J.L., Dushyanth A. (2011). T2-weighted fast spin-echo magnetic resonance imaging of extraocular muscles. J. Am. Assoc. Pediatr. Ophthalmol. Strabismus.

[82] Lein E.S., Hawrylycz M.J., Ao N., Ayres M., Bensinger A., Bernard A., Boe A.F., Boguski M.S., Brockway K.S., Byrnes E.J. (2007). Genome-wide atlas of gene expression in the adult mouse brain. Nature.

[83] Wang Q., Ding S.-L., Li Y., Royall J., Feng D., Lesnar P., Graddis N., Naeemi M., Facer B., Ho A. (2020). The Allen Mouse Brain Common Coordinate Framework: A 3D Reference Atlas. Cell.

[84] Weller R.O., Hawkes C.A., Kalaria R.N., Werring D.J., Carare R.O. (2015). White matter changes in dementia: Role of impaired drainage of interstitial fluid. Brain Pathol..

[85] Mecca A.P., Chen M., O’Dell R.S., Naganawa M., Toyonaga T., Godek T.A., Harris J.E., Bartlett H.H., Zhao W., Nabulsi N.B. (2020). In vivo measurement of widespread synaptic loss in Alzheimer’s disease with SV2A PET. Alzheimers Dement..

[86] Colom-Cadena M., Spires-Jones T., Zetterberg H., Blennow K., Caggiano A., DeKosky S.T., Fillit H., Harrison J.E., Schneider L.S., Scheltens P. (2020). The clinical promise of biomarkers of synapse damage or loss in Alzheimer’s disease. Alzheimers Res. Ther..

[87] Scheff S.W., Price D.A. (2006). Alzheimer’s disease-related alterations in synaptic density: Neocortex and hippocampus. J. Alzheimers Dis..

[88] DeKosky S.T., Scheff S.W., Styren S.D. (1996). Structural Correlates of Cognition in Dementia: Quantification and Assessment of Synapse Change. Neurodegeneration.

[89] Terry R.D., Masliah E., Salmon D.P., Butters N., DeTeresa R., Hill R., Hansen L.A., Katzman R. (1991). Physical basis of cognitive alterations in alzheimer’s disease: Synapse loss is the major correlate of cognitive impairment. Ann. Neurol..

[90] Mijalkov M., Volpe G., Fernaud-Espinosa I., DeFelipe J., Pereira J.B., Merino-Serrais P. (2021). Dendritic spines are lost in clusters in Alzheimer’s disease. Sci. Rep..

[91] Çetereisi D., Kramvis I., Gebuis T., van der Loo R.J., Gouwenberg Y., Mansvelder H.D., Li K.W., Smit A.B., Spijker S. (2019). Gpr158 Deficiency Impacts Hippocampal CA1 Neuronal Excitability, Dendritic Architecture, and Affects Spatial Learning. Front. Cell. Neurosci..

[92] Moser M.B., Trommald M., Andersen P. (1994). An increase in dendritic spine density on hippocampal CA1 pyramidal cells following spatial learning in adult rats suggests the formation of new synapses. Proc. Natl. Acad. Sci. USA.

[93] Du F. (2019). Golgi-Cox Staining of Neuronal Dendrites and Dendritic Spines with FD Rapid GolgiStain^TM^ Kit. Curr. Protoc. Neurosci..

[94] Risher W.C., Ustunkaya T., Singh Alvarado J., Eroglu C. (2014). Rapid Golgi Analysis Method for Efficient and Unbiased Classification of Dendritic Spines. PLoS ONE.

[95] Lim E.Y., Ho S.H., Hong Y.J., Jeong J.H., Park H.K., Park K.H., Kim S.Y., Wang M.J., Choi S.H., Shim Y.S. (2023). Clinical Significance of Physical Frailty in Subjects with Subjective Cognitive Decline: A Prospective Study with Amyloid PET Data. J. Clin. Neurol..

[96] Whitehead J.C., Hildebrand B.A., Sun M., Rockwood M.R., Rose R.A., Rockwood K., Howlett S.E. (2014). A Clinical Frailty Index in Aging Mice: Comparisons with Frailty Index Data in Humans. J. Gerontol. Ser. A.

[97] Pulok M.H., Theou O., van der Valk A.M., Rockwood K. (2020). The role of illness acuity on the association between frailty and mortality in emergency department patients referred to internal medicine. Age Ageing.

[98] Howlett S.E., Rockwood K. (2014). Ageing: Develop models of frailty. Nature.

[99] Rockwood K. (2005). A global clinical measure of fitness and frailty in elderly people. Can. Med. Assoc. J..

[100] Fahlström A., Yu Q., Ulfhake B. (2011). Behavioral changes in aging female C57BL/6 mice. Neurobiol. Aging.

[101] Ullman-Culleré M.H., Foltz C.J. (1999). Body condition scoring: A rapid and accurate method for assessing health status in mice. Lab. Anim. Sci..

[102] Brayton C., Justice M., Montgomery C.A. (2001). Evaluating Mutant Mice: Anatomic Pathology. Vet. Pathol..

[103] van Meer P., Raber J. (2005). Mouse behavioural analysis in systems biology. Biochem. J..

[104] Strozik E., Festing M.F.W. (1981). Whisker trimming in mice. Lab. Anim..

[105] Blackwell B.-N., Bucci T.J., Hart R.W., Turturro A. (1995). Longevity, Body Weight, and Neoplasia in Ad Libitum-Fed and Diet-Restricted C57BL6 Mice Fed NIH-31 Open Formula Diet. Toxicol. Pathol..

[106] Yerger J., Cougnoux A.C., Abbott C.B., Luke R., Clark T.S., Cawley N.X., Porter F.D., Davidson C.D. (2022). Phenotype assessment for neurodegenerative murine models with ataxia and application to Niemann–Pick disease, type C1. Biol. Open.

[107] Liu Y.B., Tewari A., Salameh J., Arystarkhova E., Hampton T.G., Brashear A., Ozelius L.J., Khodakhah K., Sweadner K.J. (2015). A dystonia-like movement disorder with brain and spinal neuronal defects is caused by mutation of the mouse laminin β1 subunit, Lamb1. eLife.

[108] Shiga A., Nakagawa T., Nakayama M., Endo T., Iguchi F., Kim T.-S., Naito Y., Ito J. (2005). Aging effects on vestibulo-ocular responses in C57BL/6 mice: Comparison with alteration in auditory function. Audiol. Neurootol..

[109] Wolf N.S., Li Y., Pendergrass W., Schmeider C., Turturro A. (2000). Normal Mouse and Rat Strains as Models for Age-related Cataract and the Effect of Caloric Restriction on its Development. Exp. Eye Res..

[110] Zhang J., Yan H., Löfgren S., Tian X., Lou M.F. (2012). Ultraviolet Radiation–Induced Cataract in Mice: The Effect of Age and the Potential Biochemical Mechanism. Investig. Ophthalmol. Vis. Sci..

[111] Van Winkle T.J., Balk M.W. (1986). Spontaneous corneal opacities in laboratory mice. Lab. Anim. Sci..

[112] Chen V.M., Shelke R., Nyström A., Laver N., Sampson J.F., Zhiyi C., Bhat N., Panjwani N. (2018). Collagen VII deficient mice show morphologic and histologic corneal changes that phenotypically mimic human dystrophic epidermolysis bullosa of the eye. Exp. Eye Res..

[113] Blizzard L.E., Menke C., Patel S.D., Waclaw R.R., Lachke S.A., Stottmann R.W. (2021). A Novel Mutation in Cse1l Disrupts Brain and Eye Development with Specific Effects on Pax6 Expression. J. Dev. Biol..

[114] Lehmann K., Schmidt K.-F., Löwel S. (2012). Vision and visual plasticity in ageing mice. Restor. Neurol. Neurosci..

[115] Gelatt K.N. (1997). Visual disturbance: Where do I look?. J. Small Anim. Pract..

[116] Abramowitch S.D., Feola A., Jallah Z., Moalli P.A. (2009). Tissue mechanics, animal models, and pelvic organ prolapse: A review. Eur. J. Obstet. Gynecol. Reprod. Biol..

[117] Brayton C. (2007). Spontaneous Diseases in Commonly Used Mouse Strains. The Mouse in Biomedical Research.

[118] Percy D.H., Barthold S.W. (2008). Pathology of Laboratory Rodents and Rabbits.

[119] Torres-González E., Bueno M., Tanaka A., Krug L.T., Cheng D.-S., Polosukhin V.V., Sorescu D., Lawson W.E., Blackwell T.S., Rojas M. (2012). Role of endoplasmic reticulum stress in age-related susceptibility to lung fibrosis. Am. J. Respir. Cell Mol. Biol..

[120] Defensor E.B., Corley M.J., Blanchard R.J., Blanchard D.C. (2012). Facial expressions of mice in aggressive and fearful contexts. Physiol. Behav..

[121] Langford D.J., Bailey A.L., Chanda M.L., Clarke S.E., Drummond T.E., Echols S., Glick S., Ingrao J., Klassen-Ross T., LaCroix-Fralish M.L. (2010). Coding of facial expressions of pain in the laboratory mouse. Nat. Methods.

[122] Trammell R.A., Cox L., Toth L.A. (2012). Markers for heightened monitoring, imminent death, and euthanasia in aged inbred mice. Comp. Med..

[123] Russell L.N., Hyatt W.S., Gannon B.M., Simecka C.M., Randolph M.M., Fantegrossi W.E. (2021). Effects of Laboratory Housing Conditions on Core Temperature and Locomotor Activity in Mice. J. Am. Assoc. Lab. Anim. Sci..

[124] Wong A.A., Brown R.E. (2007). Age-related changes in visual acuity, learning and memory in C57BL/6J and DBA/2J mice. Neurobiol. Aging.

[125] Benice T.S., Raber J. (2008). Object recognition analysis in mice using nose-point digital video tracking. J. Neurosci. Methods.

[126] Spear N.E., Miller R.R. (2014). Information Processing in Animals.

[127] Oswald I. (1965). Perception and the Conditioned Reflex. By N Sokolov. Translated by Stefan W. Waydenfeld Oxford: Pergamon Press. 1963. Pp. 308. Price 80s. Br. J. Psychiatry.

[128] Baddeley A. (1992). Working Memory: The Interface between Memory and Cognition. J. Cogn. Neurosci..

[129] Olton D.S., Collison C. (1979). Intramaze cues and “odor trails” fail to direct choice behavior on an elevated maze. Anim. Learn. Behav..

[130] Ennaceur A., Delacour J. (1988). A new one-trial test for neurobiological studies of memory in rats. 1: Behavioral data. Behav. Brain Res..

[131] Yang X., Yang Y., Li G., Wang J., Yang E.S. (2008). Coenzyme Q10 Attenuates β-Amyloid Pathology in the Aged Transgenic Mice with Alzheimer Presenilin 1 Mutation. J. Mol. Neurosci..

[132] Singh A., Kumar A. (2015). Microglial Inhibitory Mechanism of Coenzyme Q10 Against Aβ (1-42) Induced Cognitive Dysfunctions: Possible Behavioral, Biochemical, Cellular, and Histopathological Alterations. Front. Pharmacol..

[133] Li G., Jack C.R., Yang X., Yang E.S. (2008). Diet supplement CoQ_10_ delays brain atrophy in aged transgenic mice with mutations in the amyloid precursor protein: An in vivo volume MRI study. BioFactors.

[134] Bagheri S., Haddadi R., Saki S., Kourosh-Arami M., Rashno M., Mojaver A., Komaki A. (2023). Neuroprotective effects of coenzyme Q10 on neurological diseases: A review article. Front. Neurosci..

[135] Sandhu J.K., Pandey S., Ribecco-Lutkiewicz M., Monette R., Borowy-Borowski H., Walker P.R., Sikorska M. (2003). Molecular mechanisms of glutamate neurotoxicity in mixed cultures of NT2-derived neurons and astrocytes: Protective effects of coenzyme Q_10_. J. Neurosci. Res..

[136] Gladen-Kolarsky N., Monestime O., Bollen M., Choi J., Yang L., Magaña A.A., Maier C.S., Soumyanath A., Gray N.E. (2024). *Withania somnifera* (Ashwagandha) Improves Spatial Memory, Anxiety and Depressive-like Behavior in the 5xFAD Mouse Model of Alzheimer’s Disease. Antioxidants.

[137] Kasai H., Fukuda M., Watanabe S., Hayashi-Takagi A., Noguchi J. (2010). Structural dynamics of dendritic spines in memory and cognition. Trends Neurosci..

[138] Kamarulzaman N.T., Makpol S. (2025). The link between Mitochondria and Sarcopenia. J. Physiol. Biochem..

[139] Salve J., Pate S., Debnath K., Langade D. (2019). Adaptogenic and Anxiolytic Effects of Ashwagandha Root Extract in Healthy Adults: A Double-blind, Randomized, Placebo-controlled Clinical Study. Cureus.

[140] Prokopczyk K., Peszt M.J., Jakubowska P., Poręba K., Poręba M., Wasilczuk A., Szaryński M., Lewandowska-Mackiewicz A., Rusiłowicz R., Matuszewska J. (2025). Effects of Ashwagandha Supplementation on Strength, Endurance, and Post-Exercise Recovery: A Review. Qual. Sport.

[141] Gupta M., Kaur G. (2018). *Withania somnifera* as a Potential Anxiolytic and Anti-inflammatory Candidate Against Systemic Lipopolysaccharide-Induced Neuroinflammation. Neuromolecular Med..

[142] Sepúlveda M., Palomo I., Lagos F., Pardo X., López-Alarcón C., Ávila F., Fuentes E. (2026). Biomarkers of oxidative damage as a tool to investigate frailty syndrome in older women. npj Aging.

[143] Buskila Y., Crowe S.E., Ellis-Davies G.C.R. (2013). Synaptic deficits in layer 5 neurons precede overt structural decay in 5xFAD mice. Neuroscience.

[144] Choudhary D., Bhattacharyya S., Bose S. (2017). Efficacy and Safety of Ashwagandha (*Withania somnifera* (L.) *Dunal*) Root Extract in Improving Memory and Cognitive Functions. J. Diet. Suppl..

[145] Barbaresi P., Fabri M., Lorenzi T., Sagrati A., Morroni M. (2024). Intrinsic organization of the corpus callosum. Front. Physiol..

[146] Akiyama H. (2000). Inflammation and Alzheimer’s disease. Neurobiol. Aging.

[147] Hwang H.J., Kim Y.K. (2023). The role of LC3B in autophagy as an RNA-binding protein. Autophagy.

[148] Kang R., Zeh H.J., Lotze M.T., Tang D. (2011). The Beclin 1 network regulates autophagy and apoptosis. Cell Death Differ..

[149] Nixon R.A. (2007). Autophagy, amyloidogenesis and Alzheimer disease. J. Cell Sci..

[150] Yu W., Li Y., Li Y., Hu J., Wu J., Chen X., Huang Y., Shi X. (2025). Connexin43 Contributes to Alzheimer’s Disease by Promoting the Mitochondria-Associated Membrane-Related Autophagy Inhibition. Mol. Neurobiol..

[151] Thompson R.F., Spencer W.A. (1966). Habituation: A model phenomenon for the study of neuronal substrates of behavior. Psychol. Rev..

[152] King D., Holt K., Toombs J., He X., Dando O., Okely J.A., Tzioras M., Rose J., Gunn C., Correia A. (2023). Synaptic resilience is associated with maintained cognition during ageing. Alzheimers Dement..

[153] Kanaan R.A., Allin M., Picchioni M., Barker G.J., Daly E., Shergill S.S., Woolley J., McGuire P.K. (2012). Gender Differences in White Matter Microstructure. PLoS ONE.

[154] Cerghet M., Skoff R.P., Bessert D., Zhang Z., Mullins C., Ghandour M.S. (2006). Proliferation and Death of Oligodendrocytes and Myelin Proteins Are Differentially Regulated in Male and Female Rodents. J. Neurosci..

[155] Raikes A.C., Dyke J.P., Nerattini M., Boneu C., Ajila T., Fauci F., Battista M., Pahlajani S., Williams S., Brinton R.D. (2025). White matter microstructural and macrostructural profiles during midlife reveal sex differences between men and women at different menopausal stages. Sci. Rep..

[156] Ding Z.-B., Song L.-J., Wang Q., Kumar G., Yan Y.-Q., Ma C.-G. (2021). Astrocytes: A double-edged sword in neurodegenerative diseases. Neural Regen. Res..

[157] Heo S., Zhang S., Mun D.-G., Pandey A., Bygrave A.M., Huganir R.L. (2026). Sex- and experience-dependent regulation of synaptic protein turnover. Proc. Natl. Acad. Sci. USA.

[158] Hara Y., Waters E.M., McEwen B.S., Morrison J.H. (2015). Estrogen Effects on Cognitive and Synaptic Health Over the Lifecourse. Physiol. Rev..

[159] Handley E.E., Reale L.A., Chuckowree J.A., Dyer M.S., Barnett G.L., Clark C.M., Bennett W., Dickson T.C., Blizzard C.A. (2022). Estrogen Enhances Dendrite Spine Function and Recovers Deficits in Neuroplasticity in the prpTDP-43A315T Mouse Model of Amyotrophic Lateral Sclerosis. Mol. Neurobiol..

[160] Lin C.-W., Chen B., Huang K.-L., Dai Y.-S., Teng H.-L. (2016). Inhibition of Autophagy by Estradiol Promotes Locomotor Recovery after Spinal Cord Injury in Rats. Neurosci. Bull..

[161] Du L., Hickey R.W., Bayir H., Watkins S.C., Tyurin V.A., Guo F., Kochanek P.M., Jenkins L.W., Ren J., Gibson G. (2009). Starving Neurons Show Sex Difference in Autophagy. J. Biol. Chem..

[162] Nair A., Jacob S. (2016). A simple practice guide for dose conversion between animals and human. J. Basic Clin. Pharm..

[163] Benice T.S., Rizk A., Kohama S., Pfankuch T., Raber J. (2006). Sex-differences in age-related cognitive decline in C57BL/6J mice associated with increased brain microtubule-associated protein 2 and synaptophysin immunoreactivity. Neuroscience.

[164] Kumar P., Banik S.P., Goel A., Chakraborty S., Bagchi M., Bagchi D. (2023). A critical assessment of the whole plant-based phytotherapeutics from *Withania somnifera* (L.) *Dunal* with respect to safety and efficacy vis-a-vis leaf or root extract-based formulation. Toxicol. Mech. Methods.

[165] Kumar S., Singh R., Gajbhiye N., Dhanani T. (2018). Extraction Optimization for Phenolic- and Withanolide-Rich Fractions from *Withania somnifera* Roots: Identification and Quantification of Withaferin A, 12-Deoxywithastromonolide, and Withanolide A in Plant Materials and Marketed Formulations Using a Reversed-Phase HPLC–Photodiode Array Detection Method. J. AOAC Int..

[166] Zellner L., Wierer M., Himmelsbach M., Schatzmann B., Thalhamer B., Klampfl C.W. (2025). Determination of Withanolides and Withanosides in Ashwagandha Based Products Using HPLC-Drift-Tube-Ion-Mobility Quadrupole Time-of-Flight Mass Spectrometry. Electrophoresis.

