# Supplementary material for: Multidimensional Neuroprotective Effects of Combined Ubisol-Q10 and Ashwagandha (Withania somnifera) in Male and Female 5XFAD Mice
Source: Nutrients. 2026 Oct 5;18(19):3270. doi: 10.3390/nu18193270 (PMC13667026; doi:10.3390/nu18193270)
Supplement: Supplementary file 1 [file nutrients-18-03270-s001.zip › nutrients-4561711-supplementary.pdf]

Supplementary Materials

Table S1. (**A**) Two-way repeated-measures analysis of variance (ANOVA) summary of magnetic resonance imaging (MRI) brain region segmentation results. (**B**) Bonferroni-adjusted multiple comparisons of magnetic resonance imaging (MRI) brain region segmentation results.

(A)

Sex	Brain Region	Effect	*F*(df)	*p*	Partial η²	Cohen's *f*
Female	Fimbria	Time	F(1.53, 29.08) = 0.53	0.548	0.027	0.167
		Group	F(2,19) = 11.48	**0.0005**	0. 644	1.346
		Time × Group	F(3.06,29.08) = 1.68	0.192	0.150	0.421
	Anterior Commissure of Olfactory	Time	F(1.79,34.04) = 0.50	0.592	0.026	0.162
		Group	F(2,19) = 10.40	**0.0009**	0.287	0.635
		Time × Group	F(3.58,34.04) = 0.33	0.838	0.033	0.186
	Corpus Callosum	Time	F(1.61,30.58) = 0.97	0.375	0.048	0.226
		Group	F(2,19) = 7.32	**0.004**	0.696	1.514
		Time × Group	F(3.22,30.58) = 3.41	**0.027**	0.264	0.599
	Hippocampus	Time	F(1.77,33.69) = 10.85	**0.0004**	0.363	0.756
		Group	F(2,19) = 7.32	**0.004**	0.455	0.914
		Time × Group	F(3.546,33.69) = 1.25	0.310	0.116	0.362
Male	Fimbria	Time	F(1.33,26.53) = 40.18	**<0.0001**	0.668	1.417
		Group	F(2,20) = 0.81	0.457	0.031	0.181
		Time × Group	F(2.65,26.53) = 0.42	0.717	0.040	0.205
	Anterior Commissure of Olfactory	Time	F(1.82,36.48) = 0.59	0.543	0.029	0.172
		Group	F(2,20) = 0.78	0.470	0.049	0.227
		Time × Group	F(3.65,36.48) = 1.23	0.317	0.109	0.350
	Corpus Callosum	Time	F(1.52,30.32) = 53.09	**<0.0001**	0.726	1.629
		Group	F(2,20) = 2.58	0.101	0.235	0.553
		Time × Group	F(3.03,30.32) = 2.40	0.087	0.194	0.490
	Hippocampus	Time	F(1.50,29.96) = 82.86	**<0.0001**	0.806	2.035
		Group	F(2,20) = 17.74	**<0.0001**	0.726	1.627
		Time × Group	F(3.00,29.96) = 0.91	0.450	0.083	0.301

(B)

Sex	Region	Age	Comparison	Mean 1	Mean 2	Mean Difference	SE	95% CI	*t*	df	Bonferroni-Adjusted *p*
Female	Hippocampus	3 mo	5XFAD/Tonic vs. 5XFAD/Control	1947	1818	129.1	72.76	−85.59 to 343.9	1.775	8.758	0.332
			5XFAD/Tonic vs. Wild-Type/Control	1947	2030	−83.54	45.23	−207.9 to 40.82	1.847	12.89	0.264
			5XFAD/Control vs. Wild-Type/Control	1818	2030	−212.7	72.57	−426.8 to 1.489	2.931	8.760	0.052
		6 mo	5XFAD/Tonic vs. 5XFAD/Control	2015	2025	−9.71	67.26	−197.5 to 178.1	0.1444	11.68	1.000 (n.s.)
			5XFAD/Tonic vs. Wild-Type/Control	2015	2159	−143.9	62.09	−314.4 to 26.68	2.317	12.96	0.113
			5XFAD/Control vs. Wild-Type/Control	2025	2159	−134.2	67.89	−321.9 to 53.61	1.976	12.39	0.213

		9 mo	5XFAD/Tonic vs. 5XFAD/Control	2117	1940	177.3	76.85	−36.35 to 390.9	2.307	11.99	0.119
			5XFAD/Tonic vs. Wild-Type/Control	2117	2174	−57.38	82.86	−285.2 to 170.4	0.692	12.90	1.000 (n.s.)
			5XFAD/Control vs. Wild-Type/Control	1940	2174	−234.7	83.87	−465.1 to −4.246	2.798	12.95	**0.045**
Male	Hippocampus	3 mo	5XFAD/Tonic vs. 5XFAD/Control	2134	2012	121.5	49.32	−15.57 to 258.6	2.463	12.01	0.089
			5XFAD/Tonic vs. Wild-Type/Control	2134	2261	−127.4	63.44	−315.6 to 60.84	2.008	8.554	0.232
			5XFAD/Control vs. Wild-Type/Control	2012	2261	−248.9	70.82	−447.9 to −49.93	3.514	11.24	**0.014**
		6 mo	5XFAD/Tonic vs. 5XFAD/Control	2239	2103	135.5	31.68	49.20 to 221.8	4.278	13.75	**0.002**
			5XFAD/Tonic vs. Wild-Type/Control	2239	2482	−242.8	69.39	−454.9 to −30.76	3.499	7.600	**0.026**
			5XFAD/Control vs. Wild-Type/Control	2103	2482	−378.3	68.41	−590.4 to −166.3	5.530	7.225	**0.002**
		9 mo	5XFAD/Tonic vs. 5XFAD/Control	2519	2366	153.8	78.47	−64.51 to 372.0	1.959	11.95	0.222
			5XFAD/Tonic vs. Wild-Type/Control	2519	2719	−200.0	91.59	−467.7 to 67.64	2.184	9.151	0.169
			5XFAD/Control vs. Wild-Type/Control	2366	2719	−353.8	104.6	−644.3 to −63.28	3.383	12.04	**0.016**
Female	Fimbria	3 mo	5XFAD/Tonic vs. 5XFAD/Control	163.7	250.0	−86.29	48.89	−224.2 to 51.64	1.765	10.98	0.316
			5XFAD/Tonic vs. Wild-Type/Control	163.7	122.5	41.21	35.72	−58.96 to 141.4	1.154	11.35	0.817
			5XFAD/Control vs. Wild-Type/Control	250.0	122.5	127.5	44.77	−3.013 to 258.0	2.848	9.264	0.056
		6 mo	5XFAD/Tonic vs. 5XFAD/Control	121.1	340.3	−219.1	68.95	−437.9 to −0.4354	3.178	6.683	**0.049**
			5XFAD/Tonic vs. Wild-Type/Control	121.1	134.5	−13.36	23.33	−77.43 to 50.71	0.5725	12.99	1.000 (n.s.)
			5XFAD/Control vs. Wild-Type/Control	340.3	134.5	205.8	69.18	−12.77 to 424.3	2.975	6.768	0.065
		9 mo	5XFAD/Tonic vs. 5XFAD/Control	151.6	289.0	−137.4	52.38	−302.8 to 27.93	2.624	6.785	0.106
			5XFAD/Tonic vs. Wild-Type/Control	151.6	148.1	3.446	18.01	−46.11 to 53.01	0.1914	12.81	1.000 (n.s.)
			5XFAD/Control vs. Wild-Type/Control	289.0	148.1	140.9	52.24	−24.52 to 306.3	2.696	6.724	0.096
			5XFAD/Control vs. Wild-Type/Control	499.6	497.1	2.482	67.90	−188.6 to 193.5	0.03656	11.14	1.000 (n.s.)
	Anterior Commissure of Olfactory	3 mo	5XFAD/Tonic vs. 5XFAD/Control	93.29	193.6	−100.3	45.50	−235.3 to 34.71	2.204	8.558	0.169
			5XFAD/Tonic vs. Wild-Type/Control	93.29	127.4	−34.09	25.07	−103.7 to 35.56	1.360	12.04	0.597
			5XFAD/Control vs. Wild-Type/Control	193.6	127.4	66.20	44.06	−67.73 to 200.1	1.502	7.758	0.518

	6 mo	5XFAD/Tonic vs. 5XFAD/Control	95.29	162.1	−66.86	32.76	−161.5 to 27.78	2.041	9.671	0.208
		5XFAD/Tonic vs. Wild-Type/Control	95.29	101.9	−6.589	26.70	−80.18 to 67.00	0.2467	12.69	1.000 (n.s.)
		5XFAD/Control vs. Wild-Type/Control	162.1	101.9	60.27	35.21	−38.35 to 158.9	1.712	11.45	0.342
	9 mo	5XFAD/Tonic vs. 5XFAD/Control	86.00	184.0	−98.00	48.32	−251.1 to 55.13	2.028	6.703	0.252
		5XFAD/Tonic vs. Wild-Type/Control	86.00	149.6	−63.63	19.70	−118.2 to −9.010	3.230	12.20	**0.021**
		5XFAD/Control vs. Wild-Type/Control	184.0	149.6	34.38	49.64	−118.4 to 187.2	0.6925	7.402	1.000 (n.s.)
Corpus Callpsum	3 mo	5XFAD/Tonic vs. 5XFAD/Control	1182	1648	−466.9	304.3	−1340 to 406.7	1.534	9.991	0.468
		5XFAD/Tonic vs. Wild-Type/Control	1182	1194	−12.30	182.1	−541.5 to 516.9	0.06757	9.394	1.000 (n.s.)
		5XFAD/Control vs. Wild-Type/Control	1648	1194	454.6	273.3	−388.0 to 1297	1.663	7.358	0.414
	6 mo	5XFAD/Tonic vs. 5XFAD/Control	873.3	2168	−1294	386.6	−2537 to −51.84	3.348	6.412	**0.042**
		5XFAD/Tonic vs. Wild-Type/Control	873.3	1125	−251.5	144.8	−661.2 to 158.3	1.736	10.80	0.333
		5XFAD/Control vs. Wild-Type/Control	2168	1125	1043	400.6	−193.8 to 2279	2.603	7.327	0.102
	9 mo	5XFAD/Tonic vs. 5XFAD/Control	962.6	2035	−1073	372.6	−2233 to 87.51	2.879	7.106	0.070
		5XFAD/Tonic vs. Wild-Type/Control	962.6	1448	−485.3	127.3	−849.9 to −120.7	3.813	10.09	**0.010**
		5XFAD/Control vs. Wild-Type/Control	2035	1448	587.6	362.6	−577.5 to 1753	1.621	6.415	0.459

(**A**) Note. Data were analyzed using two-way repeated-measures ANOVA with time as the repeated-measures factor and experimental group as the between-subjects factor. Geisser–Greenhouse corrections were applied where appropriate. Bolded values indicate $p < 0.05$. (**B**) Note. Bonferroni-adjusted p values < 0.05 were considered statistically significant and are represented as bolded values. Only regions with significant overall group effects or significant time × group interactions are included. "n.s." = not significant.

Table S2. (**A**) One-way analysis of variance (ANOVA) summary of Nissl stain results in corpus callosum. (**B**) Bonferroni-corrected pairwise comparisons of Nissl stain results in corpus callosum.

(**A**)

Sex	5XFAD/Tonic Mean ± SEM	5XFAD/Control Mean ± SEM	Wild-Type/Control Mean ± SEM	*F*(df)	*p*	η²
**Female**	12.468 ± 1.917	14.014 ± 1.196	10.693 ± 2.332	F(2,21) = 0.38	0.690	0.035
**Male**	8.145 ± 0.610	7.822 ± 1.441	9.024 ± 1.222	F(2,20) = 17.62	**<0.001**	0.638

(**B**)

Sex	Comparison	Bonferroni-Adjusted *p*-Value	Cohen's *d*
**Male**	5XFAD/Control vs. 5XFAD/Tonic	1.000 (n.s.)	−0.132
	5XFAD/Tonic vs. Wild-Type/Control	**0.0009**	−2.322
	5XFAD/Control vs. Wild-Type/Control	**0.0004**	−2.511

(**A**) Note. Bolded values represent significance at $p < 0.05$. (**B**) Note. Data were analyzed using one-way ANOVA followed by Bonferroni-corrected pairwise comparisons. Only those which previously demonstrated a significant overall ANOVA are included. Adjusted p values < 0.05 were considered statistically significant. Bolded values represent significance at $p < 0.05$. "n.s." = not significant.

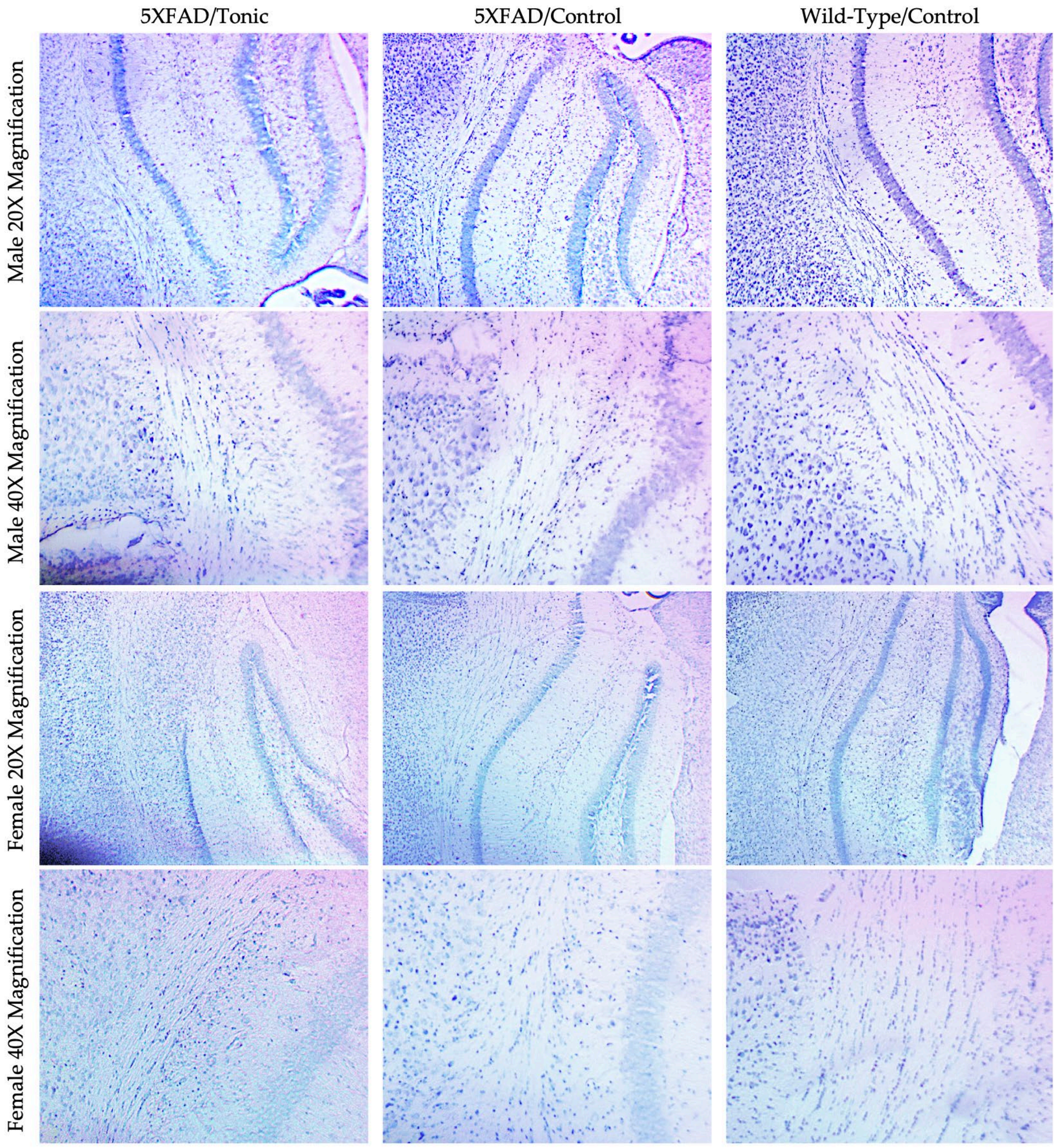

Figure S1. Representative Nissl-stained images (20× and 40× magnification).

Table S3. (**A**) One-way analysis of variance (ANOVA) summary of immunofluorescence results. (**B**) Bonferroni-corrected pairwise comparisons of immunofluorescent results.

(**A**)							
**Marker**	**Sex**	***F*(df)**	***p***	**η^2**	**Wild-Type/Control Mean ± SEM**	**5XFAD/Control Mean ± SEM**	**5XFAD/Tonic Mean ± SEM**
Amyloid-β	Male	F(2,20) = 42.24	**<0.0001**	0.809	0.040 ± 0.007	1.814 ± 0.223	0.212 ± 0.041

	Female	F(2,24) = 11.95	**0.0003**	0.499	0.062 ± 0.034	2.051 ± 0.504	0.686 ± 0.067
APP	Male	F(2,20) = 21.56	**<0.0001**	0.683	0.852 ± 0.195	4.200 ± 0.435	1.753 ± 0.352
	Female	F(2,24) = 16.52	**<0.0001**	0.579	0.290 ± 0.134	1.575 ± 0.179	0.650 ± 0.173
IBA-1	Male	F(2,23) = 45.33	**<0.0001**	0.798	0.373 ± 0.072	3.347 ± 0.322	1.274 ± 0.226
	Female	F(2,19) = 22.50	**<0.0001**	0.703	0.494 ± 0.081	2.094 ± 0.228	0.692 ± 0.194
LC3B	Male	F(2,23) = 14.92	**0.00007**	0.565	3.780 ± 0.523	0.922 ± 0.117	3.482 ± 0.467
	Female	F(2,19) = 1.19	0.325	0.112	0.787 ± 0.207	0.534 ± 0.075	0.748 ± 0.076
IL-1β	Male	F(2,24) = 24.63	**<0.0001**	0.672	0.327 ± 0.120	2.017 ± 0.185	0.785 ± 0.211
	Female	F(2,20) = 4.15	**0.031**	0.293	0.321 ± 0.073	1.275 ± 0.372	0.665 ± 0.199
Beclin-1	Male	F(2,24) = 46.05	**<0.0001**	0.793	3.794 ± 0.228	0.968 ± 0.121	3.707 ± 0.319
	Female	F(2,20) = 1.80	0.191	0.153	0.722 ± 0.159	0.502 ± 0.067	0.807 ± 0.102
Hippocampal α-GFAP	Male	F(2,24) = 11.68	**0.0003**	0.493	0.403 ± 0.045	1.069 ± 0.167	0.434 ± 0.080
	Female	F(2,24) = 0.87	0.434	0.067	1.520 ± 0.383	0.944 ± 0.229	1.270 ± 0.301
Corpus Callosum α-GFAP	Male	F(2,24) = 1.22	0.313	0.092	2.046 ± 0.471	3.473 ± 0.746	2.844 ± 0.692
	Female	F(2,23) = 11.60	**0.0003**	0.502	14.112 ± 1.219	6.033 ± 1.322	13.340 ± 1.306

(**B**)

**Marker**	**Sex**	**Wild-Type/Control vs. 5XFAD/Control (Bonferroni-Adjusted *p*-Value)**	**Cohen's *d***	**Wild-Type/Control vs. 5XFAD/Tonic (Bonferroni-Adjusted *p*-Value)**	**Cohen's *d***	**5XFAD/Control vs. 5XFAD/Tonic (Bonferroni-Adjusted *p*-Value)**	**Cohen's *d***
Amyloid-β	Male	**0.0001**	−3.384	**0.013**	−1.945	**0.0002**	3.241
	Female	**0.013**	−1.856	**<0.0001**	−3.902	0.083	1.266
APP	Male	**<0.0001**	−3.143	0.141	−1.097	**0.002**	2.091
	Female	**0.0001**	−2.711	0.362	−0.775	**0.006**	1.754
Iba-1	Male	**<0.0001**	−4.632	**0.011**	−1.786	**0.0005**	2.606
	Female	**0.0002**	−2.982	1.000 (n.s.)	−0.552	**0.001**	2.287
LC3B	Male	**0.001**	2.446	0.775	0.490	**0.0004**	−2.030
IL-1β	Male	**<0.0001**	−3.616	0.245	−0.890	**0.001**	2.071
	Female	0.136	−1.392	0.416	−0.813	0.546	0.776
Beclin-1	Male	**<0.0001**	5.173	1.000 (n.s.)	0.105	**<0.0001**	−3.783
Hippocampal α-GFAP	Male	**0.012**	−1.816	1.000 (n.s.)	−0.158	**0.017**	1.617
	Female	**0.001**	2.186	1.000 (n.s.)	0.204	**0.004**	−1.905

(**A**) Note. Bolded values represent significance at $p < 0.05$. "APP" = amyloid precursor protein, "Iba-1" = ionized calcium-binding adapter molecule 1, "LC3B" = microtubule-associated protein 1 light chain 3B, "IL-1β" = interleukin-1β, "α-GFAP" = α-glial fibrillary acidic protein. (**B**) Note. Data were analyzed using one-way analysis of variance (ANOVA) followed by Bonferroni-corrected pairwise comparisons. Only those which previously demonstrated a significant overall ANOVA are included. Adjusted *p*-values < 0.05 were considered statistically significant. Bolded values represent significance at $p < 0.05$. "n.s." = not significant, "APP" = amyloid precursor protein, "Iba-1" = ionized calcium-binding adapter molecule 1, " LC3B" = microtubule-associated protein 1 light chain 3B, "IL-1β" = interleukin-1β, "α-GFAP" = α-glial fibrillary acidic protein.

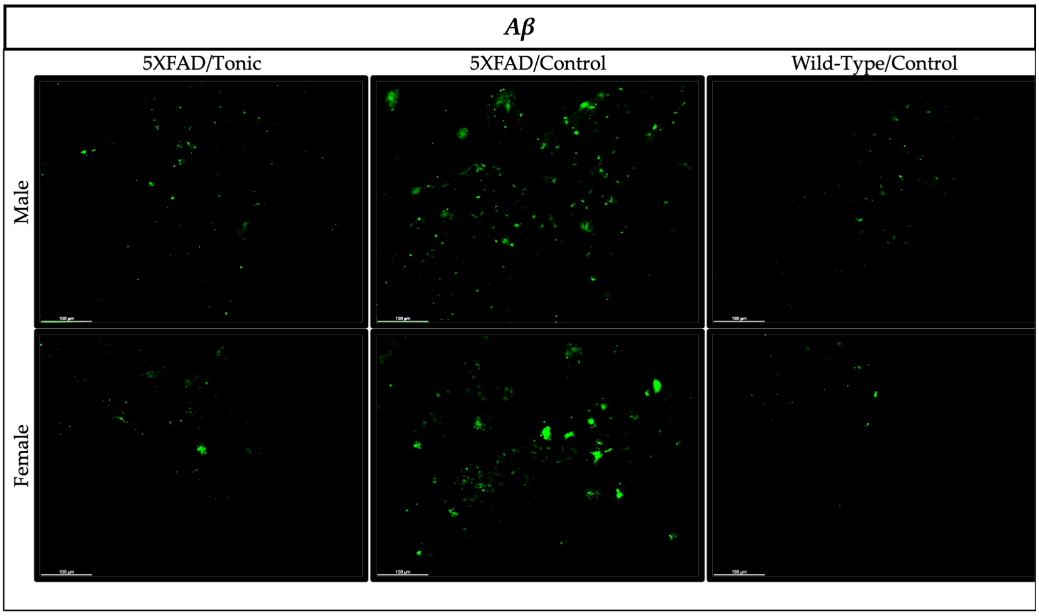
Aβ
5XFAD/Tonic
5XFAD/Control
Wild-Type/Control
Male
Female

(**A**)

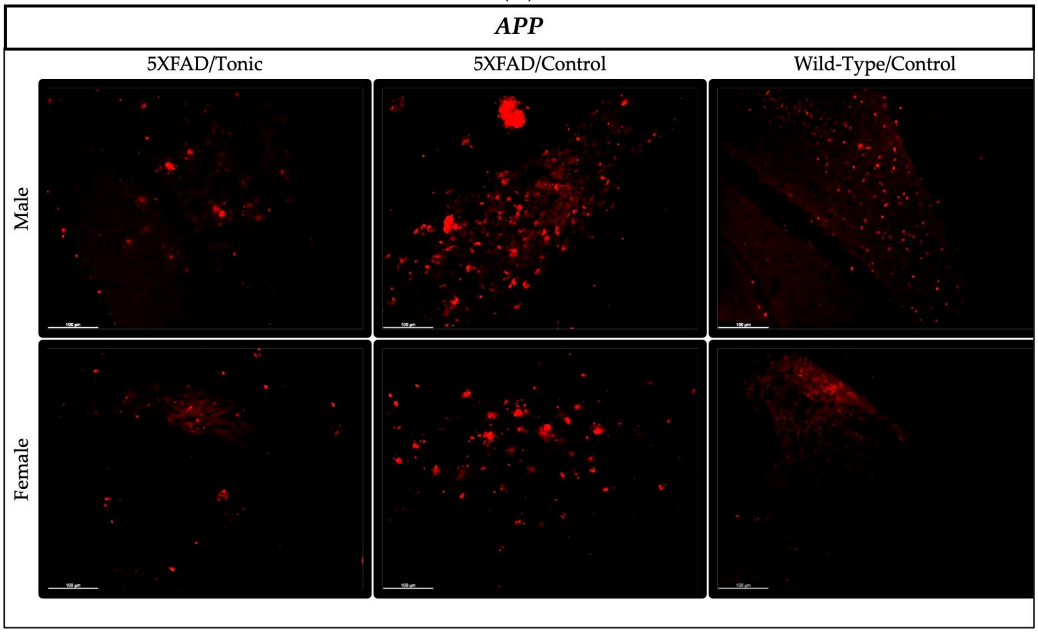
APP
5XFAD/Tonic
5XFAD/Control
Wild-Type/Control
Male
Female

(**B**)

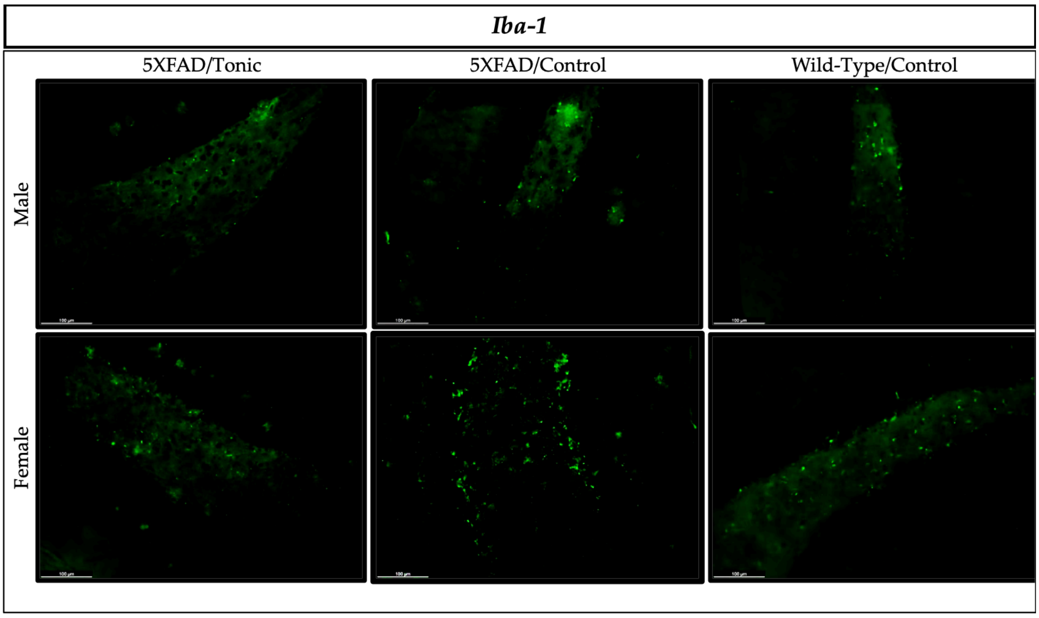
Iba-1
5XFAD/Tonic
5XFAD/Control
Wild-Type/Control
Male
Female

(C)

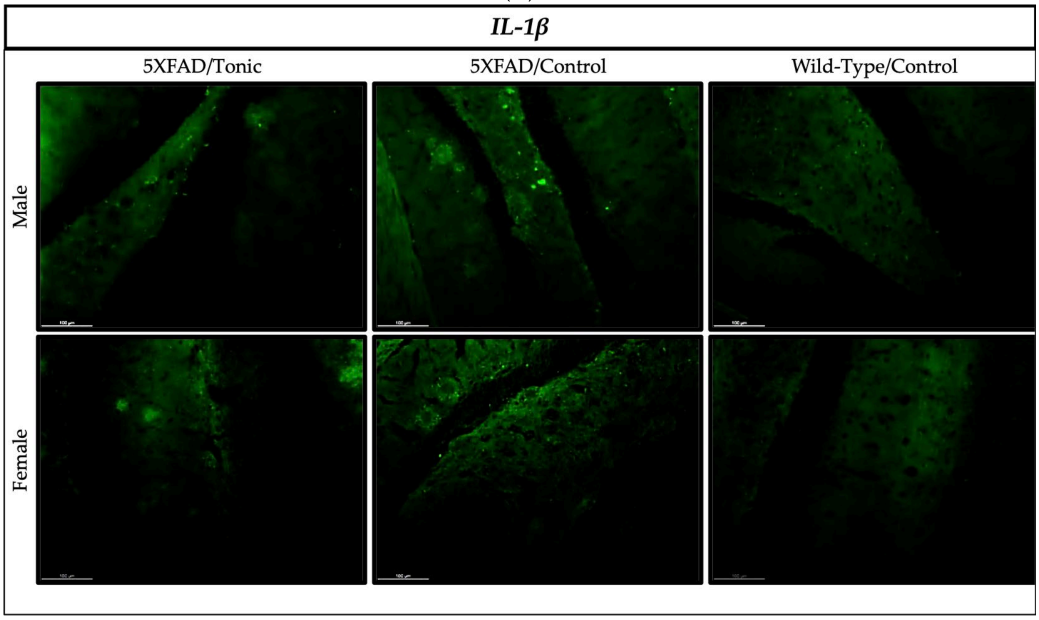
IL-1β
5XFAD/Tonic
5XFAD/Control
Wild-Type/Control
Male
Female

(D)

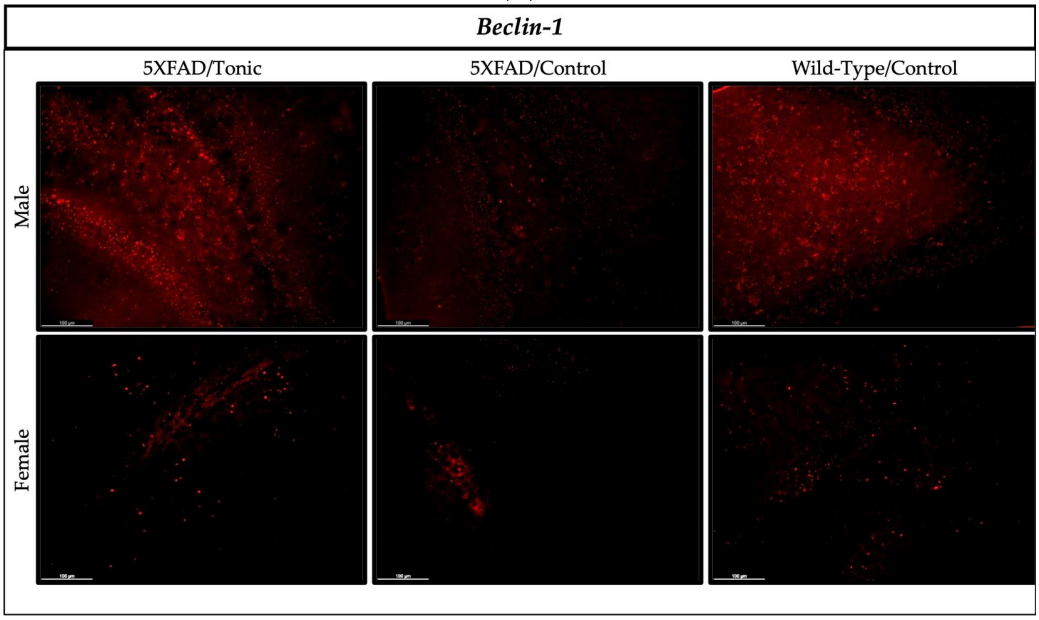
Beclin-1
5XFAD/Tonic
5XFAD/Control
Wild-Type/Control
Male
Female

(**E**)

Figure S2. (**A**) Qualitative representative images of hippocampal amyloid-β (Aβ) immunoreactivity in the dentate gyrus and CA1 (Cornu Ammonis area 1) region of the hippocampus of male and female wild-type/control, 5XFAD/control, and 5XFAD/Tonic-treated mice. Representative images were taken at 20× magnification and scale bar = 100 μm. (**B**) Qualitative representative images of hippocampal amyloid precursor protein (APP) immunoreactivity in the dentate gyrus and CA1 (Cornu Ammonis area 1) region of the hippocampus of male and female wild-type/control, 5XFAD/control, and 5XFAD/Tonic-treated mice. Representative images were taken at 20× magnification and scale bar = 100 μm. (**C**) Qualitative representative images of hippocampal ionized calcium-binding adapter molecule 1 (Iba-1) immunoreactivity in the dentate gyrus and CA1 (Cornu Ammonis area 1) region of the hippocampus of male and female wild-type/control, 5XFAD/control, and 5XFAD/Tonic-treated mice. Representative images were taken at 20× magnification and scale bar = 100 μm. (**D**) Qualitative representative images of hippocampal interleukin-1β (IL-1β) immunoreactivity in the dentate gyrus and CA1 (Cornu Ammonis area 1) region of the hippocampus of male and female wild-type/control, 5XFAD/control, and 5XFAD/Tonic-treated mice. Representative images were taken at 20× magnification and scale bar = 100 μm. (**E**) Qualitative representative images of hippocampal Beclin-1 immunoreactivity in the dentate gyrus and CA1 (Cornu Ammonis area 1) region of the hippocampus of male and female wild-type/control, 5XFAD/control, and 5XFAD/Tonic-treated mice. Representative images were taken at 20× magnification and scale bar = 100 μm.

Table S4. (**A**) One-way analysis of variance (ANOVA) summary of Golgi–Cox staining results. (**B**) Bonferroni-corrected pairwise comparisons of Golgi–Cox staining results.

(**A**)

Evaluation	Sex	5XFAD/Tonic Mean ± SEM	5XFAD/Control Mean ± SEM	Wild-Type/Control Mean ± SEM	*F*(df)	*p*	η^2
Percentage of mature dendrites	Female	0.500 ± 0.056	0.268 ±0.072	0.498 ± 0.010	F(2,6) = 6.27	**0.034**	0.677
	Male	0.624 ± 0.029	0.301 ± 0.030	0.739 ± 0.023	F(2,6) = 68.00	**<0.001**	0.958
Dendritic length	Female	127.218 ± 9.646	83.557 ± 4.142	105.489 ± 4.583	F(2,78) = 10.90	**<0.001**	0.218
	Male	121.038 ± 4.461	97.073 ± 5.098	129.544 ± 6.146	F(2,78) = 10.17	**<0.001**	0.207
Dendritic thickness	Female	1.380 ± 0.092	1.102 ± 0.103	1.567 ± 0.131	F(2,78) = 4.52	**0.014**	0.104
	Male	1.179 ± 0.054	0.957 ± 0.068	1.149 ± 0.048	F(2,78) = 4.40	**0.015**	0.101
Spine density	Female	1.768 ± 0.071	1.240 ± 0.067	1.426 ± 0.064	F(2,78) = 15.66	**<0.001**	0.286
	Male	1.276 ± 0.050	1.097 ± 0.046	1.258 ± 0.071	F(2,78) = 2.98	0.056	0.071

(**B**)

Evaluation	Sex	Comparison	Bonferroni-Adjusted *p*	Cohen's *d*
Mature dendrites (percentage)	Female	5XFAD/Tonic vs. 5XFAD/Control	0.194	2.067
		5XFAD/Tonic vs. Wild-Type/Control	1.000 (n.s.)	0.030
		5XFAD/Control vs. Wild-Type/Control	0.264	−2.569
	Male	5XFAD/Tonic vs. 5XFAD/Control	**0.005**	6.283
		5XFAD/Tonic vs. Wild-Type/Control	0.106	−2.554

		5XFAD/Control vs. Wild-Type/Control	**0.001**	−9.429
Dendritic length (µm)	Female	5XFAD/Tonic vs. 5XFAD/Control	**0.0006**	1.132
		5XFAD/Tonic vs. Wild-Type/Control	0.147	0.554
		5XFAD/Control vs. Wild-Type/Control	**0.003**	−0.966
	Male	5XFAD/Tonic vs. 5XFAD/Control	**0.003**	0.963
		5XFAD/Tonic vs. Wild-Type/Control	0.805	−0.305
		5XFAD/Control vs. Wild-Type/Control	**0.0005**	−1.107
Dendritic thickness (µm)	Female	5XFAD/Tonic vs. 5XFAD/Control	0.151	0.546
		5XFAD/Tonic vs. Wild-Type/Control	0.745	−0.318
		5XFAD/Control vs. Wild-Type/Control	**0.023**	−0.759
	Male	5XFAD/Tonic vs. 5XFAD/Control	**0.041**	0.696
		5XFAD/Tonic vs. Wild-Type/Control	1.000 (n.s.)	0.114
		5XFAD/Control vs. Wild-Type/Control	0.078	−0.626
Spine density (number of dendritic spines/length in µm)	Female	5XFAD/Tonic vs. 5XFAD/Control	**<0.0001**	1.464
		5XFAD/Tonic vs. Wild-Type/Control	**0.035**	0.969
		5XFAD/Control vs. Wild-Type/Control	**0.002**	−0.545
	Male	5XFAD/Tonic vs. 5XFAD/Control	**0.035**	0.711
		5XFAD/Tonic vs. Wild-Type/Control	1.000 (n.s.)	0.054
		5XFAD/Control vs. Wild-Type/Control	0.193	−0.516

(**A**) Note. η^2 values represent the proportion of variance explained by treatment or experimental phase. Bolded values represent significance at $p < 0.05$. (**B**) Note. Bolded values represent significance at $p < 0.05$. "n.s." = not significant.

Table S5. (**A**) Two-way repeated-measures analysis of variance (ANOVA) summary of frailty index results. (**B**) Bonferroni-adjusted multiple comparisons of frailty index results. (**C**) Frailty index criteria and representative photos of how the frailty index was implemented in the current study. The table follows three individual mice through each criteria to show the physiological variation in aging outcomes from the highest scored mouse, a modestly scored mouse, and the lowest scored mouse at 9 months of age. Examples of the highest scored mouse per criteria is also provided if the other three mice do not exemplify this score. Refer to Table 4 for definitions of criteria and explanation of scoring.

(**A**)

**Effect**	***F*(df)**	**Partial η^2**	**Cohen's *f***	***p***
Time × Group Condition	F (3.374, 33.74) = 3.223	0.2438	0.5677	**0.0301**
Time	F (1.687, 33.74) = 138.1	0.8735	2.628	**<0.0001**
Group Condition	F (2, 20) = 19.89	0.4983	0.9967	**<0.0001**

(**B**)

**Timepoint**	**Comparison**	**Mean 1**	**Mean 2**	**Mean Diff.**	**SE of Diff.**	***t***	**DF**	**95.00% CI of Diff.**	**Bonferroni-Adjusted *p***
3 Mo of Age	5XFAD/Tonic vs. 5XFAD/Control	0.969	1.200	−0.231	0.189	1.225	13.50	−0.7467 to 0.2842	0.724
	5XFAD/Tonic vs. Wild-Type/Control	0.969	0.643	0.326	0.161	2.027	12.98	−0.1157 to 0.7675	0.191
	5XFAD/Control vs. Wild-Type/Control	1.200	0.643	0.557	0.181	3.080	12.39	0.05679 to 1.058	**0.028**
6 Mo of Age	5XFAD/Tonic vs. 5XFAD/Control	3.000	3.525	−0.525	0.464	1.132	13.90	−1.786 to 0.7362	0.830
	5XFAD/Tonic vs. Wild-Type/Control	3.000	2.036	0.964	0.364	2.652	11.13	−0.05907 to 1.988	0.067
	5XFAD/Control vs. Wild-Type/Control	3.525	2.036	1.489	0.388	3.836	10.62	0.3877 to 2.591	**0.009**
9 Mo of Age	5XFAD/Tonic vs. 5XFAD/Control	4.750	6.375	−1.625	0.571	2.847	12.63	−3.199 to −0.05082	0.042

5XFAD/Tonic vs. Wild-Type/Control	4.750	3.786	0.964	0.509	1.893	12.32	−0.4457 to 2.374	0.246
5XFAD/Control vs. Wild-Type/Control	6.375	3.786	2.589	0.606	4.276	12.86	0.9240 to 4.255	0.003

(C)

**Criteria**	**Highest Scored Mouse at 9 mo of Age Mouse ID: C2 Condition: 5XFAD/Control**	**Moderately Scored Mouse at 9 mo of ag e Mouse ID: F1 Condition: 5XFAD/Tonic**	**Lowest Scored Mouse at 9 mo of Age Mouse ID: D3 Condition: Wild-Type/Control**	**Examples of Other Mice Given the Highest Score at the 9 mo of Age Timepoint, if Columns to the Left do not Display This Score**
Alopecia	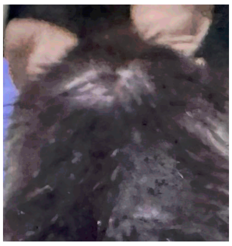 Some localized hair loss Score = 0.25	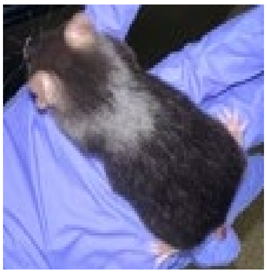 Score = 0	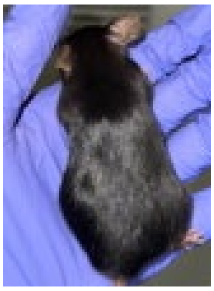 Score = 0	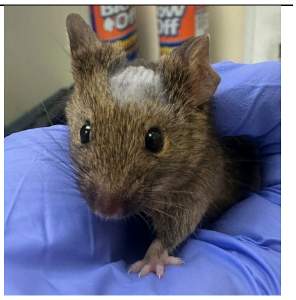 Condition: Wild-Type/Control Score = 1
Loss of fur colour	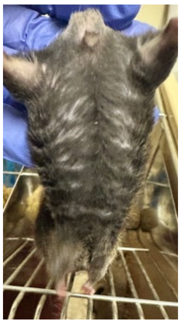 Score = 1	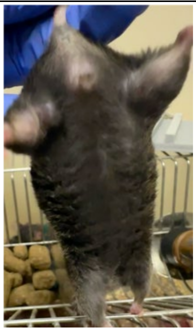 Score = 0.5	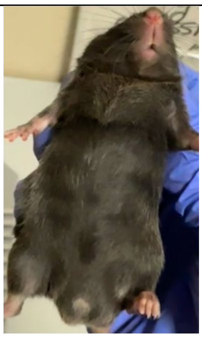 Score = 0.25	N/A
Dermatitis	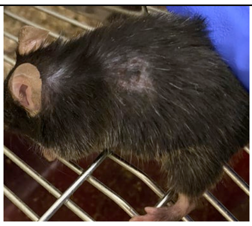 Score = 0.5	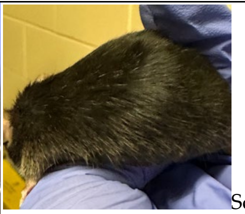 Score = 0	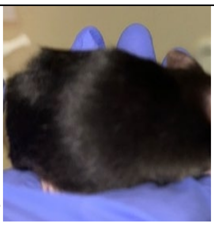 Score = 0	N/A
Loss of whiskers	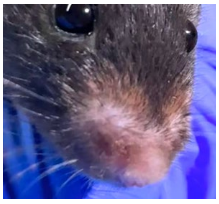 Score = 0.75	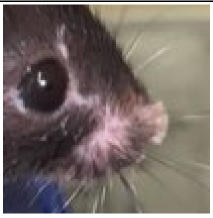 Score = 0.5	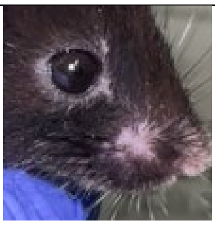 Score = 0.25	N/A
Coat condition	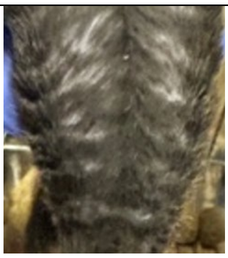 Score = 1	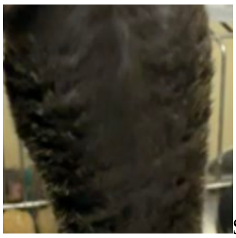 Score = 0.5	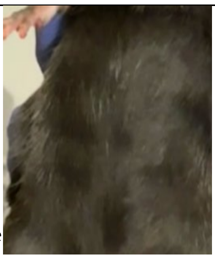 Score = 0	N/A

Kyphosis	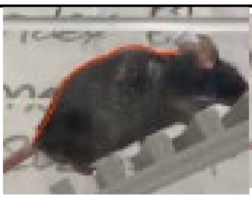<br>Score = 0.75	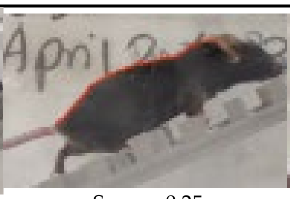<br>Score = 0.25	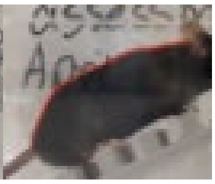<br>Score = 0	N/A
Tail stiffening	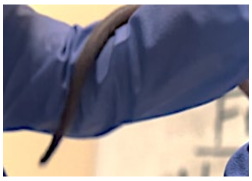<br>Score = 0	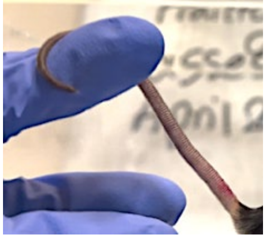<br>Score = 0	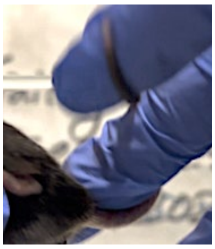<br>Score = 0	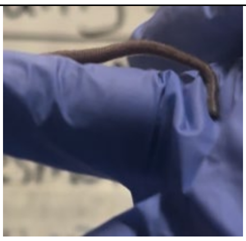<br>Condition: 5XFAD/Tonic<br>Score = 0.75
Gait disorders	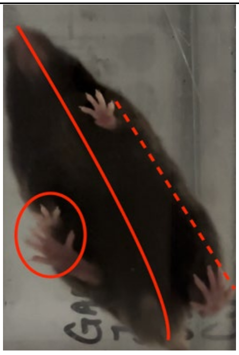<br>Score = 0.25	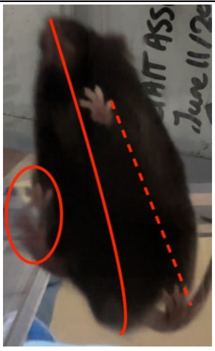<br>Score = 0.5	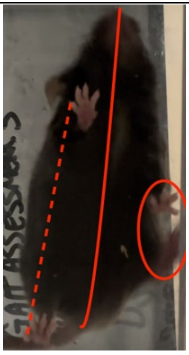<br>Score = 0.25	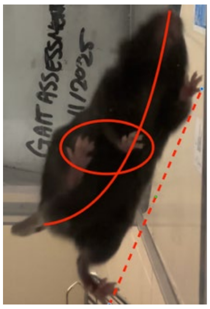<br>Condition: 5XFAD/Control<br>Score = 1
Tremor	No photo<br>Score = 0.5	No photo<br>Score = 0	No photo<br>Score = 0	N/A
Forelimb grip strength	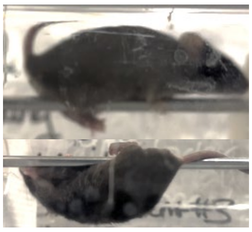<br>Average time on wire = 6 seconds, −5.67 seconds from first assessment (3mo of age)<br>Score = 0.5	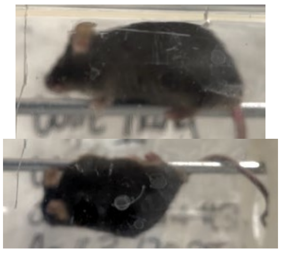<br>Average time on wire = 6.33 seconds, +3.67 seconds from first assessment (3mo of age)<br>Score = 0	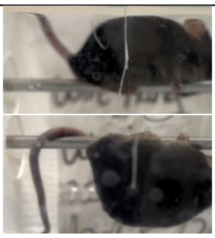<br>Average time on wire = 31 seconds, +18.67 seconds from first assessment (3mo of age)<br>Score = 0	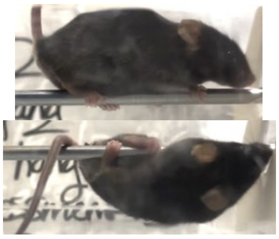<br>Condition: 5XFAD/Tonic<br>Average Time on wire = 20.67, −16.33 from first assessment (3mo of age)<br>Score = 1
Body condition score	See photos for loss of fur colour above or piloerection below<br>Score = 0	See photos for loss of fur colour above or piloerection below<br>Score = 0	See photos for loss of fur colour above or piloerection below<br>Score = 0	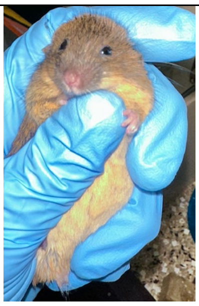<br>Over-conditioned<br>Condition: Wild-Type/Control<br>Score = 0.5

Vestibular disturbance	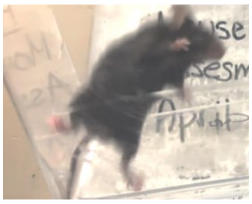<br>Slipped but maintained balance<br>Score = 0.25	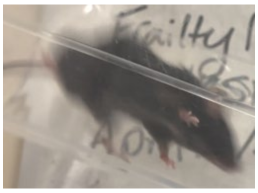<br>Slipped but maintained balance<br>Score = 0.25	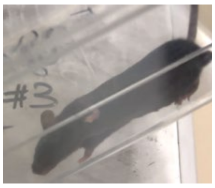<br>All paws remained grounded<br>Score = 0	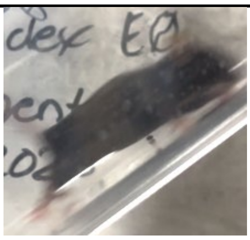<br>Slipped, 1 paw remained grounded<br>Condition: 5XFAD/Control<br>Score = 0.5
Hearing loss	No photo<br>Score = 0.25	No photo<br>Score = 0	No photo<br>Score = 0	N/A
Corneal opacity	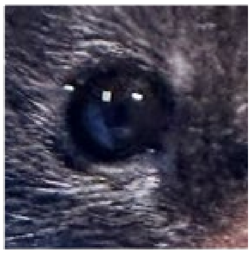<br>Score = 0.25	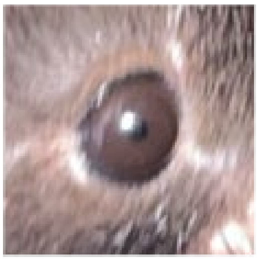<br>Score = 0.5	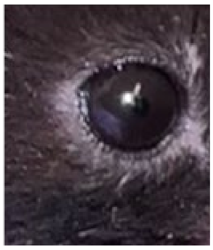<br>Score = 0.25	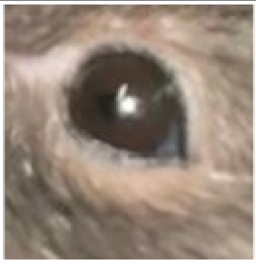<br>Condition: 5XFAD/Control<br>Score = 0.75
Microphthalmia	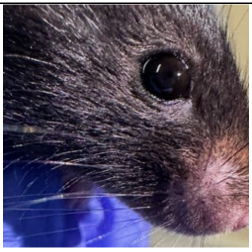<br>Score = 0	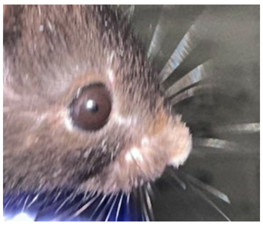<br>Score = 0	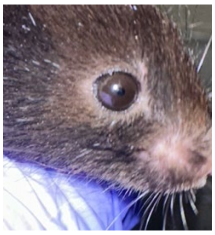<br>Score = 0	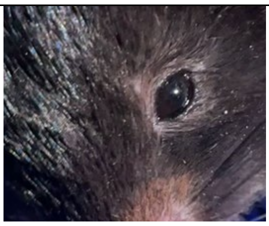<br>Condition: 5XFAD/Control<br>Score = 0.75
Vision loss	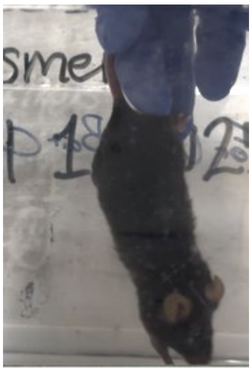<br>One arm extended < 1 cm above floor<br>Score = 0.5	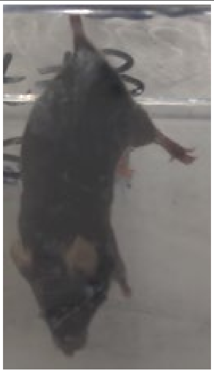<br>Both arms extended > 1 cm above floor<br>Score = 0	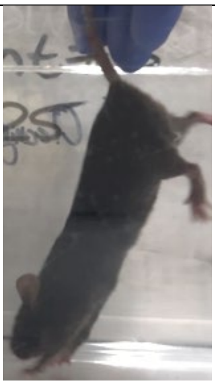<br>Both arms extended < 1 cm above floor<br>Score = 0.25	N/A
Mouse Grimace Scale	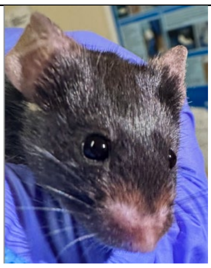<br>Score = 0.25	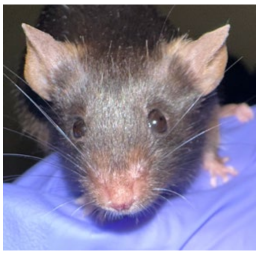<br>Score = 0	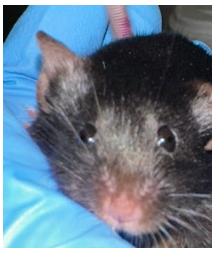<br>Score = 0	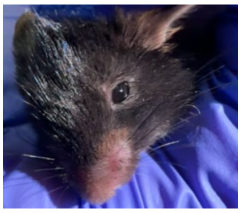<br>Condition: 5XFAD/Control<br>Score = 0.75

Piloerection	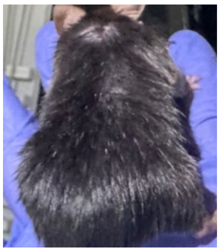 Score = 1	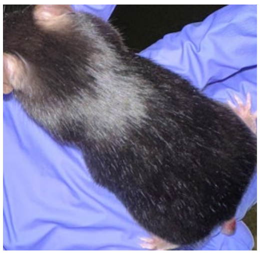 Score = 0.25	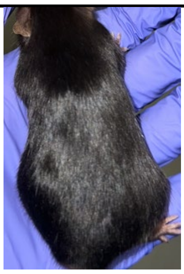 Score = 0	N/A
Body weight score	33.5 g<br>Score = 0	29.6 g<br>Score = 0.5	35.1 g<br>Score = 0	27.8 g<br>Condition: 5XFAD/Control<br>Score = 1
Total score	8.0	3.75	1.25	N/A

(**A**) Note. Data were analyzed using two-way repeated-measures ANOVA with time as the repeated-measures factor and experimental group as the between-subjects factor. Geisser–Greenhouse corrections were applied where appropriate. Bolded values indicate $p < 0.05$. (**B**) Note. Bonferroni-adjusted p values < 0.05 were considered statistically significant and are represented as bolded values. Only regions with significant overall group effects or significant time × group interactions are included. (**C**) Note. The following items have been removed from this exemplary table because all mice scored 0: Tumours, distended abdomen, cataracts, eye discharge/swelling, menace reflex, nasal discharge, malocclusions, rectal prolapse, vaginal/uterine/penile prolapse, diarrhoea, breathing rate/depth. Red lines (dashed or solid) and circles represent points of interest for the given test.

Table S6. (**A**) Paired sample *t*-test summary for total object area exploration on habituation and testing days. (**B**) Spatial (Novel Location Recognition) and non-spatial (Novel Object Recognition) memory test summary of paired sample *t*-test comparing exploration of individual object areas on habituation (Day 6 Trials 1 + 2) vs. test (Day 6 Trials 3 + 4) conditions.

(A)												
			**Female**					**Male**				
			**Trial 1 + 2 Mean ± SEM**	**Trial 3 + 4 Mean ± SEM**	***t*(df)**	***p***	**Cohen′s *d***	**Trial 1 + 2 Mean ± SEM**	**Trial 3 + 4 Mean ± SEM**	***t*(df)**	***p***	**Cohen′s *d***
4 Mo of Age	Day 1 Habituation	5XFAD/Tonic	35.53 ± 6.368	37.232 ± 4.063	−0.236 (6)	0.821	−0.111	32.550 ± 4.233	30.131 ± 3.118	0.582 (7)	0.579	0.215
		5XFAD/Control	27.969 ± 5.673	40.721 ± 5.701	−1.371 (6)	0.219	−0.308	33.799 ± 3.789	31.652 ± 3.224	0.470 (7)	0.653	0.202
		Wild-Type/Control	43.335 ± 4.918	49.296 ± 3.479	−0.843 (7)	0.427	−0.149	36.847 ± 3.279	39.557 4.568	−0.415 (6)	0.693	−0.239
	Day 2 Habituation	5XFAD/Tonic	28.880 ± 1.467	34.260 ± 4.586	−0.929 (6)	0.389	−0.553	23.306 ± 2.561	23.816 ± 2.508	−0.215 (7)	0.836	−0.066
		5XFAD/Control	30.965 ± 3.155	44.342 ± 3.789	−2.579 (6)	**0.042**	−0.572	21.956 ± 1.903	24.362 ± 2.068	−0.855 (7)	0.421	−0.400
		Wild-Type/Control	43.712 ± 4.690	36.616 ± 4.703	1.239 (7)	0.255	0.183	31.058 ± 1.941	30.380 ± 1.471	0.794 (6)	0.458	0.138
	Day 3 Habituation	5XFAD/Tonic	33.375 ± 3.244	31.999 ± 3.305	0.382 (6)	0.715	0.147	22.252 ± 2.257	20.596 ± 4.098	0.485 (7)	0.643	0.166
		5XFAD/Control	35.713 ± 3.105	40.555 ± 3.538	−0.832 (6)	0.437	−0.212	15.044 ± 1.650	15.489 ± 2.309	−0.294 (7)	0.778	−0.073
		Wild-Type/Control	48.479 ± 2.082	38.337 ± 4.766	1.625 (7)	0.148	0.519	18.960 ± 1.621	13.905 ± 3.431	1.297 (6)	0.242	0.659
	Day 4 Habituation	5XFAD/Tonic	28.508 ± 2.926	32.315 ± 3.586	−0.747 (6)	0.484	−0.407	21.336 ± 3.302	21.318 ± 3.133	0.005 (7)	0.996	0.002
		5XFAD/Control	37.150 ± 3.119	41.292 ± 3.807	−1.647 (6)	0.151	−0.179	14.027 ± 1.911	21.817 ± 2.297	−3.543 (7)	**0.009**	−1.219
		Wild-Type/Control	38.842 ± 2.947	35.002 ± 5.258	0.636 (7)	0.545	0.147	23.898 ± 2.534	24.289 ± 2.608	−0.107 (6)	0.918	−0.053
	Day 5 Habituation	5XFAD/Tonic	31.387 ± 3.685	25.480 ± 2.104	1.762 (6)	0.129	0.689	21.310 ± 2.620	26.647 ± 3.674	−1.513 (7)	0.174	−0.553
		5XFAD/Control	32.928 ± 3.443	28.688 ± 3.822	1.094 (6)	0.316	0.168	18.551 ± 1.890	23.917 ± 2.974	−1.927 (7)	0.095	−0.712
		Wild-Type/Control	37.384 ± 5.667	32.124 ± 3.339	1.213 (7)	0.264	0.115	24.714 ± 2.325	22.068 ± 2.465	1.542 (6)	0.174	0.386
	Day 6 Novel Object and Novel Location Recognition Test	5XFAD/Tonic	26.999 ± 4.076	36.386 ± 5.530	−1.264 (6)	0.253	−0.676	21.310 ± 2.473	22.335 ± 1.597	−0.422 (7)	0.685	−0.163
		5XFAD/Control	29.957 ± 4.479	34.662 ± 5.833	−0.910 (6)	0.398	−0.140	14.896 ± 2.960	19.301 ± 1.218	−1.893 (7)	0.100	−0.644
		Wild-Type/Control	23.060 ± 4.232	32.522 ± 4.629	−1.590 (7)	0.156	−0.268	22.993 ± 3.691	22.978 ± 3.813	0.004 (6)	0.997	0.001
7 Mo of Age	Day 1 Habituation	5XFAD/Tonic	20.457 ± 2.851	21.371 ± 2.057	−0.287 (6)	0.784	−0.129	25.859 ± 2.651	32.198 ± 3.725	−2.614 (7)	**0.035**	−0.648
		5XFAD/Control	28.564 ± 2.398	26.253 ± 3.167	2.045 (6)	0.087	0.129	18.792 ± 1.539	24.491 ± 3.230	−1.598 (7)	0.154	−0.745
		Wild-Type/Control	27.798 ± 2.965	25.828 ± 3.760	0.330 (7)	0.751	0.079	30.369 ± 4.870	35.963 ± 6.220	−1.684 (6)	0.143	−0.350
	Day 2 Habituation	5XFAD/Tonic	15.935 ± 1.820	19.837 ± 3.404	−0.897 (6)	0.404	−0.500	30.901 ± 4.910	27.739 ± 2.734	0.606 (7)	0.564	0.263
		5XFAD/Control	17.035 ± 2.358	24.882 ± 2.263	−2.150 (6)	0.075	−0.458	24.247 ± 3.110	24.614 ± 3.608	−0.086 (7)	0.934	−0.036
		Wild-Type/Control	17.117 ± 2.075	17.358 ± 2.222	−0.104 (7)	0.920	−0.014	32.286 ± 6.154	32.960 ± 7.253	−0.228 (6)	0.827	−0.035
	Day 3 Habituation	5XFAD/Tonic	14.614 ± 0.618	14.899 ± 1.591	−0.164 (6)	0.875	−0.083	19.264 ± 2.439	25.777 ± 3.189	−1.774 (7)	0.119	−0.759
		5XFAD/Control	15.343 ± 1.496	22.861 ± 2.317	−2.798 (6)	**0.031**	−0.655	19.597 ± 3.616	21.382 ± 3.845	−0.546 (7)	0.602	−0.158

		Wild-Type/Control	21.752 ± 2.973	25.944 ± 3.083	−1.810 (7)	0.113	−0.170	24.257 ± 3.327	28.349 ± 4.960	−0.925 (6)	0.391	−0.339
	Day 4 Habituation	5XFAD/Tonic	15.988 ± 1.530	15.322 ± 1.915	0.578 (6)	0.585	0.134	27.490 ± 3.892	26.897 ± 3.119	0.141 (7)	0.892	0.056
		5XFAD/Control	19.404 ± 1.651	21.815 ± 3.792	−0.519 (6)	0.622	−0.174	20.393 ± 2.017	28.932 ± 3.667	−2.111 (7)	0.073	−0.954
		Wild-Type/Control	25.151 ± 2.100	22.937 ± 2.779	0.990 (7)	0.355	0.124	31.511 ± 5.396	27.879 ± 4.099	1.458 (6)	0.195	0.265
	Day 5 Habituation	5XFAD/Tonic	13.991 ± 1.310	13.944 ± 1.074	0.039 (6)	0.970	0.014	24.633 ± 3.333	29.062 ± 3.822	−1.300 (7)	0.235	−0.408
		5XFAD/Control	22.286 ± 3.415	18.320 ± 2.451	2.273 (6)	0.063	0.162	23.917 ± 4.173	24.483 ± 2.927	−0.130 (7)	0.900	−0.052
		Wild-Type/Control	25.998 ± 3.572	25.859 ± 3.920	0.036 (6)	0.973	0.005	25.685 ± 3.885	23.750 ± 4.547	0.314 (6)	0.764	0.160
	Day 6 Novel Object and Novel Location Recognition Test	5XFAD/Tonic	12.858 ± 2.120	23.377 ± 3.923	−3.503 (6)	**0.013**	−1.167	26.009 ± 3.871	31.791 ± 4.640	−0.952 (7)	0.373	−0.448
		5XFAD/Control	18.283 ± 1.713	31.331 ± 3.799	−4.484 (6)	**0.004**	−0.917	22.964 ± 2.574	31.680 ± 2.891	−4.361 (7)	**0.003**	−1.053
		Wild-Type/Control	24.010 ± 2.225	39.230 ± 7.032	−2.388 (6)	**0.048**	−0.653	25.517 ± 4.520	27.736 ± 3.453	−0.537 (6)	0.611	−0.193
10 Mo of Age	Day 1 Habituation	5XFAD/Tonic	27.260 ± 3.415	27.503 ± 3.669	−0.071 (6)	0.945	−0.024	21.345 ± 4.288	21.937 ± 3.622	−0.392 (7)	0.707	−0.049
		5XFAD/Control	31.138 ± 3.191	34.192 ± 5.827	−0.531 (6)	0.615	−0.121	18.560 ± 4.428	18.079 ± 3.455	0.162 (7)	0.876	0.040
		Wild-Type/Control	29.991 ± 4.271	32.600 ± 6.533	−0.566 (7)	0.589	−0.071	24.437 ± 5.208	26.721 ± 4.362	−0.580 (6)	0.583	−0.166
	Day 2 Habituation	5XFAD/Tonic	20.250 ± 2.262	24.938 ± 2.117	−1.652 (6)	0.150	−0.749	23.393 ± 1.465	23.483 ± 2.567	−0.032 (7)	0.975	−0.014
		5XFAD/Control	29.949 ± 4.192	32.325 ± 6.335	−0.699 (6)	0.511	−0.074	22.428 ± 2.697	18.607 ± 3.337	2.228 (7)	0.061	0.416
		Wild-Type/Control	31.071 ± 3.538	31.145 ± 2.654	−0.021 (7)	0.984	−0.003	20.503 ± 2.211	24.363 ± 3.749	−0.957 (6)	0.376	−0.439
	Day 3 Habituation	5XFAD/Tonic	18.834 ± 2.871	24.786 ± 2.538	−1.790 (6)	0.124	−0.769	20.115 ± 1.974	22.465 ± 1.913	−1.294 (7)	0.237	−0.400
		5XFAD/Control	25.243 ± 4.826	29.650 ± 4.428	−0.954 (6)	0.377	−0.126	21.586 ± 2.750	22.557 ± 2.413	−0.306 (7)	0.768	−0.124
		Wild-Type/Control	33.109 ± 2.515	29.366 ± 1.367	1.611 (7)	0.151	0.184	23.126 ± 1.641	20.567 ± 4.112	0.633 (6)	0.550	0.286
	Day 4 Habituation	5XFAD/Tonic	15.512 ± 2.695	18.008 ± 3.076	−0.664 (6)	0.531	−0.302	19.439 ± 2.986	24.898 ± 2.727	−1.710 (7)	0.131	−0.631
		5XFAD/Control	23.327 ± 3.863	27.766 ± 4.888	−0.857 (6)	0.425	−0.154	23.261 ± 2.316	20.189 ± 1.758	1.380 (7)	0.210	0.494
		Wild-Type/Control	29.431 ± 2.417	28.154 ± 2.433	0.403 7.000	0.699	0.064	22.354 ± 2.211	26.298 ± 3.430	−1.459 (6)	0.195	−0.478
	Day 5 Habituation	5XFAD/Tonic	18.737 ± 2.596	22.917 ± 2.968	−1.085 (6)	0.320	−0.525	21.995 ± 1.844	19.495 ± 2.133	0.948 (7)	0.375	0.415
		5XFAD/Control	27.934 ± 4.145	36.972 ± 9.365	−1.240 (6)	0.261	−0.261	18.348 ± 2.075	18.727 ± 1.846	−0.107 (7)	0.918	−0.064
		Wild-Type/Control	32.832 ± 2.427	34.597 ± 5.059	−0.278 (7)	0.789	−0.079	21.878 ± 3.263	26.605 ± 4.162	−1.883 (6)	0.109	−0.442
	Day 6 Novel Object and Novel Location Recognition Test	5XFAD/Tonic	15.618 ± 1.872	22.301 ± 2.817	−2.893 (6)	**0.028**	−0.978	16.479 ± 1.621	24.989 ± 2.318	−3.371 (7)	**0.012**	−1.407
		5XFAD/Control	29.290 ± 5.103	37.898 ± 7.546	−1.976 (6)	0.096	−0.222	18.741 ± 2.167	25.228 ± 2.065	−2.214 (7)	0.062	−0.915
		Wild-Type/Control	28.599 ± 2.371	44.751 ± 4.246	−4.200 (7)	**0.004**	−0.768	18.006 ± 2.523	25.615 ± 2.886	−1.814 (6)	0.120	−0.982

(**B**)

			Females					Males				
			**Trial 1 + 2 Habituation Mean ± SEM**	**Trial 3 + 4 Test Mean ± SEM**	***t*(df)**	***p***	**Cohen′s *d***	**Trial 1 + 2 Habituation Mean ± SEM**	**Trial 3 + 4 Test Mean ± SEM**	***t*(df)**	***p***	**Cohen′s *d***
4 Mo of Age	Unmanipulated Object Area	5XFAD/Tonic	13.134 ± 3.520	15.119 ± 4.285	−0.310 (6)	0.767	−0.177	4.876 ± 0.661	3.923 ± 0.733	1.213 (7)	0.264	0.451
		5XFAD/Control	15.946 ± 4.181	17.088 ± 4.493	−0.186	0.858	−0.092	3.840	4.709 ± 0.347	−0.875	0.411	−0.404

					(6)			0.946		(7)		
		Wild-Type/ Control	5.423 ± 2.627	13.478 ± 4.818	−1.638 (7)	0.146	−0.686	4.876 ± 1.455	3.627 ± 0.688	1.128 (6)	0.302	0.384
	Object Area to Receive Moved Object on Novel Location Trial	5XFAD/Tonic	4.325 ± 0.850	3.845 ± 1.023	0.618 (6)	0.559	0.179	3.155 ± 0.642	5.755 ± 1.438	−1.208 (7)	0.266	−0.772
		5XFAD/Control	3.775 ± 1.261	6.207 ± 1.993	−0.753 (6)	0.480	−0.510	2.563 ± 0.775	3.368 ± 0.687	−2.267 (7)	0.058	−0.364
		Wild-Type/ Control	2.662 ± 0.682	3.272 ± 0.747	−0.925 (7)	0.386	−0.282	4.276 ± 0.905	5.065 ± 0.661	−0.715 (6)	0.502	−0.349
	Object Area to be Empty on Novel Location Trial	5XFAD/Tonic	5.256 ± 1.512	10.618 ± 3.138	−1.237 (6)	0.262	−0.762	5.395 ± 0.989	4.737 ± 0.596	0.493 (7)	0.637	0.266
		5XFAD/Control	6.577 ± 1.871	5.255 ± 1.240	0.826 (6)	0.440	0.291	3.821 ± 0.874	3.377 ± 0.929	0.430 (7)	0.680	0.163
		Wild-Type/ Control	8.742 ± 2.036	7.211 ± 2.290	0.596 (7)	0.570	0.234	5.118 ± 1.005	4.885 ± 0.807	0.269 (6)	0.797	0.089
	Object to be Replaced by Novel Object	5XFAD/Tonic	4.283 ± 1.189	6.803 ± 2.055	−1.065 (6)	0.328	−0.525	7.883 ± 1.471	7.920 ± 1.294	−0.020 (7)	0.984	−0.009
		5XFAD/Control	3.659 ± 1.084	6.112 ± 0.823	−1.494 (6)	0.186	−0.892	4.672 ± 1.051	7.846 ± 1.574	−1.729 (7)	0.127	−0.784
		Wild-Type/ Control	6.232 ± 1.871	8.562 ± 2.643	−1.220 (7)	0.262	−0.336	8.723 ± 0.918	9.400 ± 2.844	−0.274 (6)	0.793	−0.112
7 Mo of Age	Unmanipulated Object Area	5XFAD/Tonic	2.749 ± 0.538	5.651 ± 1.112	−2.504 (6)	**0.046**	−1.162	6.014 ± 0.770	7.902 ± 2.251	−0.772 (7)	0.466	−0.371
		5XFAD/Control	5.721 ± 0.752	6.820 ± 0.909	−1.382 (6)	0.216	−0.461	3.932 ± 0.799	6.384 ± 0.727	−3.181 (7)	**0.015**	−1.061
		Wild-Type/ Control	6.255 ± 1.942	9.484 ± 0.777	−1.519 (7)	0.172	−0.722	5.922 ± 1.336	6.641 ± 1.139	−0.322 (6)	0.758	−0.203
	Object Area to Receive Moved Object on Novel Location Trial	5XFAD/Tonic	3.838 ± 0.820	3.595 ± 0.911	0.219 (6)	0.834	0.098	4.182 ± 0.695	5.903 ± 1.547	−0.822 (7)	0.438	−0.475
		5XFAD/Control	4.769 ± 0.447	4.420 ± 0.901	0.357 (6)	0.733	0.172	4.663 ± 1.126	6.652 ± 1.546	−1.014 (7)	0.344	−0.487
		Wild-Type/ Control	5.542 ± 0.716	3.618 ± 0.874	1.762 (7)	0.121	0.797	8.037 ± 2.855	4.557 ± 1.667	1.246 (6)	0.259	0.521
	Object Area to be Empty on Novel Location Trial	5XFAD/Tonic	2.675 ± 0.446	3.704 ± 1.131	−0.799 (6)	0.455	−0.419	6.273 ± 1.220	5.996 ± 2.067	0.103 (7)	0.921	0.054
		5XFAD/Control	3.849 ± 0.765	3.669 ± 0.446	0.182 (6)	0.862	0.100	6.699 ± 1.488	3.830 ± 0.463	1.840 (7)	0.108	0.861
		Wild-Type/ Control	6.430 ± 0.893	4.423 ± 0.888	2.660 (7)	**0.032**	0.745	6.662 ± 1.720	7.032 ± 1.759	−0.392 (6)	0.709	−0.074
	Object to be Replaced by Novel Object	5XFAD/Tonic	3.595 ± 0.957	10.426 ± 3.413	−2.430 (6)	0.051	−0.954	9.539 ± 2.905	11.991 ± 2.874	−0.453 (7)	0.664	−0.281
		5XFAD/Control	3.944 ± 0.946	16.422 ± 3.679	−3.645 (6)	**0.011**	−1.626	7.670 ± 1.692	14.813 ± 2.421	−2.615 (7)	**0.035**	−1.131
		Wild-Type/ Control	5.783 ± 1.100	21.706 ± 7.161	−1.992 (7)	0.087	−1.028	4.896 ± 1.069	9.506 ± 0.899	−2.620 (6)	**0.040**	−1.634
10 Mo of Age	Unmanipulated Object Area	5XFAD/Tonic	4.896 ± 0.788	6.146 ± 1.220	−0.942 (6)	0.383	−0.426	4.269 ± 1.267	4.979 ± 1.402	−0.735 (7)	0.486	−0.176
		5XFAD/Control	12.774 ± 3.203	8.470 ± 1.787	1.537 (6)	0.175	0.581	4.249 ± 1.634	4.283 ± 1.115	−0.021 (7)	0.984	−0.008
		Wild-Type/ Control	11.723 ± 1.478	10.096 ± 1.596	0.880 (7)	0.408	0.350	5.144 ± 0.861	6.651 ± 2.108	−0.749 (6)	0.482	−0.327
	Object Area to Receive Moved Object on Novel Location Trial	5XFAD/Tonic	2.136 ± 0.799	4.506 ± 0.587	−3.019 (6)	**0.023**	−1.183	3.490 ± 1.383	7.192 ± 0.981	−2.761 (7)	**0.028**	−1.021
		5XFAD/Control	3.035 ± 1.129	5.255 ± 1.794	−1.958 (6)	0.098	−0.518	3.147 ± 0.993	7.477 ± 1.017	−3.128 (7)	**0.017**	−1.425
		Wild-Type/ Control	2.248 ± 0.224	7.940 ± 1.536	−3.560 (7)	**0.009**	−1.715	3.242 ± 1.416	5.525 ± 1.013	−2.468 (6)	**0.049**	−0.649
	Object Area to be Empty on Novel Location Trial	5XFAD/Tonic	4.166 ± 0.674	6.124 ± 1.683	−1.279 (6)	0.248	−0.534	3.519 ± 0.819	4.613 ± 0.871	−0.728 (7)	0.490	−0.428
		5XFAD/Control	8.089 ± 1.572	15.523 ± 3.843	−1.800 (6)	0.122	−0.886	4.326 ± 0.822	5.093 ± 0.787	−0.568 (7)	0.587	−0.315
		Wild-Type/ Control	7.504 ± 1.799	17.692 ± 3.666	−3.426 (7)	**0.011**	−1.167	4.119 ± 0.389	3.627 ± 0.723	0.441 (6)	0.674	0.296
		5XFAD/Tonic	4.420 ± 1.232	5.524 ± 1.295	−0.497	0.637	−0.306	5.655 ± 1.136	8.944 ± 1.802	−1.696	0.134	−0.722

				(6)					(7)		
Object to be Replaced by Novel Object	5XFAD/Control	5.393 ± 2.037	8.650 ± 2.541	−2.242 (6)	0.066	−0.495	9.665 ± 1.399	9.459 ± 1.624	0.118 (7)	0.910	0.045
	Wild-Type/ Control	7.124 ± 1.495	9.023 ± 1.208	−1.249 (7)	0.252	−0.462	5.501 ± 0.814	9.812 ± 0.968	−2.934 (6)	**0.026**	−1.687

(**A**) Note. Bolded values represent significance at $p < 0.05$. (**B**) Note. Bolded values represent significance at $p < 0.05$.
